# Recent accelerated diversification in rosids occurred outside the tropics

**DOI:** 10.1038/s41467-020-17116-5

**Published:** 2020-07-03

**Authors:** Miao Sun, Ryan A. Folk, Matthew A. Gitzendanner, Pamela S. Soltis, Zhiduan Chen, Douglas E. Soltis, Robert P. Guralnick

**Affiliations:** 1 Florida Museum of Natural History, University of Florida, Gainesville, FL 32611 USA; 2 Department of Bioscience, Aarhus University, Aarhus, 8000C Denmark; 3 State Key Laboratory of Systematic and Evolutionary Botany, Institute of Botany, The Chinese Academy of Sciences, Beijing, 100093 China; 4 Department of Biological Sciences, Mississippi State University, Starkville, MS 39762 USA; 5 Department of Biology, University of Florida, Gainesville, FL 32611 USA; 6 Biodiversity Institute, University of Florida, Gainesville, FL 32611 USA; 7 Genetics Institute, University of Florida, Gainesville, FL 32608 USA

**Keywords:** Macroecology, Phylogenetics, Adaptive radiation, Plant evolution

## Abstract

Conflicting relationships have been found between diversification rate and temperature across disparate clades of life. Here, we use a supermatrix comprising nearly 20,000 species of rosids—a clade of ~25% of all angiosperm species—to understand global patterns of diversification and its climatic association. Our approach incorporates historical global temperature, assessment of species’ temperature niche, and two broad-scale characterizations of tropical versus non-tropical niche occupancy. We find the diversification rates of most subclades dramatically increased over the last 15 million years (Myr) during cooling associated with global expansion of temperate habitats. Climatic niche is negatively associated with diversification rates, with tropical rosids forming older communities and experiencing speciation rates ~2-fold below rosids in cooler climates. Our results suggest long-term cooling had a disproportionate effect on non-tropical diversification rates, leading to dynamic young communities outside of the tropics, while relative stability in tropical climes led to older, slower-evolving but still species-rich communities.

## Abstract (web summary)

There is mixed evidence for how temperature affects diversification rates. Here, authors use a supermatrix of nearly 20,000 rosid species, comprising almost a quarter of flowering plants, to show that tropical groups are older and speciated twice as slowly as their counterparts from cooler climates.

## Introduction

Attempts to understand the diversification of the angiosperms date to the time of Darwin, yet the drivers of their spectacular species diversity remain to be adequately assessed across much of angiosperm phylogeny as reviewed in Soltis et al.^1^, as well as Sauquet and Magallón^2^. A combination of environmental niche reconstructions and estimates of diversification rates provides one key approach to assess abiotic drivers of angiosperm diversity. Assembly and analysis of such broad-scale data remain challenging^3^. Nevertheless, the identification of climatic drivers of diversification, such as temperature, has seen a surge of recent interest. Numerous studies have demonstrated a correlation between organismal diversification rates and past climate change, particularly with global temperature trends after the Cretaceous-Paleogene (K-Pg) boundary (~65 Million years ago [Myr] to present)^4–8^. The nature of this relationship has been highly variable, with some studies recovering a negative correlation (e.g., accelerated evolution during cool periods^9–11^) and others a positive correlation (rapid evolution during warm periods^6,12^). Likewise, studies of latitudinal gradients in diversification rate have found patterns congruent with both of these relationships (i.e., higher diversification at higher latitudes^13,14^, or higher diversification at lower latitudes^15,16^) or, in some cases, with neither relationship^10,17^. There remains little consensus on the origins of these contrasting biodiversity patterns, with numerous hypotheses proposed across different taxa using different methodological approaches: e.g., the diversification rate hypothesis, the ecological regulation hypothesis, and the tropical conservatism hypothesis^18–20^.

Elucidating the timing and drivers of diversification of the rosid clade of angiosperms is key to better understanding the origin of present-day angiosperm diversity and global vegetation^21^. Rosids, an enormous clade of 90,000–120,000 species (estimated from the Open Tree of Life [OpenTree] and Open Tree Taxonomy [OTT] database^22^), represent more than a quarter of all angiosperms, based on an estimated 400,000 species of angiosperms^23^. The clade originated in the Early to Late Cretaceous (115–93 Myr), followed by rapid diversification of crown groups (sensu APG IV^24^) of fabids (112 to 91 Myr), and malvids (109 to 83 Myr^25,26^) in perhaps as little as 4–5 million years^25^.

Present-day angiosperm-dominated forests are the outcomes of the rapid initial rise of the rosid clade and its subsequent repeated cycles of radiations^25,27^. These forests also profoundly shaped many current terrestrial ecosystems and much of biodiversity^25,28^. Many other lineages of life radiated in the shadow of rosid-dominated angiosperm forests (e.g., ants^29^, amphibians^30^, mammals^31^, liverworts^32^, and most ferns^33^). Rosids are also highly diverse ecologically (e.g., parasite: Rafflesiaceae; mangrove: Rhizophoraceae; aquatic: Podostemaceae) and now occupy most terrestrial habitats (e.g., arctic, alpine, desert, temperate, and tropical forests) with correspondingly large trait diversity (e.g., chemistry, reproductive strategy, and life history^25,34,35^).

Rosids have their highest species richness in the tropics, where they are ecologically dominant^36^, but most rosid subclades extend into subtropical and temperate areas and climate zones^25^, and some lineages, including Brassicaceae, Rosaceae, and Fagales, greatly diversified in temperate habitats^11,37,38^. Despite recent efforts to develop comprehensive rosid phylogenies^25,39–41^, no large-scale assessment of rosid diversification has yet been conducted, likely due to the formidable size of this clade. However, extensive phylogenetic sampling in such large clades is valuable for providing a broad context and clade-level replication for testing macroevolutionary hypotheses^10,36,42^. The enormous diversity of rosids therefore affords the opportunity for evolutionary replication across multiple globally distributed clades, whereas latitudinal diversity disparities enable testing how climate, and temperature in particular, may have driven diversification.

Here we use a recently compiled 5-locus phylogenetic tree for rosids^41^, with more than twice the taxon sampling used in earlier studies^39,40^, to examine how past climates have driven diversification. Using the 17 rosid orders (clades classified sensu APG IV^24^) as replicated evolutionary tests, we asked whether latitudinal and climatic disparities in rosid diversity are due to (1) differences in diversification rates of contemporary species and communities within and outside of the tropics; (2) historical temperature-dependence of the diversification process; or (3) differences in species ages in tropical versus non-tropical communities. In particular, we consider both geographic and climatic definitions of tropicality (sensu Owens et al.^43^) and use emerging theory linking climatic niche conservatism to increased diversification rates in habitats outside the tropics^19^. We find that rates of speciation are about twofold higher in non-tropical rosid communities, which are also typically younger. This surprising finding likely relates to marked increases in diversification rates of most rosid subclades during cooling associated with the global expansion of temperate habitats in the Miocene. Our approach not only addresses key, long-held diversity gradient hypotheses, but also considers multiple lines of evidence toward a more process-oriented view of species and diversification gradients.

## Results

### Species record accumulation

We assembled occurrences for all name-validated rosid species via queries using R packages rgbif v1.3.0^44^ and ridigbio v0.3.5^45^, as discussed in the Methods and Supplementary Method 1. Of the 19,740 sampled rosid species, 18,283 species initially had occurrence data in iDigBio and GBIF; we removed species lacking geographic coordinates, geographic outlier records (Methods and Supplementary Method 1), and coordinates where both latitude and longitude were estimated only to the nearest integer degree. After these cleaning steps, 2,691,099 records remained, and each species had an average of 160 occurrence points. We then paired the species with data from the three contemporary niche data sets: mean annual temperature (MAT; 16,986 species passed our filters), geographic tropicality (18,269 species passed), and Köppen–Geiger climatic tropicality (17,635 species passed). See Supplementary Table 1 for detailed summary of each data layer for the 17 rosid orders.

### Diversification and current temperature niche

Although we fit a variety of pure-birth and birth-death models, we conservatively chose to focus on reporting speciation parameters, given the challenge of estimating extinction rates from extant-only phylogenetic data and that extinction is probably systematically underestimated^46–48^. Hereafter, without qualification we refer to diversification rate (that is, net diversification estimated from birth-death models including both speciation and extinction rates) or to speciation rates estimated from birth-death models, but for trait correlation tests we used present speciation rates (hereafter, tip rates) estimated from DR (diversification rate^17^) and BAMM (Bayesian analysis of macroevolutionary mixtures^49^, and STRAPP^50^; see Methods and Supplementary Method 1).

**Fig. 1:**
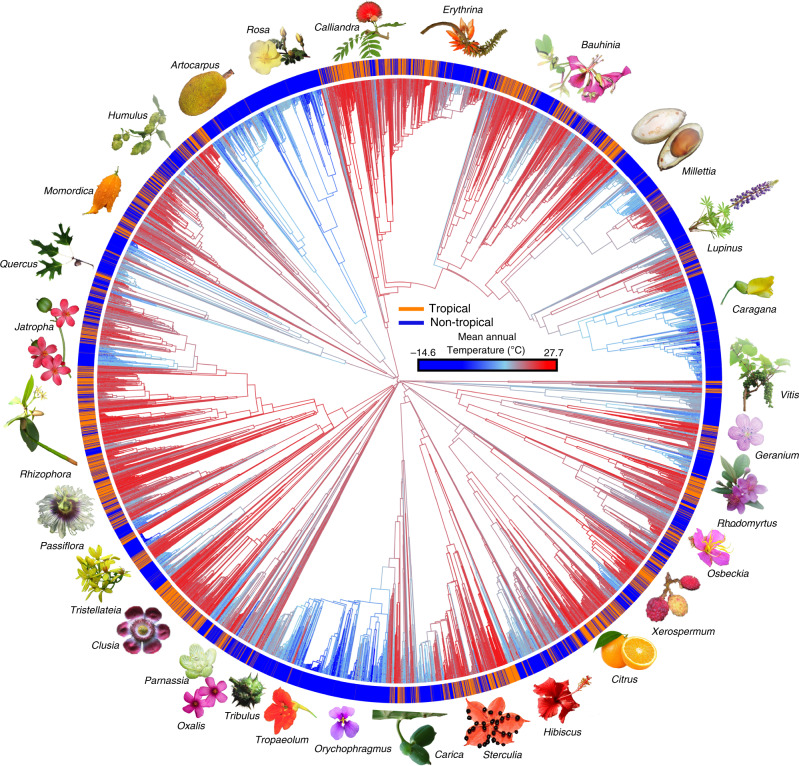
Ancestral reconstruction across the rosid tree for mean annual temperature. Branches are colored based on mean annual temperature from low (−14.6 °C; blue) to high (27.7 °C; red) values. Species tips are colored by the Köppen–Geiger climatic classification^43^; orange represents tropical species, and blue represents non-tropical species, based on climatic criteria; we also represent rosid diversity using icons (flower, fruit, leaves) of clade members to conceptually reflect their morphological character diversity, tropicality, and economic importance; a genus name is provided with each associated icon.

We tested for a correlation between tip rates and MAT using es-SIM^51^ and STRAPP^50^. We found a significant relationship between current temperature niche and tip rates across all rosids using es-SIM (two-tailed *p* = 0.00067, test statistic described in Harvey & Rabosky^51^; Table 1, Fig. 1), although the correlation between tip rates and MAT was not significant using STRAPP (Supplementary Table 3). The direction of this relationship was negative (that is, lower temperatures were associated with higher tip rates and vice versa; *ρ* = −0.29186, Table 1). Among the 17 rosid orders (sensu APG IV^24^; Table 1), only Malpighiales showed a significant relationship and only using es-SIM (*p* = 0.01133), but there was a strong overall trend towards a negative correlation (*ρ*) between tip rates and temperature (*t*-test of zero mean difference; two-tailed *p* = 0.00039); Celastrales and Geraniales were the only two clades with a positive correlation between climate and tip rates (Table 1). We also tested for phylogenetic conservatism of temperature niche with Pagel’s *λ* test^52 ^(here we use phylogenetic signal as the criterion of phylogenetic niche conservatism^53^; see Supplementary Method 2). We found very strong evidence for phylogenetic signal in MAT (Likelihood ratio test: *p* ~ 0 for Pagel’s *λ* test = 0.86209 across the entire rosid tree; Supplementary Table 2).

**Table 1:** Summary table of semi-parametric tests for correlation between diversification rate and continuous mean annual temperature (es-SIM).

	es-SIM		
Order	Rho (*ρ*)	*p*-value	pv.adjust
Brassicales	−0.3945	0.0180	0.2699
Celastrales	0.0185	0.9444	0.9444
Crossosomatales	−0.1255	0.8011	0.9444
Cucurbitales	−0.0471	0.9290	0.9444
Fabales	−0.3290	0.0060	0.0960
Fagales	−0.0767	0.4985	0.9444
Geraniales	0.0655	0.8117	0.9444
Huerteales	−0.6484	0.2413	0.9444
Malpighiales	−0.3256	0.0007	**0.0113**
Malvales	−0.1251	0.4392	0.9444
Myrtales	−0.2129	0.1113	0.9444
Oxalidales	−0.1205	0.5252	0.9444
Picramniales	−0.3169	0.7711	0.9444
Rosales	−0.2072	0.2186	0.9444
Sapindales	−0.0668	0.5012	0.9444
Vitales	−0.3762	0.1619	0.9444
Zygophyllales	−0.0581	0.7804	0.9444
Total tree	−0.2919	**0.0007**	/

The significant *p*-values for adjusted results are in bold. Rho (*ρ*) is the Pearson’s correlation coefficient; *p*-value is for the correlation between mean annual temperature and speciation rate; pv.adjust refers to adjusting the corresponding *p*-value for family-wise error using the Hochberg^97^ method (which can result in identical adjusted *p*-values as implemented in *R*). Rosid orders are provided, sensu APG IV^24^.

### Diversification and measures of tropicality

Climatic and geographic definitions resulted in strongly differing delimitations of tropicality. Although we found approximately equal numbers of non-tropical and tropical species with a geographic definition (51.45% and 48.55%, respectively; Supplementary Table 1), climatic criteria resulted in an increased number of non-tropical species (65.07% non-tropical; 34.93% tropical; Supplementary Table 1). The apparent disparities between geographic and climatic tropicality criteria are in subtropical and tropical-montane regions^43^, likely driving the scoring difference. Under the geographic definition, only 27% of the species had at least one point occurrence in both non-tropical and tropical areas. Most species that span both areas have almost all points in one of them, and the majority of species (73%) are found solely in non-tropical or tropical areas. We discuss temperate–tropical biases in more detail below. Under either definition, there was again strong evidence for phylogenetic conservatism of temperature niche using Pagel’s *λ* test (geographic: *λ* = 0.93815, Likelihood ratio test one-tailed, *p* = 2.61e−306; climatic: *λ* = 0.95630, Likelihood ratio test one-tailed, *p* = 1.25e−244; Supplementary Table 2).

We tested for a relationship between climatic tropicality and tip rates with two methods: one using semi-parametric tip rates (FiSSE^54^) and the other using tip rates estimated in BAMM (i.e. STRAPP^50^). We found a significant relationship (FiSSE: two-tailed *p* ~ 0, test statistic described in Rabosky & Goldberg^54^; Table 2); the average tip rate of species outside the tropics was approximately twofold that of tropical species (*λ*_non-tropical_ = 0.5047; *λ*_tropical_ = 0.2552, as estimated in FiSSE; Table 2). Among the 17 orders, climatic tropicality was significantly associated with tip rates for Fabales (in both tests, negatively: *λ*_non-tropical_ > *λ*_tropical_): FiSSE (*p* = 0.02997; Table 2) and STRAPP (two-tailed *p* ~ 0, test statistic defined in Rabosky & Huang^50^; Supplementary Table 3). Estimated tip rates for non-tropical species (*λ*_non-tropical_ as estimated by FiSSE) were consistently and significantly different from those of tropical species (*λ*_tropical_) across orders (Table 2), and this difference was significant (*t*-test for mean difference: two-tailed *p* = 0.0110). However, there was not a significant relationship between the percentage of non-tropical species in an ordinal clade and the disparity between non-tropical and tropical tip rates (linear model of non-tropical proportion vs. *λ*_non-tropical_ /*λ*_tropical_; *F*-test: two-tailed *p* = 0.8012), indicating that this relationship existed regardless of overall clade temperature niche.

**Table 2:** Summary of correlation tests between tip rates and the two binary tropicality data sets in FiSSE.

	Köppen–Geiger				Geographic			
Order	*λ*_non-tropical_	*λ*_tropical_	*p*-value	pv.adjust	*λ*_non-tropical_	λ_tropical_	*p*-value	pv.adjust
Brassicales	0.5198	0.1594	0.0985	0.5554	0.5079	0.4410	0.4865	0.7682
Celastrales	0.2208	0.2015	NA	NA	0.2309	0.2046	NA	NA
Crossosomatales	0.0776	0.1073	0.6104	0.6104	0.0893	0.0477	0.2248	0.7682
Cucurbitales	0.3033	0.2353	0.2168	0.5554	0.2919	0.2843	0.4945	0.7682
Fabales	0.6197	0.2946	0.0020	**0.0300**	0.6962	0.3450	0.0080	0.0959
Fagales	0.6032	0.3385	0.0320	0.4156	0.6276	0.4008	0.0609	0.6703
Geraniales	0.2919	0.0544	0.1578	0.5554	0.2988	0.2366	0.2378	0.7682
Huerteales	0.0278	0.0195	0.1718	0.5554	0.0278	0.0195	0.1768	0.7682
Malpighiales	0.4105	0.2312	0.0230	0.3217	0.4747	0.2582	0.0060	0.0779
Malvales	0.4066	0.2963	0.1898	0.5554	0.4305	0.3639	0.3666	0.7682
Myrtales	0.4766	0.2594	0.0440	0.4835	0.4972	0.3544	0.3437	0.7682
Oxalidales	0.1532	0.0854	0.1698	0.5554	0.1815	0.1503	NA	NA
Picramniales	0.0875	0.0488	0.1389	0.5554	NA	NA	NA	NA
Rosales	0.6407	0.3218	0.0390	0.4675	0.6795	0.4339	0.1149	0.7682
Sapindales	0.2576	0.2202	0.1159	0.5554	0.2815	0.2280	0.1089	0.7682
Vitales	0.7599	0.1055	NA	NA	0.9223	0.1014	NA	NA
Zygophyllales	0.0628	0.0537	0.2777	0.5554	0.0570	0.0663	0.7682	0.7682
Total tree	0.5047	0.2552	**0**	/	0.5552	0.3089	**0**	/

The significant *p*-values for adjusted results are in bold; *p*-value is for the correlation between the Köppen–Geiger climatic/Geographic tropicality and tip rate; pv.adjust refers to adjusting the corresponding *p*-value for family-wise error using the Hochberg^97^ method. For the binary data, computation of some *p*-values in small subclades failed due to monomorphic traits or mismatched transition rates, which are marked as “NA”. Rosid orders are provided, sensu APG IV^24^.

A geographically non-tropical distribution was also significantly associated with different tip rates across the rosids (FiSSE: two-tailed *p* ~ 0; Table 2); geographically non-tropical species had ~1.8-fold the tip rate of tropical species (*λ*_non-tropical_ = 0.5552; *λ*_tropical_ = 0.3089; Table 2). However, this association was weaker than for climatic tropicality, with no order showing a significant association of geographic tropicality with diversification and no significant difference across rosid orders between average tip rates (*λ*_non-tropical_ and *λ*_tropical_; *t*-test: two-tailed *p* = 0.0545; Table 2). Similarly, as with climatic tropicality, there was no relationship between the percentage of non-tropical species in the order and the magnitude of the difference between non-tropical and tropical tip rates (linear model of non-tropical proportion vs. *λ*_non-tropical_ /*λ*_tropical_; *F*-test: two-tailed *p* = 0.4151).

We also assessed the relationship between tropicality and diversification under a fully model-based framework while accounting for the possibility of an unobserved trait driving diversification using a series of HiSSE (hidden-state speciation and extinction^55^) and BiSSE (binary state speciation and extinction^56^) models (Supplementary Method 3). We implemented these tests under a model comparison framework using AIC (Akaike information criterion^57^). Under both definitions of tropicality (climatic and geographic), we find full support with AW (Akaike weight^58^; AW ~ 1) for a full HiSSE model, with equal transition rates and relative extinction among tropicality states, but different diversification rates between these two states (Supplementary Table 4). That is, tropicality is significantly associated with diversification, but in concert with an unobserved trait that also drives diversification. Non-tropical diversification is higher under one of these “hidden” traits as expected, but under the other hidden trait, it is slightly (Köppen–Geiger tropicality) or substantially (geographic tropicality) lower. This indicates that, although the overall diversification rate is higher for non-tropical species, the direction of this effect is modulated by an unobserved trait.

Sampling across orders was idiosyncratic with respect to tropicality. For instance, the most prevalently tropical large order, Malpighiales (52.8% climatically tropical, 17.37% species coverage in the tree; cf. Table 1 in Sun et al.^41^), was far better sampled than Rosales, one of the most prominent temperate radiations in the rosids (13.3% tropical; 8.22% species coverage). However, our species richness plots suggest overrepresentation of Rosales in temperate North America and Europe (Supplementary Fig. 1). To test how much both phylogenetic and occurrence record sampling biases might impact predictions of tip rates at the ordinal level, we performed sensitivity analyses (Supplementary Method 4). Based on a series of random taxon dropping treatments representing different disparities of sampling effort among tropical and non-tropical species, we find that overall estimated tip rates are largely insensitive to these sampling differences (Supplementary Method 4 and Supplementary Table 5). Consistent with this finding, although sampling of ordinal trees was indeed moderately negatively correlated with the percent of species that were tropical (*ρ* = −0.2029), this trend was only marginally significant (*F*-test: two-tailed *p* = 0.0397). Likewise, the percentage of each order sampled showed no relationship with the disparity between tropical and non-tropical tip rates (i.e., *λ*_non-tropical_ /*λ*_tropical_; *ρ* = −0.0442; *F*-test: two-tailed *p* = 0.5779). These findings, combined with recovery of largely congruent correlations across clades with different levels of tropicality (above), suggest that the negative diversification-temperature relationship we recovered is robust globally across the phylogeny and independent of sampling effort.

**Fig. 2:**
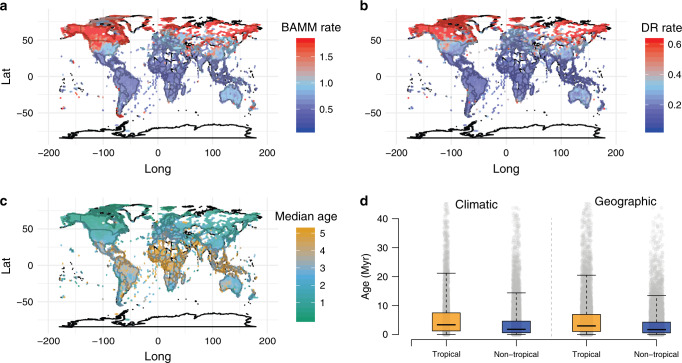
Global distribution patterns of median tip rates and median species age. Tip rates estimated by BAMM (**a**) and DR (**b**) (red = faster, and blue = slower); species tip age (**c**) is defined as the age of the parent node for any given tip and reported as the grid cell median (orange = older; green = younger); **d** boxplot of median species age for tropical (orange) and non-tropical (blue) species under both climatic and geographic definitions. *t*-test: both two-tailed; climatic *p* = 3.5518e−68 and geographic *p* = 2.3176e−82; median—solid line in the box, box—interquartile range [25% and 75%], whiskers—5% and 95% intervals; *n* = 6160, 11,475, 8870, and 9399, respectively; see gray dots for data distribution. All values in **a**–**c** are plotted in equal-area grid cells (~322 km^2^); source data are provided in Source Data file.

By mapping median tip rates from BAMM and DR analyses on equal-area grids (Fig. 2a, b), we reveal a strong latitudinal pattern in diversification rate specific to the Northern Hemisphere. As with tropicality state-specific estimates, northern non-tropical communities have approximately twofold the median tip rate as communities in tropical areas. Consistent with this pattern, we also show that tropical communities contain substantially older rosid species than communities outside the tropics under both climatic and geographic definitions (Fig. 2c, d; *t*-test: both two-tailed; climatic *p* = 3.5518e−68, and geographic *p* = 2.3176e−82).

### Diversification and global historical temperature

We used the Sun et al.^41^ rosid tree with global paleo-temperature data^59^ and RPANDA^60^ to test alternative diversification models, with more details discussed in Methods below and in Supplementary Method 5. Model selection demonstrated that temperature-dependent models were favored without exception (Supplementary Table 6). In all 17 orders, the optimal model had constant speciation and extinction with respect to temperature. This result indicates a proportional relationship of diversification with temperature; the coefficients of the models indicated the expected negative relationship. The AW for this model was ~1 with the exception of the two smallest orders (Huerteales, 30 species with 28.57% tropical species, AW = 0.768; Picramniales, 57 species with 100% tropical species, AW = 0.812; also see Supplementary Tables 1 and 6).

**Fig. 3:**
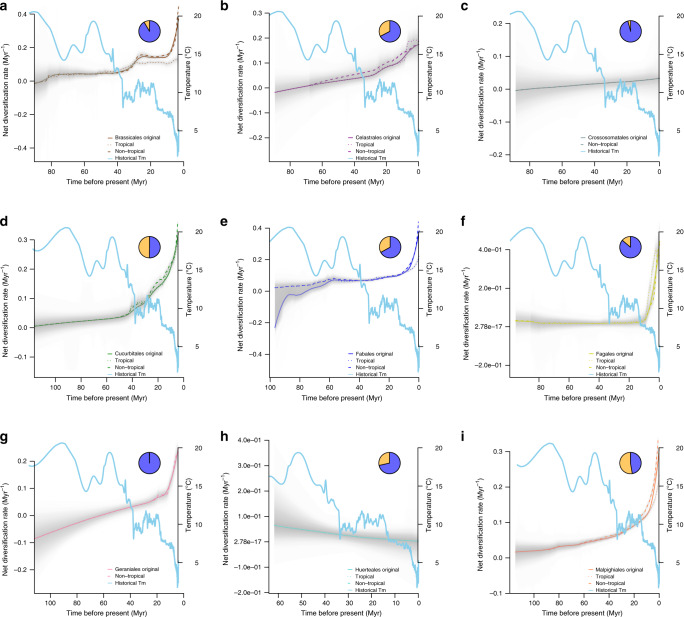

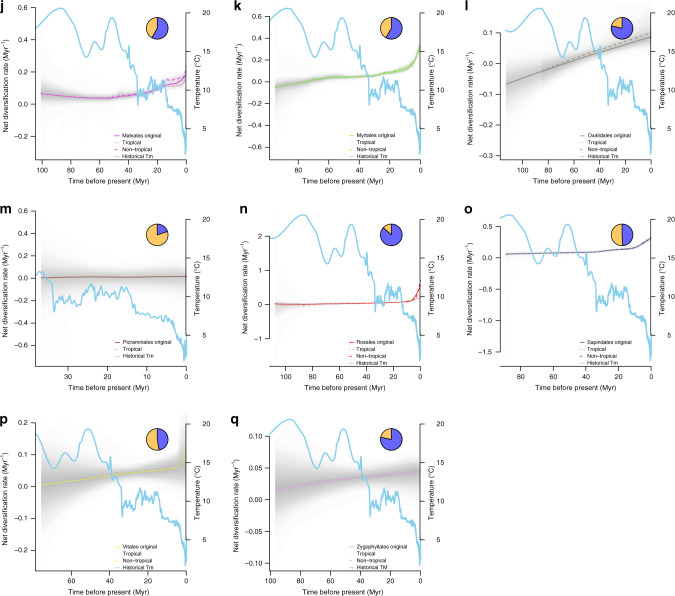
Trends in net rosid diversification rate-through-time plots of tropical and non-tropical lineages against global temperature. Net diversification rate curves were generated using BAMM for each of the 17 rosid orders (clades) (**a**–**q**); each curve is a median line colored by order; the solid line is the original net diversification rate curve, the dotted line represents the rate of tropical lineages, and the dashed line represents the rate of non-tropical lineages (no curves displayed for any lineage with sample number < 3). The gray segment is the 95% confidence interval; the sky blue line is the trend of global temperature change since the Late Cretaceous (~113 Myr), plotted as a 5-point mean derived from Cramer et al.^59^; the pie-chart represents the percentage of tropical (orange) and non-tropical (blue) species within each order summarized from the Köppen–Geiger climatic classification^43^. Each timescale (*x* axis) is rescaled to the clade age.

We also found strong evidence for a negative correlation between estimated global paleo-temperature data and tree-wide net diversification rates (here, not tip rates) using BAMM^49^. In most orders, the association between temperature and net diversification rate was negative and linear (model choice based on AIC against an exponential model; median *R*^2^ = 0.712; Supplementary Fig. 2). The exception was Huerteales, the smallest rosid order (30 species), which exhibited a positive correlation (Supplementary Fig. 2h). Across subclades, more than half of the diversification shifts (97/182 in total, summed over all 17 orders) were detected from 10 to 15 Myr to present, during which the global temperature was cooling, whereas <5 shifts were detected at the Eocene Thermal Maximum (~55 Myr) when the global temperature was more than 5–8 °C above average. Moreover, among most rosid orders, rate accelerations occurred primarily from ~10 to 15 Myr to the present, corresponding to the end of the mid-Miocene Climatic Optimum (Fig. 3; Supplementary Fig. 3). Such increased rates were also evident in some clades (Brassicales, Celastrales, Cucurbitales, and Sapindales; Fig. 3a, b, d, o) closer to an earlier cooling period at the Eocene-Oligocene Glacial Maximum (~34 Myr). Five orders showed no signs of recent bursts of diversification and no shifts in diversification rate detected by BAMM (Crossosomatales, Huerteales, Oxalidales, Picramniales, and Zygophyllales; Fig. 3c, h, l, m, q). This finding may represent a statistical power issue, as four of these five orders are the smallest orders of rosids (Supplementary Table 1).

The response of individual rosid orders to global historical climate change was idiosyncratic, but in general most clades experienced substantial diversification rate shifts and increases from ~10 to 15 Myr to the present (Fig. 3; Supplementary Fig. 3). For instance, Fagales maintained a relatively constant net diversification rate close to zero from its origination (~95.6 Myr) to ~10 Myr and then exhibited a marked increase to the present (Fig. 3f). The diversification rate curve of Rosales was relatively flat after 107 Myr; then there was a rate drop at ~90 Myr, followed by an abrupt rate increase from ~15 Myr (around the Middle Miocene Climatic Optimum) to the present (Fig. 3n). Only a few very small orders exhibited an approximately constant (Picramniales; all tropical species; Fig. 3m) or decreasing (Huerteales; Fig. 3h) trend in diversification towards the present.

## Discussion

Species richness of rosid communities is higher in tropical regions than elsewhere on the planet^36^ (Supplementary Fig. 1), and yet we found that tip rates are about twofold higher in non-tropical rosid species (Fig. 2a, b; Table 2). This pattern is only marginally weaker under geographic definitions of tropicality. Individual rosid orders sometimes showed much greater disparities, such as Vitales (7.2-fold higher for climatically non-tropical species; 9.1-fold higher for geographically non-tropical species). Part of the explanation for this startling pattern may be attributed to our finding of a strong negative relationship between diversification (or tip rates) and both historical and present-day temperature. This association is present both globally across all rosids and in rosid subclades, and is robust to several analytical methods, sensitivity tests, and several data sets representing temperature-species associations. Similar patterns of increased diversification with global cooling have been found in mammals^9^, birds^7^, and some other flowering plant lineages^10,11,14,38^. Both falling global temperature and aridification are compatible with the timing of diversification reconstructed here for rosids and most rosid subclades (~10–15 Myr to the present), a timeframe that also corresponds to the origin of many modern-day temperate plant communities^61^. Consistent with this observation, we found support for hidden-state models of tropicality (Supplementary Table 4), indicating a role for unobserved drivers of diversification that may work in concert with temperature and should be the focus of future study.

Despite concordant overall patterns in the diversification–climate relationship across most rosid orders, rate-through-time plots demonstrate a diversity of responses to global temperature (Fig. 3). These differences among subclades may reflect phylogenetic constraints imposed by ancestral temperature niche and other factors^62^. Nevertheless, temperature is negatively associated with diversification not only in largely temperate clades such as Rosales and Fabales, but also in large clades such as Malpighiales, in which more species diversity is tropical (52.76% are tropical under more conservative Köppen–Geiger definitions^43^; 67.21% under geographic definition; see Supplementary Table 1); even in clades that are primarily tropical, tip rates tend to be highest in non-tropical species (Fig. 2a, b). Although the estimated direction of the temperature–diversification relationship was consistent across analyses, relationships were not significant for many orders. This finding is likely due to a loss of power resulting both from corrections for multiple comparisons and from subtrees with limited numbers of tips^50,51,54,63–65^, and perhaps most importantly, the limited information that can be extracted when combining extant-only phylogenetic data with a single univariate trait^65^.

We found compelling evidence of a negative relationship between temperature and diversification in the rosids. These results are part of an emerging pattern in large-scale diversification studies^10,13,14,66^ (but see refs. ^15,16^) suggesting that non-tropical species exhibit high diversification rates despite the lower species richness of communities outside of the tropics. Our results are consistent with recent work that examined speciation rates across geographic space in angiosperms and showed lower angiosperm speciation in the tropics^14^. Our work significantly extends this effort by explicitly considering multiple definitions of tropicality, as well as the relationship between temperature and diversification over geological time and across multiple subclades. Our diversification results challenge some explanations of high species diversity in the tropics invoking higher tropical diversification rates^21,67^ (also reviewed in Donoghue^68^). The negative correlation between species richness and diversification may instead suggest little turnover of ancient, species-rich communities in the tropics (the tropical conservatism hypothesis^18,66^) in contrast to the dynamism of high-latitude biomes in the recent past (the last 15 million years^10,13,69–71^). We note, however, that the tropics themselves are heterogeneous and that seasonally dry tropical forests are poorly understood but may form exceptions to the diversity gradient patterns found to date.

We determined clade ages across gridded rosid communities and found that the tropics generally contain older rosid communities (Fig. 2c, d) compared to younger communities in the high latitudes of the Northern Hemisphere. Those same communities also possess species with the highest diversification rates (Fig. 2) of anywhere else on the globe. Our finding of diversification rate differences between non-tropical areas of the Northern and Southern Hemispheres has been observed before^72^ and is likely due to the greater historical climatic stability of the biomes of the Southern Hemisphere. That same dynamism suggests that extinction rates in especially temperate and polar regions may also be higher^73^, as both global cooling and perturbations during the Pleistocene likely impacted those regions more strongly. Because extinction rates are challenging to estimate from extant-only phylogenetic data^46–48^, we conservatively chose to focus on reporting speciation parameters (i.e., tip rates; but see net diversification reported in Fig. 3 and Supplementary Fig. 2).

Ecological opportunity due to Earth’s dynamic climatic past has been frequently invoked to explain high diversification rates in species currently inhabiting temperate^14,69–71,74^ and arid^62,75^ environments. Significantly, these studies do not typically invoke adaptive radiations. Rather, these diversifications appear to rely on a passive mechanism via bursts of speciation for lineages already well-adapted to these conditions, and in some cases acquisition of cold-tolerant traits quite clearly predates diversification^10,69^. Among the most important traits in temperate and cold environments, and a key, previously documented constraint on plant communities and flowering plant evolution is freezing tolerance (reviewed in Zanne et al.^76^). Freezing tolerance is underlain by a restricted set of traits^76,77^ that has evolved many times via exaptation from existing vegetative variation^78^. Despite its many origins, freezing tolerance has been gained in fewer than half of all flowering plant families from their probable tropical ancestor^76,79,80^. Our results are consistent with supposition of pre-existing cold-tolerant traits across most rosid lineages, all of which are far older than the onset of cold climates in the Plio-Pleistocene. This was followed by a largely passive diversification in response to the strong spatial expansion of extra-tropical habitats beginning in the Miocene and mediated by what appears to be strong niche conservatism of cold-tolerant or intolerant traits^68^. This finding is, in essence, a refinement of the tropical conservatism hypothesis (first proposed by Wiens & Donoghue^18^). Further work on developing climatic layers over time that can trace the expansion of strong seasonality, and especially areas with seasonal freezing temperatures is a key next step. These layers, when coupled with more fine-grained, modeled past and present species distribution information would provide a strong basis for more explicit testing of this more mechanism-focused hypothesis.

Although our taxon sampling was extensive (~20,000 species), we recognize that our sampling of the enormous rosid clade (90,000–120,000 species) remains limited, introducing possible biases. However, these biases are inherent in any large-scale biodiversity analyses^36,41^. For instance, differences in sampling rate between tropical and non-tropical species could be important for estimating niche-specific speciation rates. Significantly, we found surprisingly little impact of sampling rates on the strong and repeated trends we observed across well-sampled clades, a robustness that is likely due to the relatively weak association of incomplete sampling with phylogeny^81^ and with niche tropicality in this data set. Methods for modeling incomplete clade sampling do not replace the role of molecular phylogenetic data^82,83^. Lastly, fully incorporating estimates of phylogenetic uncertainty in a data set of nearly 20,000 species is currently computationally intractable with the models used here. However, in our previous work^41,83^, we have extensively studied the properties of this phylogenetic data set with respect to phylogenetic and diversification analyses. In particular, we performed a series of experiments on the properties of RPANDA and BAMM on the current rosid phylogenetic estimate and found diversification estimates to be robust on two supermatrix trees differing strongly in sampling and phylogenetic support^83^ (see also similar sampling experiments in Igea & Tanentzap^14^). We believe our exploration of phylogenetic uncertainty is representative of current approaches given the phylogenetic scale considered.

Despite the challenges of computational tractability and sampling for megatrees, our work here still provides a baseline set of hypotheses to test further, with yet more robust sampling and improving tools, methods, and resources. Future studies on large phylogenetic, niche, and trait data sets such as those we have started to build in rosids will be critical for further identifying and refining drivers of the high diversity of rosids in many contemporary terrestrial habitats and potentially predicting how these key members of present-day plant communities may respond during future, rapid climate warming.

## Methods

### 5-Locus rosid supermatrix

We used a tree generated from a supermatrix of 19,740 species of rosids^41^ for diversification analysis. This is the most comprehensive existing phylogeny for the rosid clade. Briefly, to construct this tree, Sun et al.^41^ mined GenBank for commonly used genetic regions using the PHyLogeny Assembly With Databases pipeline^84^ (PHLAWD, version 3.4a, https://github.com/blackrim/phlawd). The regions used represent all three plant genomes, including the chloroplast genes *atpB*, *matK*, and *rbcL*, the mitochondrial gene *matR*, and the nuclear ribosomal ITS (i.e., ITS-1, 5.8S, and ITS-2 regions). We validated the species names following The Plant List (http://www.theplantlist.org/) and removed invalid names and any taxon names with “sp.”, “subsp.”, “var.”, “x”, “cf.” and “aff.”, or other non-species designations; we curated the 5-locus data set iteratively by screening individual loci and concatenated matrices for rogue taxon behavior through manual inspection of initial trees; detailed cleaning steps are fully discussed in Sun et al.^41^. After cleaning, we concatenated the loci and inferred the best ML tree with RAxML^85^ using the extended Majority Rule Criterion (autoMRE) as a bootstrap stopping rule^86^ (reached at 352 replicates). The final 5-locus data set contained 19,740 ingroup species (135 families and 17 orders) and 554 outgroup species; the latter were excluded from downstream diversification analyses.

Divergence time estimation was conducted in treePL v.1.0^87^ using 59 calibration points covering 15 of the 17 rosid orders^24,41^ with a root constraint following Wang et al.^25^. For determining community age (Fig. 2c, d), we defined the age of a tip species as the age of its closest internal tree node and reported community-level ages as the grid cell median using the R package dggridR v. 2.0.3^88^. We then used both climatic and geographic definitions of tropicality in order to test for an age difference between tropical and non-tropical species (Fig. 2d).

We assessed the quality of the final tree by comparing the topology and recovered clade ages to a series of previous benchmark studies^26,39^ and compared sampling statistics to the OTT taxonomy^22^. More details of tree quality control are discussed in Sun et al.^41,83^. Overall, we found broad congruence with previous results (reviewed in Sun et al.^39,41^), but with increased resolution in many areas of the phylogeny enabled by denser taxon sampling. Sampling remains incomplete (also see Supplementary Table 7), but this phylogeny represents a strong improvement for the clade with more than twice the coverage of previous efforts (reviewed in Sun et al.^41^).

Given both our motivation to use the rosid clade for evolutionary replication of diversification patterns and its prohibitive size for many diversification methods, we split the phylogenetic tree into 17 subclades corresponding to all rosid orders recognized in APG IV^24^. Likewise, for computational feasibility, all analyses were run in parallel across 17 order subtrees, unless otherwise stated.

### Assembly of species distribution data

We queried all rosid species sampled in our tree from iDigBio and GBIF using R packages rgbif v1.3.0^44^ and ridigbio v0.3.5^45^ on June 4th, 2019. No further taxonomic name resolution was conducted for these rosid names, because all rosid species names in our tree were validated and reconciled by The Plant List and OpenTree databases in Sun et al.^41^, and we queried rosid species occurrence records with accepted rosid names. During our initial data acquisition, we extracted only data records that were georeferenced and excluded any coordinates with zero and/or integer latitude and longitude. We then used Python scripts from Folk et al.^10^ and custom R scripts to remove geographic outliers beyond three standard deviations of Euclidean distance from the geographic centroid of species occurrences and to filter missing environmental data. For more details on processing occurrence records, see Supplementary Method 1.

### Temperature data layers

We used four data sets to assess the relationship between temperature and rosid diversification rates. We first assessed the relationship of species-specific diversification rates (i.e., tip rates; see below) with contemporary temperature niche by calculating species mean temperature. For each species, we associated all assembled occurrence points with average monthly annual mean temperature data from 1970 to 2000 using WorldClim (i.e., bio1). We then used a geological historical oxygen isotope (δ^18^O) curve from the Late Cretaceous to the present (~113 Myr to present^59^), converting these values to 5-point temperature means following Folk et al.^10^, to assess the impact of global historical temperature trends on diversification rates as detailed below.

Many previous studies have invoked high diversification rate as an explanation for high biodiversity in the tropics^89–91^, although this has proved controversial (e.g., reviewed in Rabosky^92^). We tested this hypothesis by developing two further binary data sets to represent a tropical/non-tropical contrast. We used both geographic and climatic definitions to classify rosid species as occurring either inside or outside of tropical environments. For the geographic definitions, we calculated the mean latitude of occurrence records for each species and classified a species as tropical if this mean value fell between the Tropics of Cancer and Capricorn (23.43677°N and 23.43677°S), and non-tropical (i.e., temperate + polar) if it fell outside this interval. The climatic tropicality data set used the standard Köppen–Geiger climatic tropics definition as calculated by Owens et al.^43^, which defines as tropical those regions with year‐round monthly mean temperatures >18 °C. We associated species occurrences with the Owens et al.^43^ data set and classified each species based on the more frequent value (e.g., species with >50% of occurrences outside tropical sites were classified as non-tropical). We tested for the presence of phylogenetic signal in the three contemporary niche data sets using the lambda transform and Likelihood Ratio Test (Pagel’s *λ*) functions in R packages phytools v0.7-00^52^ and geiger v2.0.6.2^93^. This test assumes a phylogenetic signal definition of niche conservatism, one among several that have been proposed^53,94^.

### Diversification rate inference and statistical analysis

We first used BAMM v2.5.0^49^ to explore the diversification profiles of the rosids, and we inferred species-specific diversification rates (i.e., tip rates), diversification rate shifts, and diversification rate-through-time matrices and plots. Then we applied these diversification metrics, together with other diversification methods and the statistical tests introduced below, to test, assess, and cross-check the correlation pattern we observed.

For the initial BAMM method, we confirmed convergence of the MCMC chains and effective sample sizes >200 for both the number of shifts and log likelihoods, after discarding 10% burn-in (Supplementary Table 7). We accounted for incomplete sampling in BAMM^49^ and RPANDA^60^ based on species richness summarized from the OTT database^41^ (Supplementary Table 7).

We used tip rates as estimated in BAMM and DR^17,83^ (Supplementary Method 3) as reliable estimators to investigate speciation rate dynamics and trait-dependent (e.g., temperature-dependent) speciation as reviewed in Title & Rabosky^95^. We implemented a series of explicitly phylogenetic parametric and semi-parametric methods to test the relationship of tip rates to mean annual temperature and to the tropicality of contemporary rosid species (Supplementary Method 2). For parametric analyses, we used STRAPP^50^ as implemented in BAMMtools^96^, testing each of the 17 rosid clades recognized as orders. To cross-check the correlation pattern observed by STRAPP in BAMM, semi-parametric analyses were employed. We used tip rates estimated by DR in the es-SIM tests^51^ for the continuous mean annual temperature data set and FiSSE tests^54^ for the categorical tropicality data sets. Because it was computationally feasible to use semi-parametric methods for larger trees, we tested both the 17 rosid ordinal clades and the total tree; STRAPP was only used on the ordinal subtrees. All tests across rosid orders were controlled for family-wise error by the Hochberg^97^ method.

As an additional parametric approach to test for associations between tropicality and diversification rate, and to test for potential unobserved diversification drivers, we fit two binary state speciation and extinction (BiSSE^56^) and two hidden-state speciation and extinction (HiSSE^55^) models. These approaches estimate state-specific rates of diversification and state transition, assessing whether diversification rates are significantly different for the alternative states and whether unobserved traits influence diversification rates under a model selection framework. These analyses were executed in the R package HiSSE v1.9.6^55^ (for further implementation details, see Supplementary Method 3 and Supplementary Table 4).

In addition, we explored the spatial diversification patterns of rosids using tip rates estimated from both BAMM and DR via the R package dggridR v. 2.0.3^88^ to view global patterns (Fig. 2a, b).

### Diversification rates and historical temperature

To explore the patterns of global paleo-temperature on rosid diversification, we used RPANDA v1.4^60^ to fit a series of time- and temperature-dependent likelihood diversification models^3,60^. The latter models used the global historical temperature data set described above. Speciation and extinction dependence were modeled as all possible combinations of constant, linear, and exponential relationships, as well as pure-birth models for all three relationships. All models used are described in Supplementary Method 5.

We also used metrics from BAMM to test the relationship of diversification rate with historical global temperature found in RPANDA. We extracted rate-through-time curves in 100 time-unit slices and fit linear and exponential regression models with temperature, using AIC for model choice (Supplementary Fig. 2). Furthermore, we used diversification rate-through-time matrices and plots generated by BAMM v2.5.0^49^ to assess the relationship between geological historical temperature and net diversification rate curves for tropical and non-tropical lineages, respectively (Fig. 3). These analyses, unlike similar analyses in RPANDA (see “Results” section), do not account for temporal autocorrelation of diversification rates. However, given that RPANDA results show that the temperature–diversification relationship we observed cannot be explained by time-dependency alone, we have included regression results to present an independent method with easily interpreted correlation statistics.

We accounted for incomplete phylogenetic sampling in BAMM, STRAPP, RPANDA, and HiSSE/BiSSE with global sampling fractions based on species richness summarized from the OTT database^41^ (Supplementary Table 7). Semi-parametric DR and es-SIM methods do not parameterize incomplete sampling so these approaches were used directly (but see the similarity of tip rates across methods, Fig. 2a, b). The best model was selected by the smallest AIC value (or ∆AIC) and largest AW for HiSSE and RPANDA.

### Sensitivity tests

We assessed the sensitivity of our results to potentially uneven occurrence data accumulation between tropical and non-tropical species, the former of which are expected to be under-sampled^98^, by a series of random taxon dropping experiments (Supplementary Method 5). For each ordinal subtree, 10%, 30%, or 50% of non-tropical species were dropped while all tropical species were retained. We then re-ran STRAPP analyses following the methods described above and assessed the impact on estimated tip rates and downstream analyses.

### Reporting summary

Further information on research design is available in the Nature Research Reporting Summary linked to this article.

## Supplementary information

Supplementary Information

Peer Review File

Reporting Summary

## Data Availability

Phylogenetic trees, species distribution data, WorldClim temperature data, and BAMM diversification rate-through-time matrices are available at https://github.com/Cactusolo/rosid_NCOMMS-19-37964-T (10.5281/zenodo.3843441)^99^; the source data underlying Fig. 2, pie-chart of Fig. 3, and Supplementary Fig. 3 are provided as a Source Data file; all other relevant intermediate data are available from the authors upon request.

## Source data

Source Data

## Code availability

All code used in this study is available at https://github.com/Cactusolo/rosid_NCOMMS-19-37964-T (10.5281/zenodo.3843441)^99^.
