# Supplementary material for: Recent accelerated diversification in rosids occurred outside the tropics
Source: Nat Commun. 2020 Jul 3;11:3333. doi: 10.1038/s41467-020-17116-5 (PMC7335165; doi:10.1038/s41467-020-17116-5)
Supplement: Supplementary file 1 — Supplementary Information [file 41467_2020_17116_MOESM1_ESM.pdf]

Sun *et al.* (2020) Recent accelerated diversification in rosids occurred outside the tropics

Supplementary Information:

Supplementary Method: 1-5

Supplementary Table: 1-7

Supplementary Figure: 1-3

Supplementary References

Supplementary Methods

Supplementary Method 1: Species distribution data assembly and temperature data layers

1) ***Assembling and cleaning coordinates***

We queried all 19,740 name validated rosid species sampled in our tree from iDigBio and GBIF using R packages rgbif v1.3.0[1,2] and ridigbio v0.3.5[3] on June 4th, 2019. During our initial query and data assembly steps, we only extracted geo-referenced data and excluded any coordinates with zero latitude and longitude. For each individual species, we used a custom R script to calculate the geographic centroid and the Euclidean distance from each species occurrence to this centroid, removing any geographic outliers beyond three standard deviations distant. We did not impose a minimum occurrence record limit in order to maximize species representation.

2) ***BioClim mean annual temperature layer***

We downloaded a global mean annual temperature dataset (bio1 from BioClim), a dataset representing temperatures from 1970 to 2000, averaged monthly at 30-second spatial resolution (~1 km^2) from the WorldClim website. We used a Python script from Folk et al.[4] to extract temperature from each locality record, excluding any points with missing data. We then calculated a species mean temperature, which was used in downstream phylogenetic comparative analyses.

3) ***Score the binary geographic and climatic tropicality datasets***

Geographic tropicality dataset

For each species, we calculated the mean latitude of geo-referenced locality records and classified the species as tropical if this value fell between the Tropics of Cancer and Capricorn (23.43677°N and 23.43677°S), and non-tropical (i.e. temperate + polar) if it fell outside this interval.

Climatic tropicality dataset

The climatic tropicality dataset used the Köppen-Geiger climatic tropics definition as previously calculated by Owens et al.[5] (that is, defining as tropical those regions with year-round monthly mean temperatures of > 18 °C). As with mean annual temperature, we used Python script[4] to associate locality data with global tropicality classifications. We then calculated the mode for each species (i.e., the most prevalent tropicality

classification; thus species with >50% occurrences outside tropical sites were classified as non-tropical).

Supplementary Method 2: Diversification analyses implemented by BAMM & DR

We conducted diversification analyses across rosid lineages using BAMM v2.5.0[6]. BAMM is able to account for non-random and incomplete sampling, allowing the user to assign tips to clades (e.g., orders and families in our case) and indicate the total proportion of the clade sampled within the phylogeny. Analyzing the entire 5-locus, 19,740-taxon rosid phylogeny would require excessive computational resources for BAMM to reach MCMC convergence. To deal with potential convergence issues, we conducted independent analyses of 17 subtrees representing recognized orders recovered in the 5-locus tree.

In most cases, four independent MCMC chains of 20,000,000 generations were run and those running priors were estimated using the R package BAMMtools v2.1.6[7] with the "setBAMMpriors" function. For larger rosid subclades, the number of generations in MCMC chains, the number of expected shifts (expectedNumberOfShifts = 10, if tips > 5000) and rate priors were manually adjusted to ensure MCMC convergence (see Supplementary Table 1). Parameter effective sample sizes (>200 for both the number of shifts and log likelihoods) and convergence among chains were assessed in the R package coda v0.19-1[8]. After removing 10% of the trees as burn-in, we explored the BAMM output using BAMMtools.

As an alternative to BAMM for investigating speciation rate dynamics and rate-trait correlation patterns, we employed the DR statistic[9], one of the most widely used semiparametric approaches to diversification estimation[9,10,11,12]. The DR statistic quantifies the "splitting rate" from each extant species to the tree root as a "likelihood-free" estimate of diversification rate. Methods followed those described in Jetz et al.[9] and Harvey et al.[10]. Due to its more rapid computation, the DR statistic enabled us to estimated rates from the whole rosid tree and as well as 17 rosid ordinal clades for es-SIM tests[13] on continuous mean annual temperature data and for FiSSE tests[14] on categorical tropicality data. Since it was impossible for BAMM to achieve convergence in the global 20k-tip rosid tree, STRAPP tests were performed only for subclades. To further explore tips rates in global pattern, we conducted the spatial diversification patterns analyses using R package dggridR v. 2.0.3[15].

Lastly to account for recent criticism relating to the statistical methods for lineage-

specific diversification models and diversification rate estimation[16,17,18,19], which advocated for problematic behavior of BAMM under certain conditions, we compared BAMM results with other diversification analyses implemented with RPANDA and the DR statistic, and used the most updated version of BAMM and BAMMtools[20], considering the author's response notes[21,22].

Supplementary Method 3: Hidden-state speciation and extinction (HiSSE) models

We implemented binary state speciation and extinction (BiSSE[23]) and hidden state speciation and extinction (HiSSE[24]) models, executed in R packages HiSSE v1.9.6[24].

We used the following labeling conventions: non-tropical state = 0; tropical state = 1, hidden character states = A (absent), B (present). Hence the full states of the model are 0A, 1A, 0B, 1B. A standard BiSSE model does not include a hidden state; this was implemented by disallowing transitions to the hidden states 0B or 1B. The HiSSE function "TransMatMaker" takes "turnover.anc" and "eps.anc" as arguments. These arguments control the number of rate classes for the HiSSE model. The order of classes in these arguments corresponds to 0A, 1A, 0B, 1B; distinct states are given by successive non-zero integers while 0 removes a parameter.

We compared the fit of four different models: (one BiSSE-like model, one BiSSE-like null model, one HiSSE full model, and one HiSSE 2-state null model): (1A) a full BiSSE model (here, "BiSSE_full") with two observed states and no hidden state, and constrained equal extinction ($\mu0=\mu1$) and transitions (q01=q10) associated with each state; (2A) a BiSSE-like null model (here "BiSSE_null") with two observed states and no hidden states, constrained speciation and extinction rates ($\lambda0 = \lambda1$, $\mu0 = \mu1$). We then ran two HiSSE models: (1B) "HiSSE_full" model with two hidden states (A, B) contained within each observed state (i.e., states 0A, 1A, 0B, 1B), turnover rate vary freely for 4 states (no constraint); (2B) "HiSSE_cid2_null" model, i.e., the 2-state character independent diversification model (also called CID-2); we use this as our null model by forcing the two observed states (non-tropical or tropical) to have the same net turnover rates, while permitting the hidden states to vary freely. All four models were implemented in R package HiSSE V.1.9.6[24], and the rest parameters were the default in the HiSSE package. Comparisons were conducted for both tropicality traits on the large-scale rosid phylogeny. The best model is evaluated based on ΔAIC and Akaike weights (AW)[25]. All models are described in Supplementary Table 4.

Supplementary Method 4: Sensitivity test of uneven occurrence data between tropical and non-tropical species

Bias in patterns of incomplete sampling, especially for tropical species, are well known in existing occurrence record repositories. Limited work has been done to date assessing the impact of these biases on diversification or on questions specifically relevant to tropicality such as those we pose here. We assessed the impact of this potential bias with two approaches. First, we plot all the sampled rosid occurrence data on a equal-area global grid with cells approximately 322 km (ca. 200 miles) across and counted number of species within those cells using R package dggridR v. 2.0.3[15] to display overall global richness patterns for rosids based on current sampling (Supplementary Fig. 1). Our overall expectation (see Kreft & Jetz[26], who considered all vascular plants), is that, rosid diversity in non-tropical areas should be lower than in equatorial regions. The map (Supplementary Fig. 1) approximately demonstrates expected richness patterns but with over-representation of species in North America and Western Europe.

Given evidence for over-representation in some non-tropical areas, we implemented a sensitivity analysis, simulating datasets where that bias is reduced or potentially even inverted. We achieved this by randomly dropping 10%, 30%, or 50% of non-tropical species from the tips of each ordinal subtree, while retaining all tropical species. In the case of the 50% drop of non-tropical species, tropical species outnumber temperate ones for most clades, simulating over-representation of tropical species. We used "getTipRates" function in BAMMtools[7] to get tip rates form each ordinal subtree at both before and after each of dropping-taxon treatment; then reran STRAPP procedure[27] as implemented in BAMMtools[7] as also mentioned in the main text; and lastly we assessed the impact on estimated tip rates and downstream phylogenetic regressions. The results of this analyses are summarized in Supplementary Table 7. We find that (1) diversification rate results are very robust to removal of non-tropical species, suggesting that biases in number of species included from different regions do not impact the main findings here; and that (2) overall reduction in species sampling also does not have a dramatic impact on assessment of rates. While inclusion of more species is clearly critical for producing the highest quality phylogenetic framework for downstream analysis[12], the tip diversification rate assessment is robust to species sampling issues. This result makes intuitive sense because (1) we used phylogenetic methods that derive diversification estimates from branch length information as well as species count data, and (2) most methods implemented have clade-specific corrections

for missing species, reducing the impact of biased sampling effort.

Supplementary Method 5: Diversification analyses implemented by RPANDA

We used RPANDA v1.4[28] to fit a series of time- and temperature-dependent likelihood diversification birth-death models[28,29] for the time-calibrated 5-locus rosid tree whose details can be found in Sun et al.[30]. In total, we tested 18 likelihood-based diversification models (also see Sun et al.[12]):

Nine time-dependent models

1) Pure birth model, no extinction rate (mu, $\mu = 0$), and constant speciation rate (lambda, λ; hereafter bcst.d0.t)

   # *t* is time

   # *y* is a vector of initial values feeding to the functions of λ and μ

   f.lamb = function(t, y){y[1]}

   f.mu = function(t, y){0}

2) Birth-death model with constant speciation and extinction (here as bcst.dcst.t)

   f.lamb = function(t, y){y[1]}

   f.mu = function(t, y){y[1]}

3) Pure birth model with exponential variation in speciation rate (here as bvar.d0.t)

   *f.lamb = function(t, y){y[1]*exp(y[2]*t)}*

   f.mu = function(t, y){0}

4) Pure birth model with linear variation in speciation rate (here as bvar.l.d0.t)

   *f.lamb = function(t, y){y[1] + y[2]*t}*

   f.mu = function(t, y){0}

5) Birth-death model with exponential variation in speciation rate and constant extinction (here as bvar.dcst.t)

   *f.lamb = function(t, y){y[1]*exp(y[2]*t)}*

   f.mu = function(t, y){y[1]}

6) Birth-death model with linear variation in speciation rate and constant extinction (here as bvar.l.dcst.t)

   *f.lamb = function(t, y){y[1] + y[2]*t}*

   f.mu = function(t, y){y[1]}

7) Birth-death model with a constant speciation rate and exponential variation in extinction (here as bcst.dvar.t)

f.lamb = function(t, y){y[1]}

*f.mu = function(t,y){y[1]*exp(y[2]*t)}*

8) Birth-death model with a constant speciation rate and linear variation in extinction (here as bcst.dvar.l.t)

f.lamb = function(t, y){y[1]}

*f.mu = function(t,y){y[1] + y[2]*t}*

9) Birth-death model with exponential variation in speciation and extinction (here as bvar.dvar.t)

*f.lamb = function(t, y){y[1]*exp(y[2]*t)}*

*f.mu = function(t,y){y[1]*exp(y[2]*t)}*

Nine temperature-dependent models

We also tested nine environmental-dependent diversification models inferred from oxygen isotopes ($\delta^{18}O$) covering major changes of global temperature since the Late Cretaceous (~ 113 Myr to present)[31,32].

10) No extinction rate (mu, $\mu = 0$), and constant speciation rate with temperature (x) (lambda, λ; hereafter bcst.d0.x)

t is time

x is temperature

y is a vector of initial values feeding to the functions of λ and μ

*f.lamb = function(t,x,y){y[1]*x}*

f.mu = function(t,x,y){0}

11) Constant speciation and extinction with temperature (x) (here as bcst.dcst.x)

*f.lamb = function(t,x,y){y[1]*x}*

*f.mu = function(t,x,y){y[1]*x}*

12) Exponential variation in speciation rate with temperature (x) (here as bvar.d0.x)

*f.lamb = function(t,x,y){y[1]*exp(y[2]*x)}*

f.mu = function(t,x,y){0}

13) Linear variation in speciation rate with temperature (x) (here as bvar.l.d0.x)

*f.lamb = function(t,x,y){y[1] + y[2]*x}*

f.mu = function(t,x,y){0}

14) Exponential variation in speciation rate with temperature (*x*) and constant extinction with temperature (*x*) (here as bvar.dcst.x)

    *f.lamb = function(t,x,y){y[1]*exp(y[2]*x)}*

    *f.mu = function(t,x,y){y[1]*x}*

15) Linear variation in speciation rate with temperature (*x*) and constant extinction with temperature (*x*) (here as bvar.l.dcst.x)

    *f.lamb = function(t,x,y){y[1] + y[2]*x}*

    f.mu = function(t, y){y[1]}

16) Constant speciation rate with temperature (*x*) and exponential variation in extinction with temperature (*x*) (here as bcst.dvar.x)

    *f.lamb = function(t,x,y){y[1]*x}*

    *f.mu = function(t,x,y){y[1]*exp(y[2]*x)}*

17) Constant speciation rate with temperature (*x*) and linear variation in extinction with temperature (*x*) (here as bcst.dvar.l.x)

    *f.lamb = function(t,x,y){y[1]*x}*

    *f.mu = function(t,x,y){y[1] + y[2]*x}*

18) Exponential variation both in speciation and extinction rates with temperature (*x*) (here as bvar.dvar.x)

    *f.lamb = function(t,x,y){y[1]*exp(y[2]*x)}*

    *f.mu = function(t,x,y){y[1]*exp(y[2]*x)}*

The first nine birth-death models were calculated using the fuction “fit_bd”[28], and the rest models were calculated with the function “fit_env”. We compared the likelihood supports, the Akaike Information Criterion (AIC)[33] and AW[25] for the 18 different models[29]; the model with the smallest AIC value and largest AW was chosen as the best diversification model.

Supplementary Tables

Supplementary Table 1. Summary table for 17 rosid orders, giving species richness, occurrence records, and summaries of temperature data layers.

Order	Richness	Occ. recs.	Ann. Tm. recs	Köpp. recs.	Köp		Geo	
					Non-trop.%	Trop. %	Non-trop.%	Trop.%
Brassicales	5,940	215,259	63,136	122,654	91.50%	8.50%	82.03%	17.97%
Celastrales	1,499	32,479	9,715	18,538	67.33%	32.67%	52.12%	47.88%
Crossosomatales	82	4,709	1,589	2,891	95.83%	4.17%	75.00%	25.00%
Cucurbitales	3,094	57,686	18,314	33,428	50.21%	49.79%	23.08%	76.92%
Fabales	24,191	788,889	221,934	428,365	66.95%	33.05%	54.76%	45.24%
Fagales	2,263	121,488	33,110	64,770	86.19%	13.81%	75.73%	24.27%
Geraniales	962	36,961	11,419	21,042	99.66%	0.34%	87.29%	12.71%
Huerteales	30	767	186	392	71.43%	28.57%	71.43%	28.57%
Malpighiales	21,316	449,108	129,486	251,102	47.24%	52.76%	32.79%	67.21%
Malvales	7,771	154,303	44,164	83,855	58.20%	41.80%	44.40%	55.60%
Myrtales	15,529	213,248	58,629	109,399	58.38%	41.62%	41.13%	58.87%
Oxalidales	2,207	22,340	6,734	11,715	78.77%	21.23%	51.35%	48.65%
Picramniales	57	1,846	513	886	20.00%	80.00%	0	100.00%
Rosales	20,620	334,620	94,921	189,341	86.72%	13.28%	76.36%	23.64%
Sapindales	7,421	224,749	58,500	111,166	49.30%	50.70%	36.28%	63.72%
Zygophyllales	340	11,147	3,112	5,909	78.33%	21.67%	58.33%	41.67%
Vitales	1,155	21,500	6,581	12,818	47.27%	52.73%	38.18%	61.82%
Total summary	114,477	2,691,099	762,043	1,468,271	65.07%	34.93%	51.45%	48.55%

Notes:

For the different temperature datasets, the numbers of unique records are dependent on each of the data layer resolution.

Richness: Species richness as estimated from OpenTree (cf. Ref.[30])

Ann. Tm. recs: records of mean annual temperature (bio1 from WorldClim) for each order

Occ. recs.: occurrence records obtained from iDigBio and GBIF.

Köpp. recs.: Records successfully associated with Köppen-Geiger climatic data. Geographic tropicality is not given because this was successfully associated with all records.

Non-trop.% and Trop.%: percentage of non-tropical and tropical rosid species based on climatic (Köp.) and geographic (Geo.) binary tropicality definitions, respectively (see also Fig. 3).

Supplementary Table 2. Lambda transform and likelihood ratio tests (one-tailed test) for the presence of phylogenetic niche conservatism in the three contemporary temperature niche datasets.

Traits	Pagel's λ test of phylogenetic signal			
	Null Hypothesis	*Alternative Hypothesis*		
	**$\log L_{non\text{-}tropical}$**	**$\log L_{tropical}$**	***p*-value**	***λ***
Mean annual temperature	-95722.06	-85674.52	**0**	0.862086
Köppen tropicality	-7713.990061	-7156.118049	**1.25E-244**	0.9562965
Geographic tropicality	-7386.139644	-6686.356871	**2.61E-306**	0.9381459

Supplementary Table 3. Summary table of parametric testing analyses for correlation between speciation rate and temperature and between tropicality traits using STRAPP.

Order	MAT		Köp		Geo	
	p-value	pv.adjust	*p*-value	pv.adjust	*p*-value	pv.adjust
Brassicales	0.027	0.432	0.127	0.999	0.482	0.999
Celastrales	0.723	0.999	0.674	0.999	0.724	0.999
Crossosomatales	0.975	0.999	0.985	0.999	0.978	0.999
Cucurbitales	0.679	0.999	0.229	0.999	0.392	0.999
Fabales	0.005	0.085	0	**0**	0.01	0.160
Fagales	0.239	0.999	0.311	0.999	0.433	0.999
Geraniales	0.893	0.999	0.958	0.999	0.918	0.999
Huerteales	0.997	0.999	0.999	0.999	0.996	0.999
Malpighiales	0.051	0.765	0.08	0.999	0.156	0.999
Malvales	0.643	0.999	0.593	0.999	0.856	0.999
Myrtales	0.359	0.999	0.559	0.999	0.63	0.999
Oxalidales	0.995	0.999	0.998	0.999	0.999	0.999
Picramniales	0.994	0.999	0.995	0.999	NA	NA
Rosales	0.376	0.999	0.413	0.999	0.589	0.999
Sapindales	0.233	0.999	0.249	0.999	0.321	0.999
Vitales	0.969	0.999	0.979	0.999	0.976	0.999
Zygophyllales	0.999	0.999	0.999	0.999	0.999	0.999

Notes:

MAT: continuous mean annual temperature

Two tropicality traits: Köppen-Geiger climatic(Köp) and geographic (Geo)

p-value is for correlation between speciation rate and MAT, Köp and Geo datasets, respectively; the significant *p*-values are in bold;

pv.adjust refers to adjusting the corresponding *p*-value for family-wise error using the method from ref.[34]

For Picramniales, all species were scored as tropical, hence the binary-trait test in STRAPP is not applicable and this order is marked "NA"

Table S4. HiSSE modules fitted for investigating correlation of rosid diversification and climatic and geographic tropicality temperature traits.

Traits	Model	Hidden States	net turnover rate	extinction fraction	transition rates	Loglik	AIC	AW	ΔAIC	np
Köp	BiSSE_full	FALSE	vary	equal	equal	-60254.91553	120517.8311	0	5219.6499	4
	BiSSE_null	FALSE	equal	equal	equal	-60255.67164	120517.3433	0	5219.1621	3
	**HiSSE_full**	**TRUE**	**vary**	**equal**	**equal**	**-57643.0906**	**115298.1812**	**1**	**0**	**6**
	HiSSE_cid2_null	TRUE	equal	equal	equal	-58642.94677	117293.8935	0	1995.7123	4
Geo	BiSSE_full	FALSE	vary	equal	equal	-61692.79466	123393.5893	0	5094.4474	4
	BiSSE_null	FALSE	equal	equal	equal	-61886.74018	123779.4804	0	5480.3385	3
	**HiSSE_full**	**TRUE**	**vary**	**equal**	**equal**	**-59143.57097**	**118299.1419**	**1**	**0**	**6**
	HiSSE_cid2_null	TRUE	equal	equal	equal	-59798.55173	119605.1035	2.59e-284	1305.9616	4

Notes:

Climatic tropicality is Köppen-Geiger climatic tropics definition, see ref.[5]; the best model based on ΔAIC and Akaike weights (AW) denoted in bold; np is the number of parameters.

Supplementary Table 5. Summary table for sensitivity test of uneven occurrence data between tropical and non-tropical species.

Order	Global tip rate	Treatment	Geo				Köp				Mean annual temperature		
			Tip rate treatment	Trop. rate treatment	Non-trop. rate treatment	P-value	Tip rate treatment	Trop. rate treatment	Non-trop. rate treatment	P-value	Tip rate treatment	Estimated rate treatment	P-value
	0.72641274	10% drop	0.732314662	0.650842538	0.642640425	0.5	0.727359636	0.168256755	0.646386042	0.108	0.724216301	-0.386760116	0.022
**Brassicales**	0.72641274	30% drop	0.724456443	0.650842538	0.642349984	0.507	0.718995948	0.168256755	0.650891457	0.111	0.707690399	-0.412434693	0.021
	0.72641274	50% drop	0.737270109	0.650842538	0.646380123	0.417	0.704248942	0.168256755	0.651015942	0.118	0.696403331	-0.422874126	0.035
	0.608173462	10% drop	0.611404025	0.483998274	0.876632652	0.751	0.605998855	0.483335806	0.876691195	0.675	0.607320929	-0.092800598	0.683
**Celastrales**	0.608173462	30% drop	0.612951899	0.483998274	0.876632652	0.729	0.605741179	0.483335806	0.87686046	0.737	0.611128206	-0.113006555	0.704
	0.608173462	50% drop	0.612017916	0.483998274	0.876834127	0.732	0.598439862	0.483335806	0.876722298	0.704	0.579176856	-0.081432842	0.741
	0.222794402	10% drop	0.224233923	0.237267586	0.241803424	0.961	0.22236251	0.241803424	0.241803424	0.978	0.222401849	-0.060476582	0.983
**Crossosomatales**	0.222794402	30% drop	0.223042144	0.237267586	0.241803424	0.957	0.2249342	0.241803424	0.24002512	0.986	0.223126601	-0.026265415	0.972
	0.222794402	50% drop	0.221430586	0.237267586	0.241803424	0.944	0.226414803	0.241803424	0.241803424	1	0.214976239	-0.046933886	0.97
	0.416701188	10% drop	0.409486767	0.311867744	0.314155437	0.44	0.402768635	0.311340984	0.315850664	0.237	0.403826539	-0.057485089	0.626
**Cucurbitales**	0.416701188	30% drop	0.409430832	0.311867744	0.340292206	0.328	0.38884209	0.311340984	0.314114973	0.326	0.392884082	-0.033635149	0.751
	0.416701188	50% drop	0.399434678	0.311867744	0.315356616	0.531	0.386238938	0.311340984	0.313702179	0.367	0.380795951	-0.016663618	0.778
	1.080860769	10% drop	1.015500458	0.625967934	0.93202733	0.007	0.998728059	0.621794559	0.826571163	0.003	0.971364664	-0.362551051	0.003
**Fabales**	1.080860769	30% drop	0.96893731	0.625967934	0.923181949	0.009	0.981515403	0.621794559	0.85463543	0.001	0.9413272	-0.355169456	0.008
	1.080860769	50% drop	0.936971992	0.625967934	0.932614871	0.007	0.912550295	0.621794559	0.818092033	0.007	0.885509346	-0.321325059	0.016
	1.507840887	10% drop	1.49245331	1.131389349	1.681770857	0.462	1.492212999	0.586731873	1.682108694	0.319	1.481802406	-0.236873972	0.27
**Fagales**	1.507840887	30% drop	1.465473273	1.131389349	1.68090723	0.468	1.447034207	0.586731873	1.615534664	0.386	1.472792489	-0.234200923	0.294
	1.507840887	50% drop	1.45265483	1.131389349	1.682624856	0.484	1.427948314	0.586731873	1.681998731	0.32	1.452724557	-0.318215098	0.184
	0.644325704	10% drop	0.644336094	0.63747039	0.638570184	0.944	0.644446857	0.63747039	0.638570184	0.948	0.644403559	0.03903089	0.887
**Geraniales**	0.644325704	30% drop	0.644423452	0.63747039	0.638570184	0.935	0.644074264	0.63747039	0.638570184	0.94	0.644109562	0.042366366	0.905
	0.644325704	50% drop	0.644291933	0.63747039	0.638570184	0.916	0.645263922	0.63747039	0.641135541	0.947	0.645014519	0.031682808	0.922
	0.046143577	10% drop	0.046143577	0.055205683	0.058821719	0.999	0.046143577	0.055205683	0.058821719	0.998	0.046143577	-0.006149065	0.995
**Huerteales**	0.046143577	30% drop	0.046259997	0.055205683	0.058821719	0.998	0.046259997	0.055205683	0.058821719	0.997	0.045747171	-0.002990482	0.992
	0.046143577	50% drop	0.046019479	0.055205683	0.058821719	0.997	0.046259997	0.055205683	0.058821719	0.998	0.046019479	-0.013306033	0.997
	0.718235017	10% drop	0.709723388	0.600144995	0.689307945	0.156	0.706412766	0.573621457	0.686878969	0.09	0.69383343	-0.223696022	0.05
**Malpighiales**	0.718235017	30% drop	0.701068412	0.600144995	0.689332659	0.146	0.693734424	0.573621457	0.647532577	0.102	0.675850008	-0.216259981	0.046
	0.718235017	50% drop	0.671398383	0.600144995	0.687009885	0.194	0.655479131	0.573621457	0.631997606	0.115	0.664815004	-0.208079975	0.026
	1.212176034	10% drop	1.206669644	1.296044995	0.647365874	0.829	1.199929871	0.890325368	1.278719633	0.62	1.194592936	-0.113333317	0.636
**Malvales**	1.212176034	30% drop	1.220762319	1.296044995	0.844215262	0.838	1.190207771	0.890325368	1.278505089	0.594	1.178945306	-0.098069942	0.626
	1.212176034	50% drop	1.226685231	1.296044995	1.198776448	0.836	1.176545278	0.890325368	1.201690365	0.645	1.162873011	-0.119402549	0.6
	1.665370759	10% drop	1.657783562	1.612235835	1.610601207	0.549	1.654578388	1.600527352	1.613507835	0.524	1.647833219	-0.296887556	0.43
**Myrtales**	1.665370759	30% drop	1.625777035	1.612235835	1.607675907	0.673	1.605382704	1.600527352	1.612967214	0.555	1.630244389	-0.290756725	0.371
	1.665370759	50% drop	1.589683611	1.612235835	1.607675907	0.688	1.590556935	1.600527352	1.613775472	0.472	1.590072831	-0.281602909	0.357
	0.859858516	10% drop	0.859880464	0.861276872	0.861361485	0.995	0.859959139	0.861639647	0.861361485	0.996	0.859710606	0.004122843	0.996
**Oxalidales**	0.859858516	30% drop	0.86011294	0.861276872	0.861266884	0.997	0.859989752	0.861639647	0.861361485	0.996	0.860052546	0.003210864	0.996
	0.859858516	50% drop	0.860190337	0.861276872	0.861498014	0.998	0.860039938	0.861639647	0.861426653	0.996	0.860057883	0.003449307	0.998
**Picramniales**	0.268442543	10% drop	NA	NA	NA	NA	0.268442543	0.295160271	0.31286426	0.998	0.268442543	-0.00956969	0.998

	0.268442543	30% drop	NA	NA	NA	NA	0.268442543	0.295160271	0.31286426	0.997	0.268442543	-0.001440622	0.999
	0.268442543	50% drop	NA	NA	NA	NA	0.268442543	0.295160271	0.31286426	0.999	0.268442543	-0.002390239	0.995
**Rosales**	2.440185793	10% drop	2.441830016	2.527825875	2.202351755	0.628	2.435742614	0.978685148	2.652744986	0.425	2.443715183	-0.241345674	0.407
	2.440185793	30% drop	2.407056391	2.527825875	2.184393415	0.605	2.399303115	0.978685148	2.504520755	0.446	2.408773639	-0.268033214	0.373
	2.440185793	50% drop	2.401613833	2.527825875	2.202245521	0.641	2.381190308	0.978685148	2.640462678	0.436	2.314862167	-0.240839037	0.465
**Sapindales**	0.483309111	10% drop	0.479879872	0.406638351	0.659600511	0.337	0.47686237	0.402615137	0.538623754	0.272	0.473708375	-0.183462198	0.271
	0.483309111	30% drop	0.475190259	0.406638351	0.650095736	0.329	0.469573717	0.402615137	0.548543371	0.255	0.464285433	-0.181490407	0.241
	0.483309111	50% drop	0.465985924	0.406638351	0.658315157	0.303	0.456238692	0.402615137	0.476834805	0.376	0.460413965	-0.182197669	0.203
**Zygophyllales**	0.199413481	10% drop	0.199403457	0.206623017	0.206623017	1	0.199403178	0.206623017	0.206623017	1	0.199402889	-8.16E-05	1
	0.199413481	30% drop	0.199401617	0.206623017	0.206623017	1	0.199400124	0.206623017	0.206623017	1	0.199399276	0.000991593	1
	0.199413481	50% drop	0.199418784	0.206623017	0.206623017	1	0.199394941	0.206623017	0.206623017	1	0.199418784	0.000531069	1
**Vitales**	1.002648186	10% drop	0.96378635	0.806720548	0.833263177	0.993	0.96542284	0.806720548	0.817460629	0.978	0.980087627	-0.304400001	0.984
	1.002648186	30% drop	0.944648358	0.806720548	0.8571574	0.98	0.924748739	0.806720548	0.813036444	0.986	0.964588231	-0.310418161	0.981
	1.002648186	50% drop	0.883721763	0.806720548	0.817460629	0.991	0.939155599	0.806720548	0.813036444	0.981	0.906048859	-0.235915225	0.984

Notes:

We conducted a series of 10%, 30%, or 50% of non-tropical random taxon dropping treatments for each ordinal subtree; then we reran STRAPP analyses to estimate tips rate for each treatment and corresponding correlation between its rates and temperature traits (mean annual temperature and two binary tropicality datasets). We only reported the two-tailed *p*-values from the STRAPP analyses here (see below), and we didn't do any correction for multiple comparison.

Global tip rate refers to the average of tip rates calculated for the whole ordinal subtree before taxon-dropping

Tip rate treatment refers to averaged tip rates after the 10%, 30%, or 50% treatments as indicated in the table.

Trop. rate treatment and Non-trop. rate treatment refer to tip rates estimated by STRAPP for each of the binary states (Trop. = tropical; Non-trop. = non-tropical) after each taxon-dropping treatment

Estimated rate treatment means tip rate estimated by STRAPP for the continuous mean annual temperature data after each taxon-dropping treatment

p-value is reported from two-tailed tests of U statistic (binary tropicality datasets) and spearman test (mean annual temperature), respectively.

For the binary data, some small clades were monomorphic, preventing tropicality comparisons, hence these are marked “NA”.

Supplementary Table 6. Best models selected for 17 rosid orders from time- and temperature-dependent likelihood diversification models in RPANDA.

Order	Best-model	λ-μ	AICc	AW
Brassicales	bcst.dcst.x	0.021	8863.469	1
Celastrales	bcst.dcst.x	0.016	1501.449	1
Crossosomatales	bcst.dcst.x	0.002	154.603	1
Cucurbitales	bcst.dcst.x	0.023	4429.737	1
Fabales	bcst.dcst.x	0.017	27314.173	1
Fagales	bcst.dcst.x	0.007	2276.808	1
Geraniales	bcst.dcst.x	0.011	1480.488	1
Huerteales	bcst.dcst.x	0.016	44.174	0.768
Malpighiales	bcst.dcst.x	0.015	20644.211	1
Malvales	bcst.dcst.x	0.017	6741.754	1
Myrtales	bcst.dcst.x	0.016	6990.441	1
Oxalidales	bcst.dcst.x	0.011	1134.595	1
Picramniales	bcst.dcst.x	-0.007	33.813	0.812
Rosales	bcst.dcst.x	0.011	8323.199	1
Sapindales	bcst.dcst.x	0.023	7352.161	1
Zygophyllales	bcst.dcst.x	0.008	463.538	1
Vitales	bcst.dcst.x	0.009	694.697	1

Notes:

The best models with the smallest AICc and largest Akaike weight (AW) values among 18 time- and temperature-dependent likelihood diversification models (cf. Supplementary Method 5). "bcst.dcst.x" means temperature (*x*) dependent birth-death model with constant speciation and extinction rates; the first nine models are time-dependent birth-death models, and the rest are temperature-dependent models (see Supplementary Method 5 for details).

AICc: the corrected Akaike Information Criterion (AICc) for the fitted model.

Net diversification rate (λ-μ) is calculated by the speciation rate (λ) minus the absolute value of extinction rate (μ) parameters estimated by the fitted model at present, respectively; unit is Myr^{-1}; note that negative value of λ and μ estimated by RPANDA arise from the implementation of likelihood optimization and should be interpreted as their absolute values (see author's discussion at https://github.com/hmorlon/PANDA/issues/11).

Supplementary Table 7. Summary table of BAMM MCMC chain length, convergence evaluation and sampling fraction of each rosid order.

Order	MCMC Generation	Sampling fraction	Effective Size	
			N_shifts	logLik
Brassicales	208,316,000	0.308	1359.32	2430.96
Celastrales	22,000,000	0.187	588.06	490.94
Cucurbitales	20,000,000	0.278	781.13	494.86
Fabales	134,198,000	0.235	600.01	230.90
Fagales	20,000,000	0.237	851.79	399.77
Malpighiales	111,508,000	0.18	469.09	266.18
Malvales	80,000,000	0.173	395.97	241.41
Myrtales	80,000,000	0.088	782.36	995.11
Oxalidales	22,000,000	0.087	784.08	2226.57
Rosales	200,000,000	0.086	2946.91	815.99
Sapindales	82,000,000	0.193	2015.80	1439.91
Crossosomatales	2,000,000	0.293	552.70	595.89
Geraniales	2,000,000	0.315	520.72	251.23
Huerteales	2,000,000	0.233	901.00	901.00
Picramniales	2,000,000	0.088	560.40	815.48
Vitaceae	2,000,000	0.103	272.24	378.22
Zygophyllales	2,000,000	0.206	901.00	770.95

Notes:

Sampling fractions were calculated based on the number of species sampled in each major clade recognized as an order (ref.[35]), divided by the total species richness of each clade as summarized using OpenTree (See ref.[30]).

N_shifts and logLik are two parameters (the number of shifts and log likelihoods) used to assess effective sample sizes (>200) and convergence among chains (see BAMM documentation).

Supplementary Figure

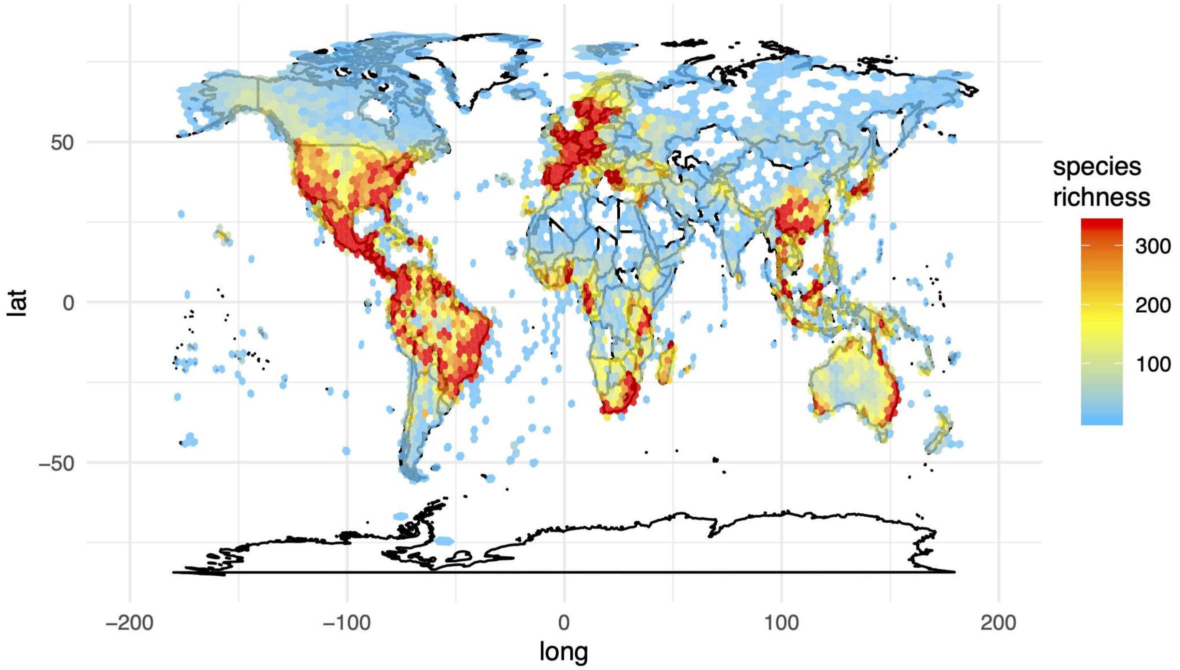

Supplementary Fig. 1. Global species richness distribution pattern for tropical and non-tropical rosid species, calculated from our occurrence record set.

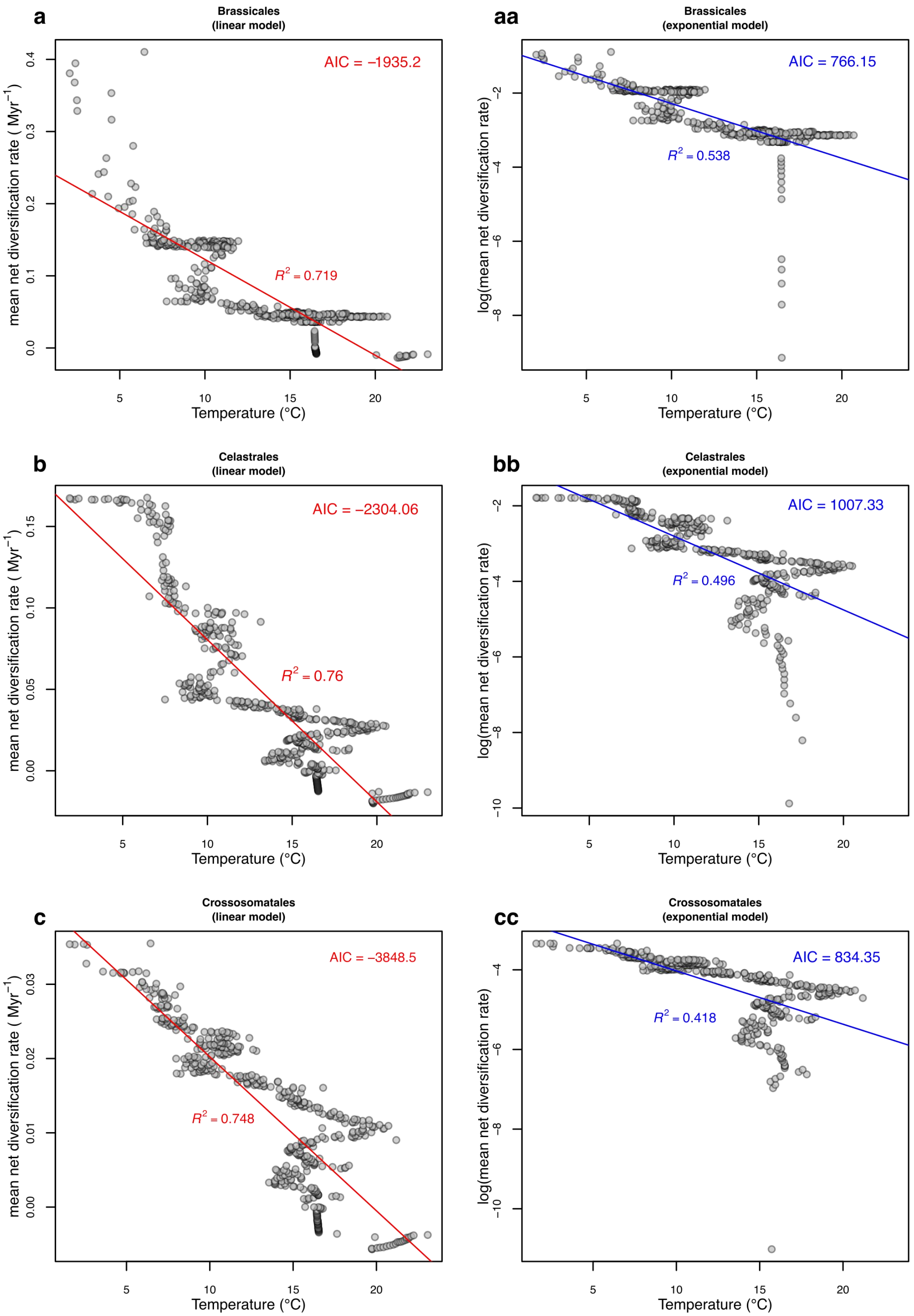
a
Brassicales
(linear model)
AIC = −1935.2
R^2 = 0.719
mean net diversification rate (Myr^{-1})
Temperature (°C)
aa
Brassicales
(exponential model)
AIC = 766.15
R^2 = 0.538
log(mean net diversification rate)
Temperature (°C)
b
Celastrales
(linear model)
AIC = −2304.06
R^2 = 0.76
mean net diversification rate (Myr^{-1})
Temperature (°C)
bb
Celastrales
(exponential model)
AIC = 1007.33
R^2 = 0.496
log(mean net diversification rate)
Temperature (°C)
c
Crossosomatales
(linear model)
AIC = −3848.5
R^2 = 0.748
mean net diversification rate (Myr^{-1})
Temperature (°C)
cc
Crossosomatales
(exponential model)
AIC = 834.35
R^2 = 0.418
log(mean net diversification rate)
Temperature (°C)

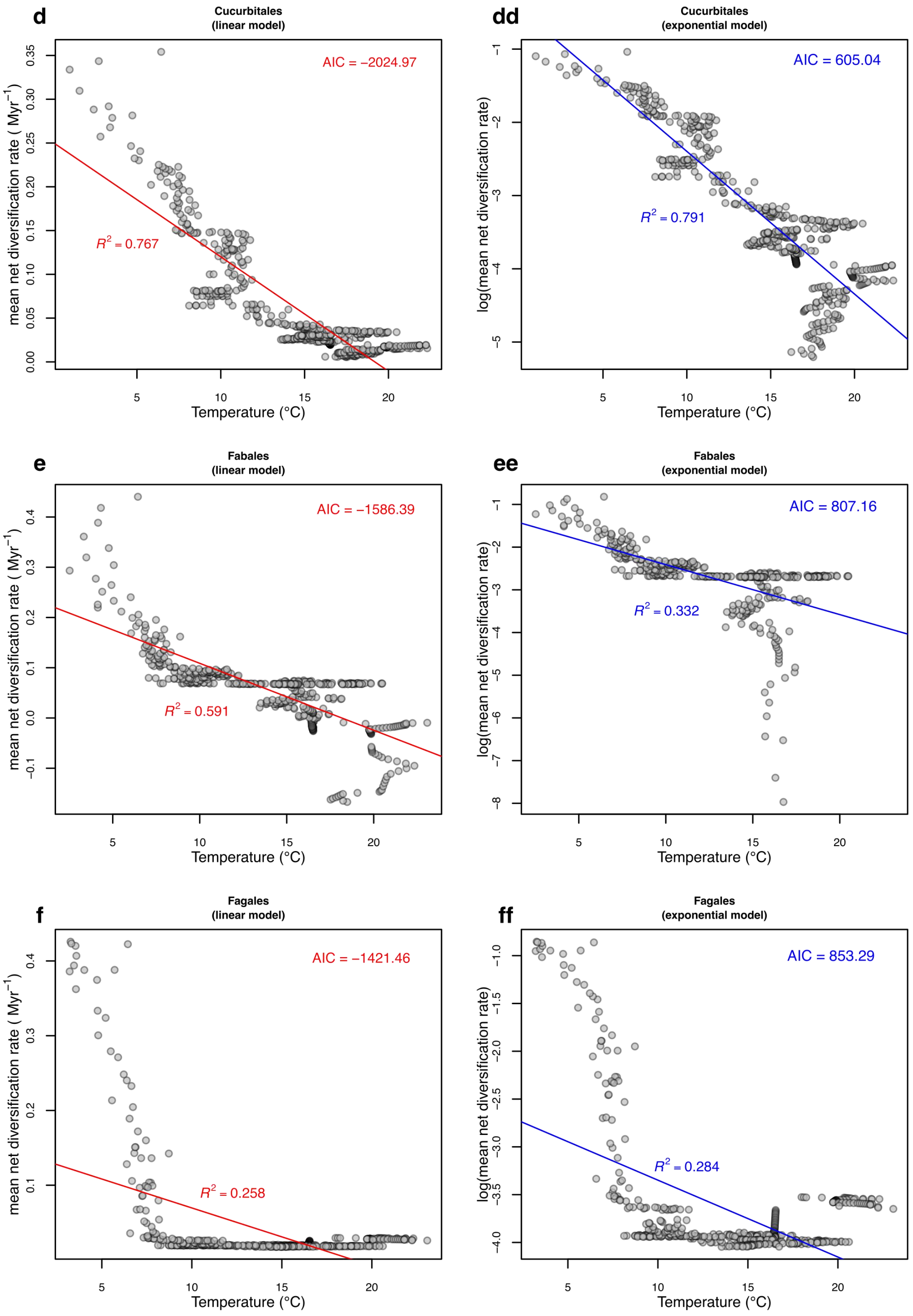
d
Cucurbitales
(linear model)
AIC = −2024.97
R^2 = 0.767
mean net diversification rate (Myr^{-1})
Temperature (°C)
dd
Cucurbitales
(exponential model)
AIC = 605.04
R^2 = 0.791
log(mean net diversification rate)
Temperature (°C)
e
Fabales
(linear model)
AIC = −1586.39
R^2 = 0.591
mean net diversification rate (Myr^{-1})
Temperature (°C)
ee
Fabales
(exponential model)
AIC = 807.16
R^2 = 0.332
log(mean net diversification rate)
Temperature (°C)
f
Fagales
(linear model)
AIC = −1421.46
R^2 = 0.258
mean net diversification rate (Myr^{-1})
Temperature (°C)
ff
Fagales
(exponential model)
AIC = 853.29
R^2 = 0.284
log(mean net diversification rate)
Temperature (°C)

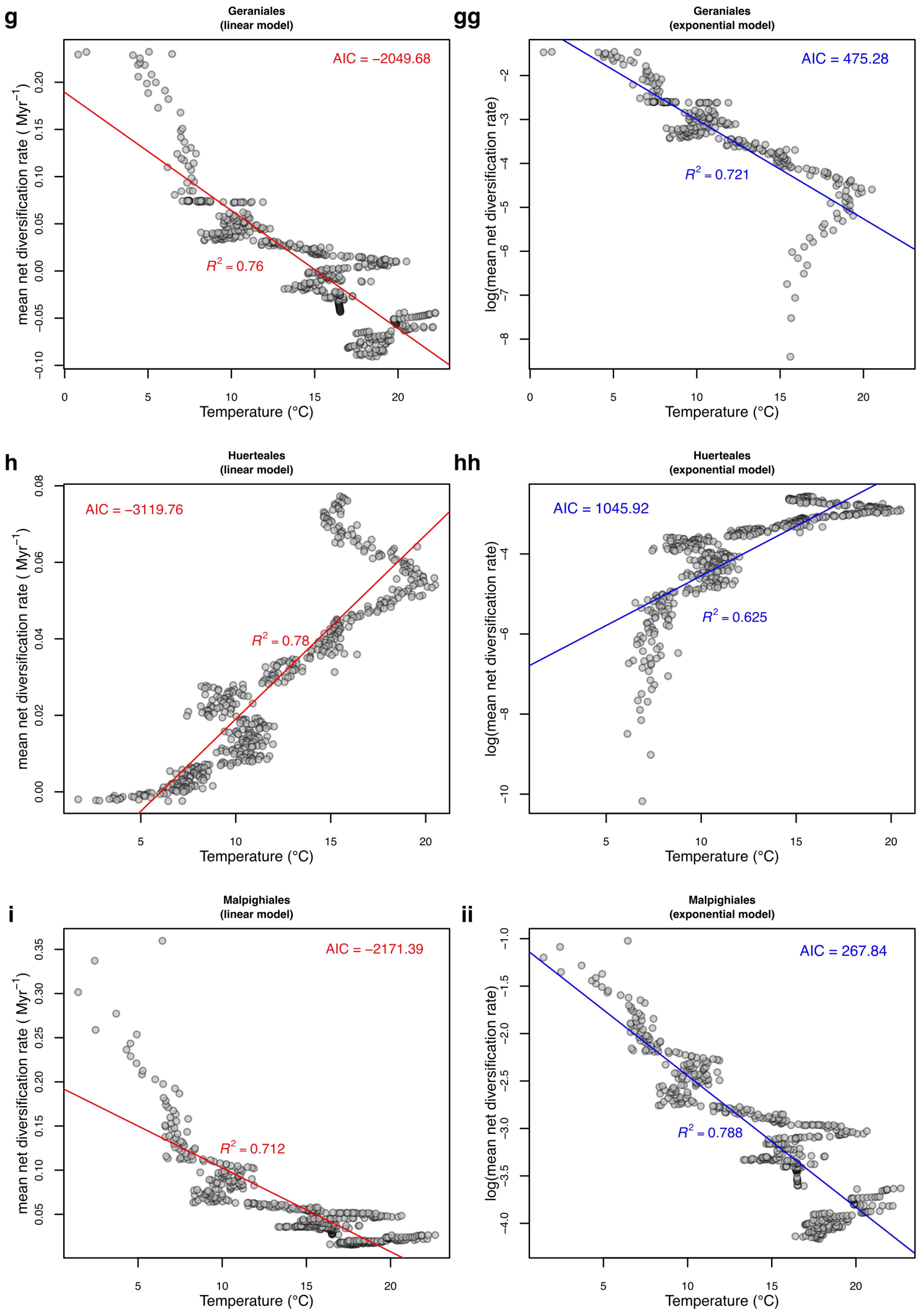

g
Geraniales
(linear model)
AIC = −2049.68
R² = 0.76
mean net diversification rate (Myr⁻¹)
Temperature (°C)
gg
Geraniales
(exponential model)
AIC = 475.28
R² = 0.721
log(mean net diversification rate)
Temperature (°C)
h
Huerteales
(linear model)
AIC = −3119.76
R² = 0.78
mean net diversification rate (Myr⁻¹)
Temperature (°C)
hh
Huerteales
(exponential model)
AIC = 1045.92
R² = 0.625
log(mean net diversification rate)
Temperature (°C)
i
Malpighiales
(linear model)
AIC = −2171.39
R² = 0.712
mean net diversification rate (Myr⁻¹)
Temperature (°C)
ii
Malpighiales
(exponential model)
AIC = 267.84
R² = 0.788
log(mean net diversification rate)
Temperature (°C)

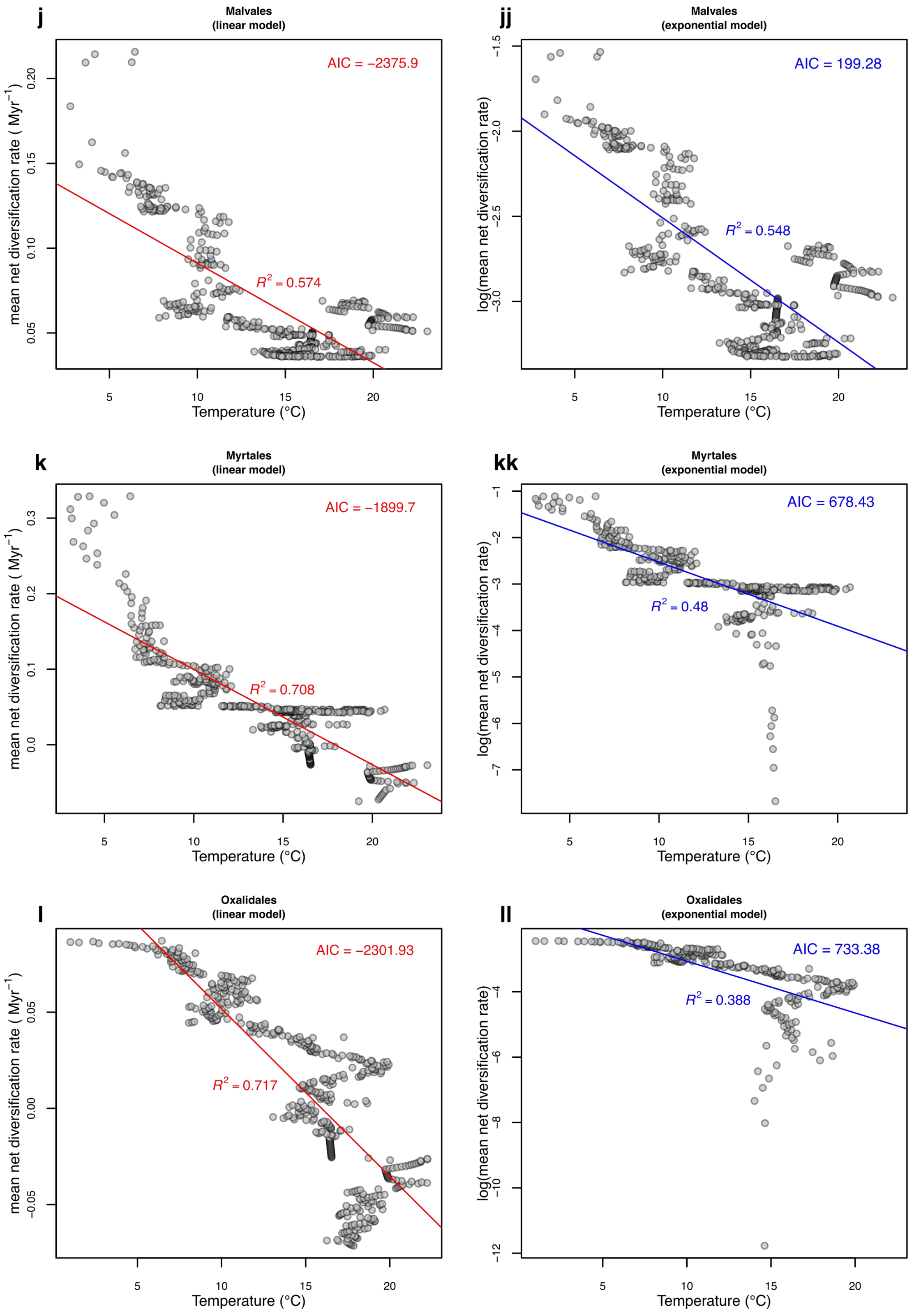
j
Malvales
(linear model)
AIC = −2375.9
R² = 0.574
mean net diversification rate (Myr⁻¹)
Temperature (°C)
jj
Malvales
(exponential model)
AIC = 199.28
R² = 0.548
log(mean net diversification rate)
Temperature (°C)
k
Myrtales
(linear model)
AIC = −1899.7
R² = 0.708
mean net diversification rate (Myr⁻¹)
Temperature (°C)
kk
Myrtales
(exponential model)
AIC = 678.43
R² = 0.48
log(mean net diversification rate)
Temperature (°C)
l
Oxalidales
(linear model)
AIC = −2301.93
R² = 0.717
mean net diversification rate (Myr⁻¹)
Temperature (°C)
ll
Oxalidales
(exponential model)
AIC = 733.38
R² = 0.388
log(mean net diversification rate)
Temperature (°C)

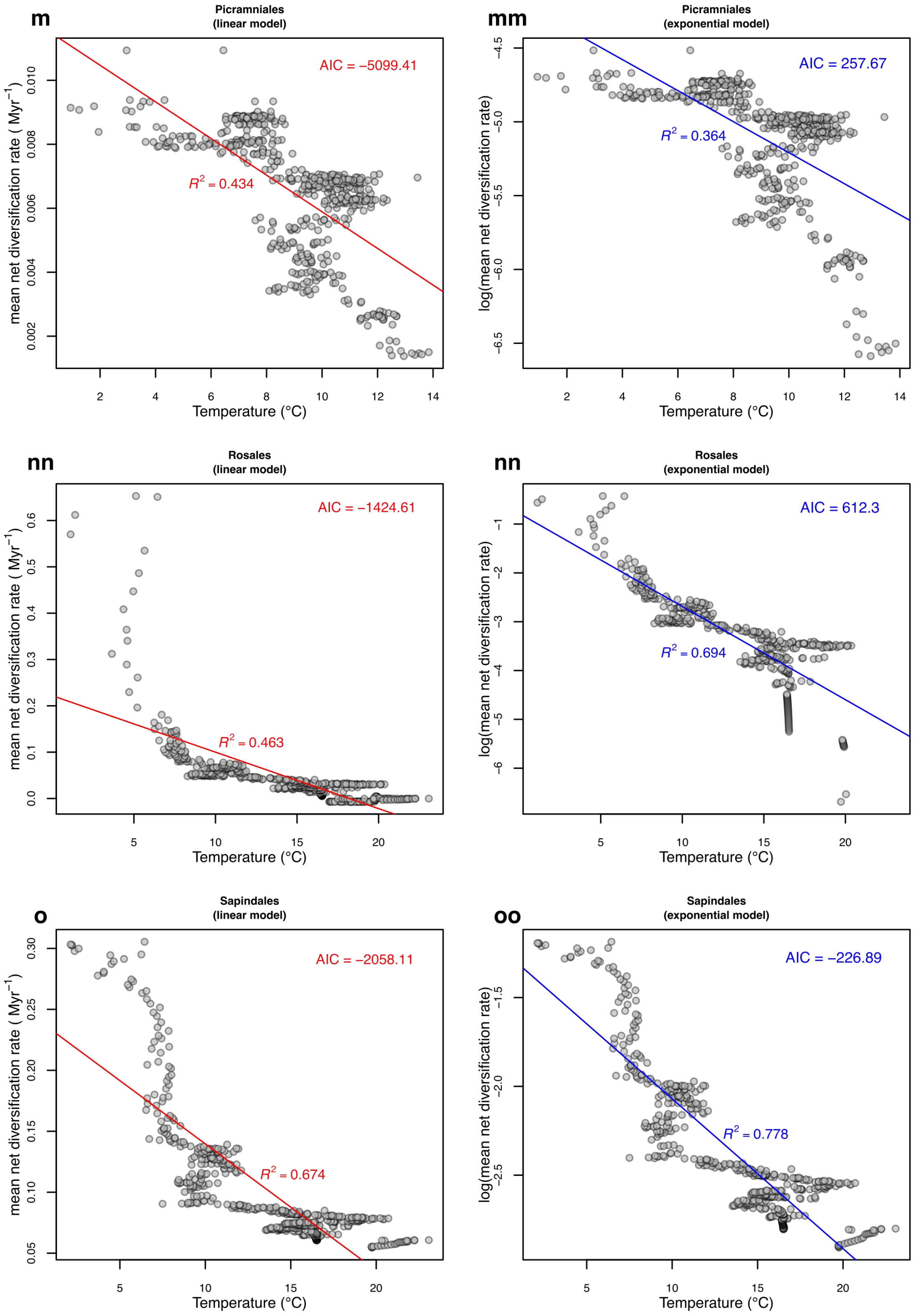
m
Picramniales
(linear model)
AIC = −5099.41
R^2 = 0.434
mean net diversification rate (Myr^{-1})
Temperature (°C)
mm
Picramniales
(exponential model)
AIC = 257.67
R^2 = 0.364
log(mean net diversification rate)
Temperature (°C)
nn
Rosales
(linear model)
AIC = −1424.61
R^2 = 0.463
mean net diversification rate (Myr^{-1})
Temperature (°C)
nn
Rosales
(exponential model)
AIC = 612.3
R^2 = 0.694
log(mean net diversification rate)
Temperature (°C)
o
Sapindales
(linear model)
AIC = −2058.11
R^2 = 0.674
mean net diversification rate (Myr^{-1})
Temperature (°C)
oo
Sapindales
(exponential model)
AIC = −226.89
R^2 = 0.778
log(mean net diversification rate)
Temperature (°C)

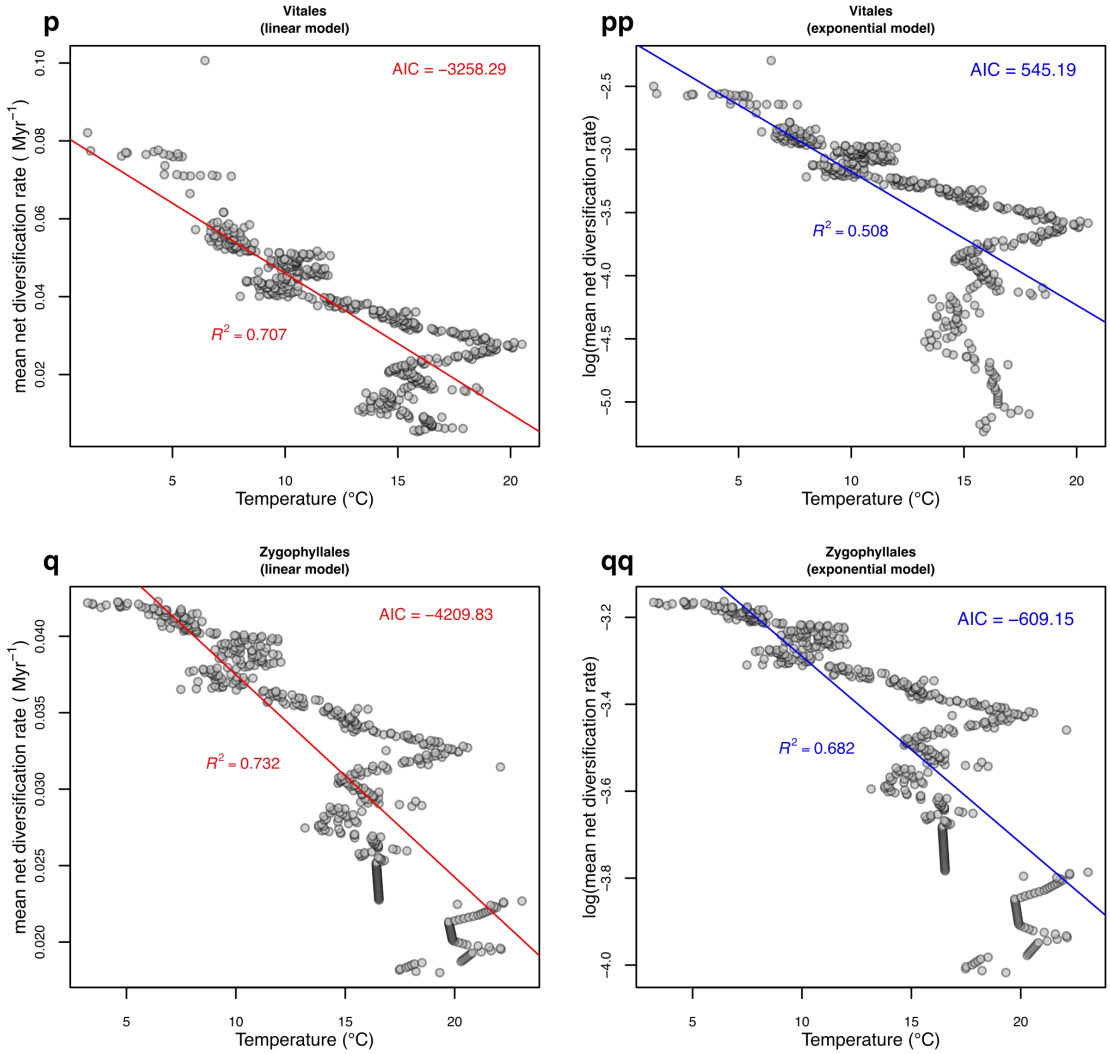

Supplementary Fig. 2. Correlation between BAMM net diversification rates in 17 rosid orders and historical global temperature based on linear and exponential best-fit models. Panels a-q represent the best linear model for each of 17 orders with AIC and R^2 as indicated; similarly, and panels aa-qq represent the best exponential model for 17 orders with AIC and R^2 as indicated; historical global temperature (5-point temperature means) since the Late Cretaceous (~113 Myr) is derived from ref[31].

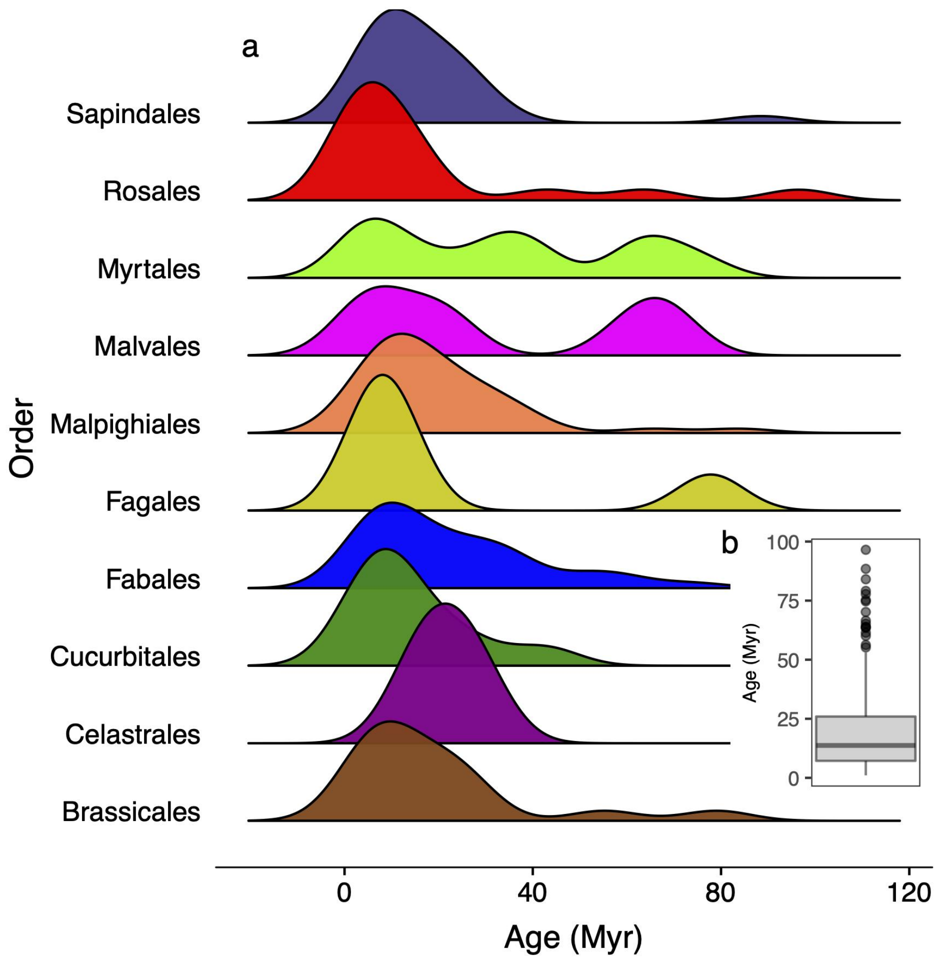

Supplementary Fig. 3. The age distribution of diversification rate shifts of 17 rosid orders estimated by BAMM. Panel a: Ridge plots of the age distribution of diversification rate shift for each rosid order; data from orders Geraniales and Vitales were removed from plotting because only 1 shift was detected in each; no shifts were detected for the orders Crossosomatales, Huerteales, Oxalidales, Picramniales, and Zygophyllales in our BAMM analyses. Panel b: Boxplot shows a summary of overall ages of each diversification shift detected across all 17 orders with time in "Myr" on the y-axis. N=182; Median—solid line in the box, box—interquartile range [25% and 75%], whiskers—5% and 95% intervals. Generally, the times of the overall diversification rate shifts are coincident with an earlier cooling period at the Eocene-Oligocene Glacial Maximum (~34 Myr; Panel a). Also, as shown in Fig. 2 in main text, diversification rates from most rosid orders show abrupt rate increases from ~15 Myr (around the Middle Miocene Climatic Optimum) to the present. Source data are provided in Source Data file.

Supplementary References

1. Chamberlain, S. & Boettiger, C. R, Python, and Ruby clients for GBIF species occurrence data. *PeerJ. PrePr.* **5**, e3304v1 (2017).
2. Chamberlain, S. *et al.* rgbif: Interface to the Global Biodiversity Information Facility API. R package version 1.3.0. https://CRAN.R-project.org/package=rgbif (2019).
3. Michonneau, F. & Collins, M. ridigbio: Interface to the iDigBio Data API. R package version 0.3.5. https://CRAN.R-project.org/package=ridigbio (2017).
4. Folk, R. A. *et al.* Rates of niche and phenotype evolution lag behind diversification in a temperate radiation. *Proc. Natl. Acad. Sci. U. S. A.* **116**, 10874–10882 (2019).
5. Owens, H. L. *et al.* The latitudinal diversity gradient in New World swallowtail butterflies is caused by contrasting patterns of out-of-and into-the-tropics dispersal. *Glob. Ecol. Biogeogr.* **26**, 1447–1458 (2017).
6. Rabosky, D. L. Automatic Detection of Key Innovations, Rate Shifts, and Diversity-Dependence on Phylogenetic Trees. *PLoS ONE* **9**, e89543 (2014).
7. Rabosky, D. L. *et al*. BAMMtools: an R package for the analysis of evolutionary dynamics on phylogenetic trees. *Methods Ecol. Evol.* **5**, 701–707 (2014).
8. Plummer, M., Best, N., Cowles, K. & Vines, K. CODA: convergence diagnosis and output analysis for MCMC. *R news* **6**, 7–11 (2006).
9. Jetz, W., Thomas, G. H., Joy, J. B., Hartmann, K. & Mooers, A. O. The global diversity of birds in space and time. *Nature* **491**, 444–448 (2012).
10. Harvey, M. G. *et al*. Population differentiation versus speciation rates. *Proc. Natl. Acad. Sci. U. S. A.* **114**, 6328–6333 (2017).
11. Rabosky, D. L. *et al.* An inverse latitudinal gradient in speciation rate for marine fishes. *Nature* **559**, 392–395 (2018).
12. Sun, M. *et al.* Estimating rates and patterns of diversification with incomplete sampling: A case study in the rosids. *Am. J. Bot.* (2020; In press).
13. Harvey, M. G. & Rabosky, D. L. Continuous traits and speciation rates: Alternatives to state-dependent diversification models. *Methods Ecol. Evol.* **9**, 984–993 (2017).
14. Rabosky, D. L. & Goldberg, E. E. FiSSE: A simple nonparametric test for the effects of a binary character on lineage diversification rates. *Evolution* **71**, 1432–1442 (2017).

15. Barnes, R. dggridR: Discrete Global Grids for R. R package version 0.1.12. https://github.com/r-barnes/dggridR/ (2017).

16. May, M. R. & Moore, B. R. How well can we detect lineage-specific diversification-rate shifts? A simulation study of sequential AIC methods. *Syst. Biol.* **65**, 1076–1084 (2016).

17. Moore, B. R., Höhna, S., May, M. R., Rannala, B. & Huelsenbeck, J. P. Critically evaluating the theory and performance of Bayesian analysis of macroevolutionary mixtures. *Proc. Natl. Acad. Sci. U.S.A.* **113**, 9569–9574 (2016).

18. Meyer, A. & Wiens, J. J. Estimating diversification rates for higher taxa: BAMM can give problematic estimate of rates and rate shifts. *Evolution* **72**, 39–53 (2017).

19. Meyer, A., Roman-Palacios, C. & Wiens, J. J. BAMM gives misleading rate estimates in simulated and empirical datasets. *Evolution* **72**, 2257-2266 (2018).

20. Rabosky, D. L, Mitchell, J. S & Chang, J. Is BAMM Flawed? Theoretical and Practical Concerns in the Analysis of Multi-Rate Diversification Models. *Syst. Biol.* **66**, 477–498 (2017).

21. Rabosky, D. L. BAMM at the court of false equivalency: a response to Meyer and Wiens. Evolution **72**, 2246–2256 (2018a).

22. Rabosky, D. L. Phylogenies and diversification rates: variance cannot be ignored. *Syst. Biol.* **68**, 538–550 (2018b).

23. Maddison, W. P., Midford, P. E., & Otto, S. P. Estimating a binary character's effect on speciation and extinction. *Syst. Biol.* **56**, 701–710 (2007).

24. Beaulieu, J. M. & O'Meara, B. C. Detecting Hidden Diversification Shifts in Models of Trait-Dependent Speciation and Extinction, *Syst. Biol.* **65**, 583–601 (2016).

25. Wagenmakers, E. J. & Farrell, S. AIC model selection using Akaike weights. *Psychon. Bull. Rev.* **11**, 192–196 (2004).

26. Kreft, H, & Jetz, W. Global patterns and determinants of vascular plant diversity. *Proc. Natl. Acad. Sci. U. S. A.* **104**, 5925–5930 (2007).

27. Rabosky, D. L. & Huang, H. A robust semi-parametric test for detecting traitdependent diversification. *Syst. Biol.* **65**, 181–193 (2016).

28. Morlon, H., Lewitus, E., Condamine, F. L., Manceau, M., Clavel, J. & Drury, J. RPANDA: an R package for macroevolutionary analyses on phylogenetic trees. *Methods Ecol. Evol.* **7**, 589–597 (2016).

29. Morlon, H. Phylogenetic approaches for studying diversification. *Ecol. Lett.* **17**, 508–525 (2014).
30. Sun, M. *et al.* Exploring the phylogeny of rosids with a five-locus supermatrix from GenBank. Preprint at https://www.biorxiv.org/content/10.1101/694950v1 (2019).
31. Cramer, B. S., Toggweiler, J. R., Wright, J. D., Katz, M. E. & Miller, K. G. Ocean overturning since the Late Cretaceous: Inferences from a new benthic foraminiferal isotope compilation. *Paleoceanography* **24**, PA4216 (2009).
32. Condamine, F. L., Rolland, J. & Morlon, H. Macroevolutionary perspectives to environmental change. *Ecol. Lett.* **16**, 72–85 (2013).
33. Akaike, H. A new look at the statistical model identification. *IEEE Transactions on Automatic Control* **19**, 716–723 (1974).
34. Hochberg, Y. A sharper Bonferroni procedure for multiple tests of significance. *Biometrika* **75**, 800–803 (1988).
35. APG IV. An update of the Angiosperm Phylogeny Group classification for the orders and families of flowering plants: APG IV. *Bot. J. Linn. Soc.* **181**, 1–20 (2016).
