# Supplementary material for: Recent accelerated diversification in rosids occurred outside the tropics
Source: Nat Commun. 2020 Jul 3;11:3333. doi: 10.1038/s41467-020-17116-5 (PMC7335165; doi:10.1038/s41467-020-17116-5)
Supplement: Supplementary file 3 — Reporting Summary [file 41467_2020_17116_MOESM3_ESM.pdf]

nature research

Corresponding author(s): Dr. Miao Sun, Dr. Ryan A. Folk, and Dr. Rob P. Guralnick

Last updated by author(s): May 28, 2020

Reporting Summary

Nature Research wishes to improve the reproducibility of the work that we publish. This form provides structure for consistency and transparency in reporting. For further information on Nature Research policies, see Authors & Referees and the Editorial Policy Checklist .

Statistics

For all statistical analyses, confirm that the following items are present in the figure legend, table legend, main text, or Methods section.

n/a	Confirmed	
☐	☒	The exact sample size (n) for each experimental group/condition, given as a discrete number and unit of measurement
☒	☐	A statement on whether measurements were taken from distinct samples or whether the same sample was measured repeatedly
☐	☒	The statistical test(s) used AND whether they are one- or two-sided<br>*Only common tests should be described solely by name; describe more complex techniques in the Methods section.*
☒	☐	A description of all covariates tested
☐	☒	A description of any assumptions or corrections, such as tests of normality and adjustment for multiple comparisons
☐	☒	A full description of the statistical parameters including central tendency (e.g. means) or other basic estimates (e.g. regression coefficient) AND variation (e.g. standard deviation) or associated estimates of uncertainty (e.g. confidence intervals)
☐	☒	For null hypothesis testing, the test statistic (e.g. F, t, r) with confidence intervals, effect sizes, degrees of freedom and P value noted<br>*Give P values as exact values whenever suitable.*
☐	☒	For Bayesian analysis, information on the choice of priors and Markov chain Monte Carlo settings
☒	☐	For hierarchical and complex designs, identification of the appropriate level for tests and full reporting of outcomes
☐	☒	Estimates of effect sizes (e.g. Cohen's d, Pearson's r), indicating how they were calculated

Our web collection on statistics for biologists contains articles on many of the points above.

Software and code

Policy information about availability of computer code

Data collection

Sequence data were collected from GenBank (Release 214: June 15, 2016) using PHLAWD (version 3.4a, https://github.com/blackrim/phlawd; Smith et al., 2009) in Sun et al. (2019; https://www.biorxiv.org/content/10.1101/694950v2.full).

Divergence time estimation was conducted via treePL v.1.0 (Smith & O'Meara, 2012) in Sun et al. (2019; https://www.biorxiv.org/content/10.1101/694950v2.full).

Species occurence data were collected from iDigBio and GBIF using R packages rgbif v1.3.0 (Chamberlain & Boettiger, 2017; Chamberlain et al., 2019) and ridigbio v0.3.5 (Michonneau & Collins, 2017) on June 4th, 2019

Global mean annual temperature data (bio1 from BioClim), were downloaded from the WorldClim website.

The climatic tropicality dataset used the standard Köppen-Geiger climatic tropics definition as calculated by Owens et al. (2017:https://onlinelibrary.wiley.com/doi/full/10.1111/geb.12672)

Species occurrence and temperature data cleaning use Python scripts from Folk et al. (2019) and custom R scripts from https://github.com/Cactusolo/rosid_NCOMMS-19-37964-T (doi:10.5281/zenodo.3843441).

Historical oxygen isotope (δ18O) data were derived from from Cramer et al. (2009).

Data analysis

Diversification analyses were performed in RPANDA v1.4 (Morlon et al., 2016), BAMM v2.5.0 (Rabosky, 2014), BAMMtools (Rabosky et al., 2014), and DR (Jetz et al., 2012). Diversification and Temperature traits correlation tests were performed in es-SIM (Harvey & Rabosky, 2017), FiSSE (Rabosky & Goldberg, 2017), and STRAPP (Rabosky & Huang, 2016). Phylogenetic niche conservatism tests were performed in R package phytools v0.7-00 (Revell, 2011) and geiger v2.0.6.2 (Pennell et al., 2014). Hidden-state speciation and extinction (HiSSE) models were carried using R packages "hisse" v1.9.6 (Beaulieu & O'Mearara, 2016). Spatial analysis of species richness, age and

diversification rate was performed in R package “dggridR” v. 2.0.3 (Barnes, 2017). Custom R scripts are available from https://github.com/Cactusolo/rosid_NCOMMS-19-37964-T (doi:10.5281/zenodo.3843441).

For manuscripts utilizing custom algorithms or software that are central to the research but not yet described in published literature, software must be made available to editors/reviewers. We strongly encourage code deposition in a community repository (e.g. GitHub). See the Nature Research guidelines for submitting code & software for further information.

Data

Policy information about availability of data

All manuscripts must include a data availability statement. This statement should provide the following information, where applicable:

- Accession codes, unique identifiers, or web links for publicly available datasets
- A list of figures that have associated raw data
- A description of any restrictions on data availability

No data restrictions. The data used in this analysis is deposited in https://github.com/Cactusolo/rosid_NCOMMS-19-37964-T (doi:10.5281/zenodo.3843441). For phylogeny and dating data and information please see: Sun M, Folk RA, Gitzendanner MA, Smith SA, Germain-Aubrey C, Guralnick RP, Soltis PS, Chen ZD, Soltis DE. Exploring the phylogeny and diversification of rosids with a five-locus supermatrix. bioRxiv 694950. https://www.biorxiv.org/content/10.1101/694950v2.full. And some methods related to phylogentic uncertainties: Sun M, Folk RA, Gitzendanner MA, Soltis PS, Chen ZD, Soltis DE, Guralnick RP. (2020). Estimating rates and patterns of diversification with incomplete sampling: A case study in the rosids. American Journal of Botany (Accepted; also see bioRxiv 749325).

The climatic tropicality dataset used the standard Köppen-Geiger climatic tropics definition as calculated by Owens et al. (2017:https://onlinelibrary.wiley.com/doi/full/10.1111/geb.12672)

Dated phylogenetic trees (molecular data mined from NCBI: https://www.ncbi.nlm.nih.gov), species distribution data, WorldClim temperature data (downlaoded from https://www.worldclim.org), and the BAMM diversification rate-through-time matrix are available in “Datasets” folder from https://github.com/Cactusolo/rosid_NCOMMS-19-37964-T (doi:10.5281/zenodo.3843441); the source data underlying Fig. 2, piechart of Fig. 3, and Supplementary Fig. 3 are provided as a Source Data file; all other relevant intermediate data is available from the authors upon request.

Field-specific reporting

Please select the one below that is the best fit for your research. If you are not sure, read the appropriate sections before making your selection.

☐ Life sciences ☐ Behavioural & social sciences ☒ Ecological, evolutionary & environmental sciences

For a reference copy of the document with all sections, see nature.com/documents/nr-reporting-summary-flat.pdf

Ecological, evolutionary & environmental sciences study design

All studies must disclose on these points even when the disclosure is negative.

Study description	We built large-scale phylogeny; then ran diversification analyses using three different contemporary methods, and calculated species-specific diversification rates; meanwhile we also assemble four temperature datasets (two continuous; two binary; one paleoclimate data, the other are more present) to investigate how diversification in rosid correlated with its temperature niche (tropical vs. non-tropical); we did sampling bias sensitivity tests, and multiple correlation test for continuous traits and binary traits, and linear and exponential regression model test. All these analyses were replicated across 17 globally distributed rosid taxonomic orders to cross-check the pattern observed between diversification rates and temperature traits.
Research sample	1.We assembled a 20294-taxon, 5-locus supermatrix for rosid phylogeny, represent 17 (100%) rosid orders and 135 (100%) families and 3,070 genera (66.34% of OpenTree)<br>2. We collected 2,794,941 rosid occurrence records represent 18,283 species; each species had an average of 160 occurrence points<br>3. We collected 762,043 mean annual temperature records (bio1 from WorldClim), represent 16,986 rosid species<br>4. We collected 1,468,271 Köppen-Geiger climatic data records, representing 17,635 rosid species
Sampling strategy	1. Phylogenetic sampling reflects the top 5 most widely sequenced loci available in GenBank;<br>2. The 17 rosid orders and 135 families followed the most updated APG system were completely represented;<br>3. All the species distribution records from iDigBio and GBIF for all the rosid species sampled in the phylogeny were collected and combined.
Data collection	Sequence data were collected from GenBank (Release 214: June 15, 2016) using PHLAWD (version 3.4a, https://github.com/blackrim/phlawd; Smith et al., 2009).<br><br>Species occurence data were collected from iDigBio and GBIF using R packages rgbif v1.3.0 (Chamberlain & Boettiger, 2017; Chamberlain et al., 2019) and ridigbio v0.3.5 (Michonneau & Collins, 2017) on June 4th, 2019<br><br>Global mean annual temperature data (bio1 from BioClim), were downloaded from the WorldClim website (https://www.worldclim.org/).<br><br>Historical oxygen isotope (δ18O) data were derived from from Cramer et al. (2009).

Timing and spatial scale	Not applicable
Data exclusions	1. For the phylogeny, we pruned sequence data with "subsp.", "var.", "f.", "cf." and "aff." designations in taxon names (to preserve one sample per species) and removed rogue taxa based on manual inspection of initial phylogenies for spurious taxon placement;<br>2. Outgroups were remove for downstream diversification analyses as these are outside the group of interest for the study;<br>3. For distributional data, any coordinates with zero latitude and longitude were excluded, as well as suspect records and geographic outliers;<br>4. For temperature trait datasets, we excluded any points with missing data;<br>5. For spatial analyses on tip rates and community age, we excluded any small communities (<3 species) in spatial grid cell.
Reproducibility	No manipulative experiments were performed in our study. All our methods are detailed in the materials and methods section and Supplementary files; All the scripts used in present study is available on GitHub at https://github.com/Cactusolo/rosid_NCOMMS-19-37964-T (doi:10.5281/zenodo.3843441).
Randomization	1. Random cross-validation was used in treePL analyses.<br>2. Given evidence for over-representation in some non-tropical areas, we implemented a sensitivity analysis by randomly dropping 10%, 30%, or 50% of non-tropical species.
Blinding	Not applicable

Did the study involve field work? ☐ Yes ☒ No

Reporting for specific materials, systems and methods

We require information from authors about some types of materials, experimental systems and methods used in many studies. Here, indicate whether each material, system or method listed is relevant to your study. If you are not sure if a list item applies to your research, read the appropriate section before selecting a response.

Materials & experimental systems

n/a	Involved in the study
☒	☐ Antibodies
☒	☐ Eukaryotic cell lines
☒	☐ Palaeontology
☒	☐ Animals and other organisms
☒	☐ Human research participants
☒	☐ Clinical data

Methods

n/a	Involved in the study
☒	☐ ChIP-seq
☒	☐ Flow cytometry
☒	☐ MRI-based neuroimaging
