# Supplementary material for: Recent accelerated diversification in rosids occurred outside the tropics
Source: Nat Commun. 2020 Jul 3;11:3333. doi: 10.1038/s41467-020-17116-5 (PMC7335165; doi:10.1038/s41467-020-17116-5)
Supplement: Supplementary file 2 — Peer Review File [file 41467_2020_17116_MOESM2_ESM.pdf]

Reviewers' comments:

Reviewer #1 (Remarks to the Author):

The paper looks at correlations between temperature (incl. historical temperature) with diversification in rosids, one of the main clades of flowering plants. They found that rosid diversification is negatively correlated with temperature, and hence argue that they must have diversified outside the tropics (although most of their diversity is currently found within the tropics).

Using a 5 locus-based phylogenetic tree, twice previous sampling for rosids, i.e. nearly 20,000 terminals, they conducted a series of analyses. They were able to link about 17,000 taxa to three measures of tropicality (incl. temperature). Also, they looked at alternate models with paleo temperature data – and found negative correlations of diversification with paleo temperature. However, the authors also explain that they had overrepresentation of temperate taxa, which could bias the results towards their findings. I am unclear as to how they excluded this possibility with confidence, although they mention a sensitivity analysis dropping taxa in turn.

This is a large piece of work but I am left unclear with the following: How resolved is a 5-loci tree, why not using more loci? How results are different from Sun et al.; I can see it does not look at the same analyses than here, but still the taxon sampling is in the same order of magnitude (not twice smaller as explained here, or am I missing something)? What is the effect of geographic sampling bias, how confident can we be in the sensitivity analysis? Crucially, what actually drove diversification in rosids, if say, it was outside the tropics? Could you not test hypotheses along these lines (topographic heterogeneity, glaciations, refugia, etc), in addition to having shown negative correlation with temperature and tropicality? It is definitely a lot of work achieved already.

Reviewer #2 (Remarks to the Author):

Understanding large-scale spatiotemporal variations of macroevolutionary rates is a major goal of evolutionary science and a critical piece of the puzzle for understanding global patterns such as the latitudinal diversity gradient. Although the history of this literature is long, recently available datasets and methods are producing novel perspectives on the drivers of macroevolutionary rates.

This contribution by Sun et al. examining diversification rates vs. temperature and latitude of the largest clade of flowering plants would be a welcome addition to the literature, and should be of general interest. Many hypotheses regarding the mechanisms underlying diversification rates with latitude predict higher speciation rates in areas with high temperatures. In contrast, Sun et al. find there is a negative relationship between temperature and diversification rate, both spatially around the globe with regards to contemporary climate, and historically as the climate has cooled. This paper follows a series of recent studies across taxa that find either no correlation with latitude or high speciation/diversification rates in high latitudes. So, the result is consistent with recent results from other taxa even if it is not predicted a priori by theory. This is exciting and timely.

I have a number of comments to help improve the ms.

1) Line 256: The authors cite 9 papers in the discussion apparently showing increased diversification rates associated with global cooling in plants. First, this seems like it might be mentioned in the introduction, since it is background context for the study. Second, if this pattern is widely documented, perhaps a more clear explanation of the novelty of the current analysis would be warranted as well.

2) The opening sentences relate to the rise of angiosperms in the Cretaceous and explanations for their diversity. This is perhaps a stylistic issue but I feel this opener was discordant from the rest of the paper, which isn't about the rise of the angiosperms and overall success but rather about geographic and environmental correlates of the their (especially more recent than Cretaceous) diversification patterns.

3) [note updated below] I am concerned about the lack of clade-specific missing data correction, if that is correct (I'm not sure). Is there a reason that clades were not corrected for missing data on an individual basis? Typically, within a large taxonomic group, some clades are better studied than others and have more molecular data available, and this can vary dramatically depending on which clades had been the subject of large projects.

-update, in the results I see that this correction was applied specifically tailored to each clade, so my point doesn't apply. I'm leaving this comment so that perhaps the methods can describe this more clearly, by noting that it was clade specific and not for rosids as a whole in the main paper.

4) Line 289: This paper has many analyses of diversification rate, not just speciation rate, so this statement is a bit strange.

5) Passage line 242-248: I am having trouble following this argument or how it relates to the results.

6) One important thing about the results is that statistical correlations with temperature and latitude don't seem to hold within order, but hold across order (or pooled across data), if I interpret table 1 correctly. This is despite the fact that the orders should be large enough to have statistical power to find these correlations. The authors don't seem to explain or interpret this. This seems an important part of the conclusions, and I am not myself sure what it means.

7) Line 338: "All analyses were run in parallel across these subtrees." This is a bit unclear, does this mean BAMM and all div rate analyses were inferred totally independently? I have no real objection to doing it this way, but is there a reason why you did this instead of analyzing it all at once?

Update- I found it in the supplement that this was done for computational efficiency reasons. I think since it is an important decision in how the models were run, you could add the phrase "For computational reasons, it is difficult to run diversification rate analyses on the whole rosid clade, thus we analyzed each order independently" or something to that effect.

8) It is also unclear how STRAPP was applied to the whole dataset if runs were independent, was it combining the independent clade-wise runs somehow? That would seem problematic since one would not know whether different parts of the tree run independently would have the same macroevolutionary process (important in the permutation test)

9) Line 379: Should the title of this section be something more descriptive, along the lines of "Diversification rate inference and statistical analysis"

10) Table 1. Many of the adjusted p values are identical across taxa. Is this explainable somehow due to how the mult-comp corrections work?

11) Table 1: I also feel somehow you should put the sign of the result of the FiSSE analysis on the table, so people can clearly interpret what biological finding the p values refer to.

12) Line 105: A little more background on the macroscale patterns in rosid diversity would be helpful in the introduction. The authors say "community-level" species richness but don't directly address how richness varies for example, by latitudinal band. Are there more rosid species present globally

between 0 to 10 degrees than between 30 to 40 degrees? This seems important information for the reader to interpret the results.

13) Line 387: How was BAMM used to examine correlations between historical temperature and diversification rates statistically? The methods refer to supplementary section M3 but in there it just describes how BAMM was run. STRAPP can be used for relating tip rates with traits, and BAMM can be used to plot rates vs time, but it is unclear how BAMM was used to asses historical correlations between temperature or time and diversification rates as RPANDA does (or was it just compared visually?).

14) Are there other studies that found the Miocene cooling (other than Folk 2019) to be an important period for buildup of extant temperate diversity, or is this the first one? The Oligocene cooling is most commonly discussed as a prediction of the tropical conservatism hypothesis, I believe.

15) The phylogenetic and geographic data are necessarily a bit fragmentary relative to the size of the group, as acknowledged by the authors. This is unavoidable at this stage and I think the authors do a good job overall of making use of available resources.

The phylogeny in particular was constructed by stretching available sequence data, with many species added to the tree with only a single locus, and (I think) all analyses depend on a single topology and dating of the tree using penalized likelihood, that does not represent uncertainty in topology or clade ages or pass that forward to the analysis. Using a single topology and dating has previously been a point of criticism in large megaphylogeny projects (e.g. Title et al. 2016).

The authors largely discuss the tree reconstruction in their companion paper, which I have also read. They also do quality control by comparing topology and clade ages to other studies, which seems to me to be a good approach to cross-validation. I think it would help the current ms to add a few sentences to the methods describing quality control and robustness of the tree.

16) Line 290: Citing Folk et al 2019 is probably not appropriate here, as this idea has been around for much longer.

17) One limitation of the study is the authors did not analyzes ages of clades inside and outside the tropics, such as for example e.g. assess “diversification time” to complement the diversification rate analysis (e.g. Kerkhoff 2014 PNAS did something like this for plants). It would be interesting to know if all the temperate clades are old and only had recently accelerated diversification (seems fro fig 1 this could be the case), or whether many transitions out of the tropics occurred recently. In lieu of new analyses, some discussion of other papers/citation could address this issue.

Reviewer #3 (Remarks to the Author):

Sun et al. investigate the diversification dynamics of a large angiosperm clade, the rosids, in relation to temperature niche and tropical/temperate distributions. They find that diversification is highest in low temperatures and in temperate/arctic places, but only when testing the models for the whole rosid clade, in their subclade (order) approach they only find significance for one or two lineages, depending on the test (see Table 1). If true, this result is interesting, quite novel for angiosperms (especially for such a large clade which also includes very important tropical elements) and will raise awareness and discussion in the fields of plant evolution and ecology.

I applaud their efforts of collecting these large datasets and cleaning millions of occurrence records, in addition to using a previously published phylogenetic tree of the rosids. However, I have a couple of

major issues which make me unsure about the reliability of the results and main conclusion. Of course, understanding past dynamics will always be challenging because we base it on correlation and inferences of past speciation/extinction rates, but in my opinion it would be essential to base these correlative tests on a solid hypothesis which also finds support in the experimental or physiological literature, for example.

This directly relates to my first major concern. Such a hypothesis is missing. Many studies have tested the link between diversification and temperature or tropical/temperate across many taxonomic groups and found contrasting results (outlined nicely in the introduction). However, not many studies explicitly present the diversification mechanism for the link between temperature or tropical/temperate niche and diversification dynamics. One hypothesis is that high temperatures would lead to high mutation rates and this may facilitate speciation. But when hypothesising that cold temperatures lead to high diversification, a solid hypothesis is missing. In the discussion the authors briefly touch on a possible mechanism by suggesting that Late Miocene opening up of temperate niches provided ecological opportunities for temperate radiations. This is possible, and the RPanda and BAMM results (Fig. 2) could provide support for this idea, but I would prefer to see it better integrated within the manuscript. It also means that temperature by itself is not a diversification driver, it’s the opportunity of empty niches, which happened to be in temperate regions from the Late Miocene onwards. So to fully test this hypothesis, the manuscript would need rewording and a more sophisticated way to identify empty niches or ecological opportunities through time. The issue with this idea is that these temperate opportunities also led to reduction in tropical biome areas and opportunities, which may have simultaneously led to extinctions in tropical rosids. So it would be a challenge to identify whether the Late Miocene diversification increase is an artefact of not being able to see the extinct tropical lineages in the phylogeny, or whether it is a true signal. This could be done with simulations in which one simulates trees under constant speciation and extinction rates for both temperate and tropical lineages, and then selectively drops tropical lineages during the Miocene/Pliocene from the tree, without changing speciation parameters in the simulation, and then repeating the analyses. A script to do this is provided by this study: https://royalsocietypublishing.org/doi/full/10.1098/rspb.2018.0882?url_ver=Z39.88-2003&rfr_id=ori:rid:crossref.org&rfr_dat=cr_pub=pubmed.

In addition, it is unclear from the BAMM plots (Fig. 2) whether both tropical and temperate lineages increased in diversification during the Miocene/Pliocene. It would be nice to distinguish between those in the plots, because alternatively, all rosids increased diversification during this time due to other, non-measured, time-dependent factors.

My second major concern relates to the use of parametric and semi-parametric tests, rather than using more process-based models such as the BiSSE-type. The used tests use the tip speciation rates to test for a correlation with for example present-day average temperature, but this does not actually link the process of speciation to these characteristics (temperature, temperate/tropical). BiSSE methods have been criticised, but several solutions have been proposed (e.g. including hidden states, as well as using simulations). It would illustrate much better whether the different rosid clades consistently through time show higher speciation under lower temperatures. With such a large clade you would also have the statistical power to test whether rates indeed change during the Miocene/Pliocene, as compared to the earlier Cenozoic (i.e. with time-dependent BiSSE models).

Another concern is that results remain largely not significant, unless the whole rosid clade is considered. Why do subclades not show consistent significant patterns as well – could it be that it is only found for the rosids as a whole because such a large clade with so many tips would always show significance in a (semi-)parametric test? And why do the orders separately in most cases not show any significance?

Minor concerns:

-Tropical areas also include high-alpine, low temperature mountains, which have been often associated with high speciation rates due to ecological opportunities with mountain uplift. It would be good to mention this, or perhaps it could also explain some of the differences found when taking average mean temperature vs. tropical/temperate classifications.

-In addition to taking the mean temperature for a species, would it make sense to take a measure of temperature seasonality? If indeed Miocene climate changes and ecological opportunities were the driver of speciation, then it may be more seasonality rather than average temperature underlying speciation (e.g. though processes of reproductive isolation and allopatric speciation).

-Assignment of species to tropical/temperature was based on the average occurrence or dominant occurrence in one of these areas – how many species have occurrences ranging in both temperate/tropical? Would it be possible to identify those species as wide range in for example a GeoSSE diversification approach? Because it seems those are not physiologically/biologically limited to tropical or temperate biomes and this would be interesting to evaluate (rather than ignore as currently done) as well.

-The order of diversification methods used is not consistent between the results and the methods, this is confusing. The results section needs a sentence or two to introduce the method used, because it's currently a sum-up of p-values and not clear to the reader what was tested exactly and how.

-RPanda results support the "means temperature (x) dependent birth-death model with constant speciation and extinction rates" – can you explain in a bit more words what this biologically means? Also, Table S6 shows very extreme speciation and extinction rate values for certain clades (e.g. Picramniales) suggesting that something went wrong when fitting the model? Do results suggest that speciation rates are higher and extinction rates lower with higher temperatures? Because that would conflict with your conclusions, but it's a bit unclear how to interpret these values, so please clarify. Also, in the discussion you mention that extinction rates were not considered, but this seems not the case for this method?

-The purpose of the sensitivity analyses is not entirely clear – you repeat the STRAPP analyses with different sampling schemes, but (almost) none of the STRAPP analyses for the orders was significant in the empirical results, so how does the sensitivity analyses contribute to this?

Renske Onstein

REVIEWERS' COMMENTS:

Reviewer #1 (Remarks to the Author):

The paper looks at correlations between temperature (incl. historical temperature) with diversification in rosids, one of the main clades of flowering plants. They found that rosid diversification is negatively correlated with temperature, and hence argue that they must have diversified outside the tropics (although most of their diversity is currently found within the tropics).

Using a 5 locus-based phylogenetic tree, twice previous sampling for rosids, i.e. nearly 20,000 terminals, they conducted a series of analyses. They were able to link about 17,000 taxa to three measures of tropicality (incl. temperature). Also, they looked at alternate models with paleo temperature data – and found negative correlations of diversification with paleo temperature. However, the authors also explain that they had overrepresentation of temperate taxa, which could bias the results towards their findings. I am unclear as to how they excluded this possibility with confidence, although they mention a sensitivity analysis dropping taxa in turn.

> *The approach we used was essentially to simulate a case where we quite dramatically shifted the balance of sampling bias of non-tropical and tropical species via sequentially, randomly removing 10%, 30%, or 50% of non-tropical species while all tropical species were retained, across all orders. The purpose of this simulation was to generate alternative levels of tropical bias in our empirical dataset and assess the robustness of the results. In the most extreme case of a 50% removal of non-tropical species, where tropical taxa numerically dominate, we still find elevated diversification rates in non-tropical taxa; indeed, the pattern we observed barely changed under these dramatic sampling perturbations. We argue that this approach strongly provides the needed evidence that moderate amounts of geographic sampling bias are not likely to have a significant effect on estimation of diversification based on tip rates as implemented here. While we have not seen these sorts of sensitivity analysis performed previously to account for geographic biases, we note that this result may not be all that surprising if indeed sampling bias is largely independent of the phylogenetic distribution of the tropical/non-tropical trait states, as we note in the manuscript. We make sure this point is made even more forcefully in the revision.*

This is a large piece of work but I am left unclear with the following: How resolved is a 5-loci tree, why not using more loci? How results are different from Sun et al.; I can see it does not look at the same analyses than here, but still the taxon sampling is in the same order of magnitude (not twice smaller as explained here, or am I missing something)?

The five markers we include here represent the most widely used loci in angiosperm molecular systematics as deposited in GenBank. The inclusion of more loci would result in a precipitous increase in missing data and poor phylogenetic resolution as additional loci are sequenced for very few of these taxa.

The phylogeny resolved in the present study is largely in agreement with Sun et al. (2016), but with higher phylogenetic resolution as well as about double the taxon sampling (tree in Sun et al. 2016: n = *8,855; new tree:* n = *19,740). Particularly, all 135 rosid families we sampled are resolved as monophyletic in the newer study. Please see Sun et al. (2019a) for more details.*

What is the effect of geographic sampling bias, how confident can we be in the sensitivity analysis?

*We believe this question is similar to the same reviewer's query above concerning "*overrepresentation of temperate taxa*"; we refer our reviewer to the corresponding response above where we discuss the sensitivity analysis and its strength and value.*

Crucially, what actually drove diversification in rosids, if say, it was outside the tropics?

Our results support global cooling as a driver of diversification via conserved non-tropical temperature niches, an argument we attempted to make in the original version of the manuscript. In revising the manuscript comprehensively in response to all the reviewer comments, we have taken special care to bring this point further forward especially in the abstract and a now sharper discussion. Additionally, using supplementary analyses requested by reviewers, we find the unsurprising result that diversification had multiple drivers of which climate change and conserved temperature niche are only two; see our new results from HiSSE (hidden character state analyses; Table S4 in Supporting Information).

Could you not test hypotheses along these lines (topographic heterogeneity, glaciations, refugia, etc), in addition to having shown negative correlation with temperature and tropicality? It is definitely a lot of work achieved already.

Our work provides evidence that climate cooling and conservation of species' temperature niche play key roles in the diversification pattern of rosids, but by no means in isolation, as we also have evidence for unobserved (i.e., 'hidden' and unknown) parameters driving diversification. Still, we believe the emphasis on responses to long-term global climate cooling is warranted based on the current literature and assures a focused contribution for what is

already a large dataset and complex set of analyses. The factors identified by the reviewer would certainly make for excellent follow-up papers, but data are not readily available for these factors, and analysis of these potential drivers will require careful implementation that we argue is outside the scope of the present study. We additionally note that some of the processes mentioned by the reviewer (glaciation and refugial dynamics) are too recent to be compatible with a sole mechanism for the tempo of diversification identified here (with shifts beginning approximately 10-15 million years ago; see Fig. R1 just below).

Fig. R1 (also see Fig. S4 in Supporting Information). The age distribution of diversification rate shifts of 17 rosid orders estimated by BAMM. Panel A: Ridge plots of the age distribution of diversification rate shifts for each rosid order; data from orders Geraniales and Vitales were removed from plotting because only 1 shift was detected in each; no shifts were detected for Crossosomatales, Huerteales, Oxalidales, Picramniales, and Zygophyllales in our BAMM analyses. Panel B: Boxplot shows a summary of overall ages of each diversification shift detected across all 17 orders, with time in "Myr" on the y-axis. Generally, the times of the overall diversification rate shifts are coincident with an earlier cooling period at the Eocene-Oligocene Glacial Maximum (~34 Myr; Panel A). Also, as shown in Fig. S3 in Supporting Information, diversification rates from most rosid orders show abrupt rate increases from ~15 Myr (around the Middle Miocene Climatic Optimum) to the present .

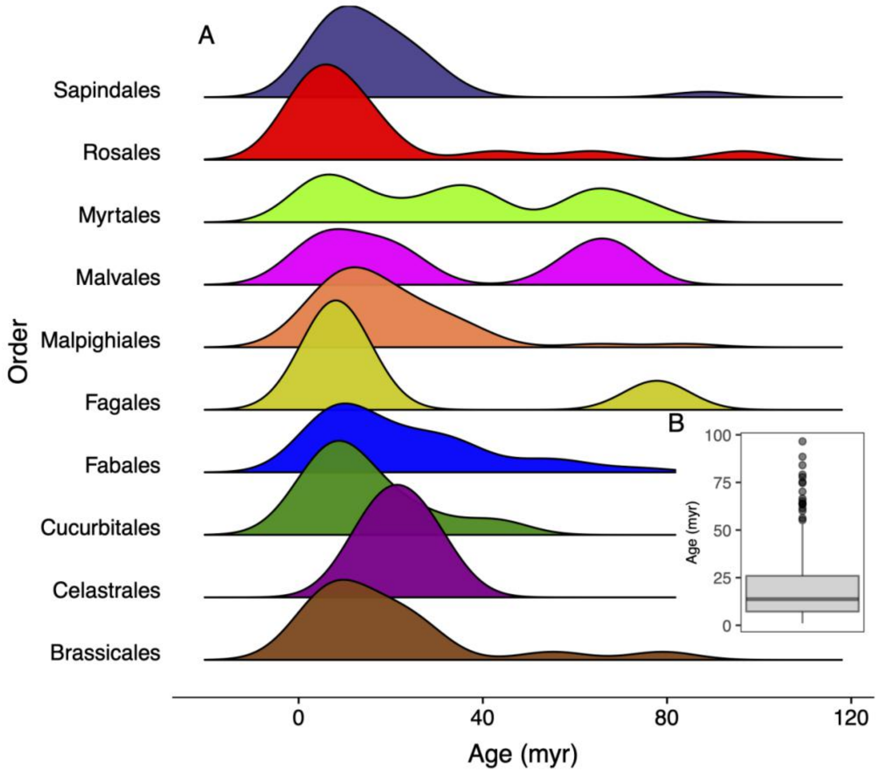

Reviewer #2 (Remarks to the Author):

Understanding large-scale spatiotemporal variations of macroevolutionary rates is a major goal of evolutionary science and a critical piece of the puzzle for understanding global patterns such as the latitudinal diversity gradient. Although the history of this literature is long, recently available datasets and methods are producing novel perspectives on the drivers of macroevolutionary rates.

This contribution by Sun et al. examining diversification rates vs. temperature and latitude of the largest clade of flowering plants would be a welcome addition to the literature, and should be of general interest. Many hypotheses regarding the mechanisms underlying diversification rates with latitude predict higher speciation rates in areas with high temperatures. In contrast, Sun et al. find there is a negative relationship between temperature and diversification rate, both spatially around the globe with regards to contemporary climate, and historically as the climate has cooled. This paper follows a series of recent studies across taxa that find either no correlation with latitude or high speciation/diversification rates in high latitudes. So, the result is consistent with recent results from other taxa even if it is not predicted a priori by theory. This is exciting and timely.

I have a number of comments to help improve the ms.

1) Line 256: The authors cite 9 papers in the discussion apparently showing increased diversification rates associated with global cooling in plants. First, this seems like it might be mentioned in the introduction, since it is background context for the study. Second, if this pattern is widely documented, perhaps a more clear explanation of the novelty of the current analysis would be warranted as well.

> *Regarding the first point concerning literature review, we have reviewed these citations and brought some of the citations forward for clarity in the introduction as requested. In the process, we found that some citations were holdovers from earlier versions of the manuscript. We have removed those references to leave only those papers germane to the point, along with additional citations.*
>
> *On the second point (novelty of the results), our study has several novel points that build substantially on previous literature and identify new patterns. First, we report on non-tropical drivers of diversification. Ours is one of the few studies to identify non-tropical areas as loci of recent diversification, and it does so using novel methodological approaches. Our framing is more direct than previous cited literature in quantifying tropical vs. out-of-tropics diversification rates, with the first attempt at explicit consideration of climatic and geographic facets of "tropicality," and with stronger sampling to enable*

this comparison than some previous studies focused on temperate clades. Our patterns are also in conflict with several key hypotheses in the literature, suggesting a need for studies across the tree of life to identify general patterns. For instance, a recent study in ants found no correlation of latitude with diversification rates (Economo et al. 2018 Nature Communications*); a study in fish (Rabosky et al. 2018* Nature*) finds highest diversification in polar regions. Neither of these studies assesses climatic definitions of tropicality. Both implicitly assume a geographic conception of tropicality, yet we find a tighter correlation with climate than with geography. Hence, the overlap of our results with previous literature is less than it seems based on the flurry of recent papers, a point we have tried to drive home in the discussion section more clearly.*

Second, our dataset, as measured in terms of sampled species, is among the largest attempts so far to understand global diversification processes, and the largest we are aware of using only molecular data as opposed to fill-ins with taxonomic data (e.g., compare Rabosky et al. 2018 Nature*). This broad scope provides a means to use replication across multiple orders of plants to examine concordance, which we believe is critical for getting closer to processes operating across multiple independent cases. The difficulty of handling large datasets has been a roadblock to assembling evolutionary patterns in large, globally distributed clades and uncovering generalizable mechanisms of macroevolution.*

The nuances of the results in our manuscript, in combination with contrasting and similar patterns in a series of related papers in the last two years, highlight these gaps in knowledge, the timeliness of work of this type, and an emerging need for synthesis. Addition of new visualizations and analyses and improvement in clarity of the writing both help to more effectively bring these key messages about novelty and impact of the work home in the revised manuscript.

2) The opening sentences relate to the rise of angiosperms in the Cretaceous and explanations for their diversity. This is perhaps a stylistic issue but I feel this opener was discordant from the rest of the paper, which isn't about the rise of the angiosperms and overall success but rather about geographic and environmental correlates of the their (especially more recent than Cretaceous) diversification patterns.

We have examined and reworded this section. While displaying more nuance of the age of angiosperm diversification, we retain some emphasis on the "abominable mystery", which has been a central challenge specific to the literature of angiosperm evolution with a long and distinguished history approaching two centuries. This framing will facilitate broad interest in the

plant community, a potentially significant portion of the anticipated readership of our work (although this work should have broad appeal outside the plant community as well). We now address the framing issues brought up by this reviewer immediately after this modified first broad sentence.

3) [note updated below] I am concerned about the lack of clade-specific missing data correction, if that is correct (I'm not sure). Is there a reason that clades were not corrected for missing data on an individual basis? Typically, within a large taxonomic group, some clades are better studied than others and have more molecular data available, and this can vary dramatically depending on which clades had been the subject of large projects.

-update, in the results I see that this correction was applied specifically tailored to each clade, so my point doesn't apply. I'm leaving this comment so that perhaps the methods can describe this more clearly, by noting that it was clade specific and not for rosids as a whole in the main paper.

We have examined the main text methods and made this point clear (now in a separate paragraph), so readers do not need to consult supplementals.

4) Line 289: This paper has many analyses of diversification rate, not just speciation rate, so this statement is a bit strange.

The original intent of the statement was to point out that our comparison of models has primarily focused on extracting the speciation rates as these are more reliable to estimate from extant-only phylogenies, whether this was done using pure-birth or birth-death models. It was not intended to refer to all of our analyses as being implemented under a pure-birth framework. We have examined and updated the phrasing to show we implement methods with and without extinction. We have also taken great care to be precise regarding our use of diversification versus speciation, to assure that there can be no confusion regarding what we are measuring.

5) Passage line 242-248: I am having trouble following this argument or how it relates to the results.

We have examined this statement and edited for clarity and conciseness.

6) One important thing about the results is that statistical correlations with temperature and latitude don't seem to hold within order, but hold across order (or pooled across data), if I interpret table 1 correctly. This is despite the fact that the orders should be large enough to have statistical power to find these correlations. The authors don't seem to explain or interpret this. This seems an important part of the conclusions, and I am not myself sure what it means.

The comment refers specifically to statistical significance as the estimated negative sign of the correlation does hold across almost all orders. Hence, we respond here to the idea that trees are "large enough" for statistical power. Early literature on state-dependent methods such as BiSSE (Davis et al. 2013; https://doi.org/10.1186/1471-2148-13-38) indeed showed promising results in terms of statistical power as long as trees were relatively large (>300 tips, comparable to many of our ordinal phylogenies), and the BiSSE statistical paper has been extensively cited. However, more recent literature (cited below) demonstrates serious Type 1 error and other false-positive issues when applied to empirical data where the trivial null of constant diversification is essentially always violated; recent literature also shows issues with evolutionary pseudoreplication (see in particular the somewhat shocking result that false positives are 100% under some simulated scenarios; Rabosky and Goldberg 2015; https://doi.org/10.1093/sysbio/syu131). These results have rendered earlier work unreliable for testing statistical power. Hence, the consensus in the literature at the moment is that BiSSE at least has artificially high "statistical power" due to these problems, and that tree size interacts strongly with the specific evolutionary scenario and exact model setup (Gamisch 2016; https://doi.org/10.4137/EBO.S39732; Rabosky and Goldberg 2017; https://doi.org/10.1111/evo.13227; perhaps still best summarized in the discussion section on tree size in Rabosky and Huang 2016; https://doi.org/10.1093/sysbio/syv066). Thus, BiSSE should not be used without either simulations or (as we implement now and increasingly the standard) comparison of BiSSE with models such as HiSSE, FiSSE, and STRAPP that account for these issues. We are not aware of encouraging results out there using these newer methods with small trees; the papers cited above all explicitly identify and sometimes extensively discuss low power as an issue. Challenges of phylogenetic, divergence-timing, and niche estimation error are further issues little addressed in simulation literature to date. As a result of this refresher of our literature search, we have adjusted the methods section with the most up-to-date work showing that power is indeed a potential issue with relatively large datasets using the least problematic methods (Rabosky and Goldberg 2017; https://doi.org/10.1111/evo.13227; (Grundler and Rabosky 2020, https://doi.org/10.1101/2020.01.07.897777). Additionally, we also highlight the broader issue that we are often at the limits of extracting information from what is essentially equivalent to a model of molecular data fitting a single nucleotide position (that is, binary-state approaches are similar to and in some instances generalized cases of molecular evolution models such as the covarion model; see Grundler and Rabosky 2020 and citations therein).

To comment specifically on whether particular orders have sufficient sampling: 7/17 orders have more than 1,000 sampled species, while 4/17 orders have fewer than 100 (two of these, Picramniales and Huerteales, are dealt with

elsewhere in this letter). The most species-rich orders in our dataset are Fabales (5,678 sampled species) and Malpighiales (3,868 species). These two do tend to recover the lowest p-values. For instance, see Table S3 and Table S4 p-values prior to correction. In these cases, these largest orders have the lowest p-values, but in several instances were only significant prior to multiple-comparison controls (especially for Malpighiales). This suggests to us that we are at the margin of being able to detect trait-associated diversification despite the size of the dataset.

7) Line 338: "All analyses were run in parallel across these subtrees." This is a bit unclear, does this mean BAMM and all div rate analyses were inferred totally independently? I have no real objection to doing it this way, but is there a reason why you did this instead of analyzing it all at once?

Update- I found it in the supplement that this was done for computational efficiency reasons. I think since it is an important decision in how the models were run, you could add the phrase "For computational reasons, it is difficult to run diversification rate analyses on the whole rosid clade, thus we analyzed each order independently" or something to that effect.

We have added the suggested phrase to the main text methods.

8) It is also unclear how STRAPP was applied to the whole dataset if runs were independent, was it combining the independent clade-wise runs somehow? That would seem problematic since one would not know whether different parts of the tree run independently would have the same macroevolutionary process (important in the permutation test)

We did not apply STRAPP to the whole tree for computational reasons, and we also further clarified this in the main text and supporting information so that the tables will be self-evident.

9) Line 379: Should the title of this section be something more descriptive, along the lines of "Diversification rate inference and statistical analysis"

We have implemented this change.

10) Table 1. Many of the adjusted p values are identical across taxa. Is this explainable somehow due to how the mult-comp corrections work?

We re-checked the p-values here. There is no problem with the calculation of the adjusted p-values. Regarding "identical adjusted p values" across some rosid clades for a trait, at the smallest family-wise significance level (alpha; below the conventional 0.05 level), those p-values from some clades have the

same the family-wise error rate among the rejected hypotheses. Hence, identical adjusted p-values simply means they have the same statistical power for rejecting/accepting the null hypothesis, under the Hochberg method (Hochberg, 1988) as implemented in the "p.adjust" function in R.

11) Table 1: I also feel somehow you should put the sign of the result of the FiSSE analysis on the table, so people can clearly interpret what biological finding the p values refer to.

We divided Table 1 into two, with Table 2 indicating detailed values from the FiSSE analysis as requested.

12) Line 105: A little more background on the macroscale patterns in rosid diversity would be helpful in the introduction. The authors say "community-level" species richness but don't directly address how richness varies for example, by latitudinal band. Are there more rosid species present globally between 0 to 10 degrees than between 30 to 40 degrees? This seems important information for the reader to interpret the results.

For the first point, we have added clarity to introductory material on the rosids. However, we have primarily chosen to address this comment through additional figures, analysis, and interpretation placed in the Discussion section as the characterization of rosid latitudinal diversity is primarily the novel result of this paper with little available in the previous literature to cite. For the second point on latitudinal patterns, this information was present but dispersed in the first draft and not necessarily presented in a straightforward way. For instance, to respond to the specific query, based on a summary from the rosid distribution data ("rosid_18269_species_occ.csv" on GitHub: https://github.com/Cactusolo/rosid_NCOMMS-19-37964-T/tree/master/Datasets/Species_Distribution_Data), we found that 5660 species occur in the 0-10-degree latitude zone, and 8255 species occur in the 30-40-degree latitude zone. Hence, community species richness is higher in the tropics (see Fig. S1 in Supporting Information) but latitudinal bands show more parity among latitudinal zones. Of course, strong differences in total land area by latitude is a significant confounding factor in these calculations that is not accounted for here.
We therefore have calculated new site statistics on equal area grids (approximately 322km across) that overcome this problem. In the first draft we only presented species richness in the supplementals, and now we added a main-text figure (Fig. 2) based on our new spatial statistical analysis showing tip rates (BAMM and DR), and we also include an age metric of the species in the community. Species tip age is defined in the new analyses as the age of the closest node; this was done for each species in the community with a community median reported for each grid cell (see Fig. S4 in Supporting

Information for density distributions and a boxplot). These figures demonstrate a clear age and diversification rate disparity across the tropical and non-tropical areas of the globe (Figs. S3-S4), as we now note in several areas of the manuscript in support of the points presented in our earlier draft.

13) Line 387: How was BAMM used to examine correlations between historical temperature and diversification rates statistically? The methods refer to supplementary section M3 but in there it just describes how BAMM was run. STRAPP can be used for relating tip rates with traits, and BAMM can be used to plot rates vs time, but it is unclear how BAMM was used to asses historical correlations between temperature or time and diversification rates as RPANDA does (or was it just compared visually?).

These results can be found in Fig. S2 in current draft. This is not a STRAPP method detail; we essentially extracted a rate-through-time curve (100 time unit slices; net diversification rate) from the BAMM rate-through-time matrix. We then performed interpolation to associate the data to corresponding historical temperature data via the 5 point method. We have explained the approach more thoroughly in the main text Methods; also see R script in GitHub (https://github.com/Cactusolo/rosid_NCOMMS-19-37964-T/blob/master/Scripts/misc/Fig_S2.R).

14) Are there other studies that found the Miocene cooling (other than Folk 2019) to be an important period for buildup of extant temperate diversity, or is this the first one? The Oligocene cooling is most commonly discussed as a prediction of the tropical conservatism hypothesis, I believe.

We thank the reviewer for this suggestion, and yes there are papers supporting this hypothesis broadly across plant exemplars (e.g., Hypericum*, Nürk et al., 2015). We have studied the literature and cite more papers relevant to this subject. We note that a Miocene date is compatible with the idea, given that the Earth was still primarily tropical in the early Miocene and paleobotanical evidence does not reveal an expansion of non-tropical plant communities until after this date.*

15) The phylogenetic and geographic data are necessarily a bit fragmentary relative to the size of the group, as acknowledged by the authors. This is unavoidable at this stage and I think the authors do a good job overall of making use of available resources.

We thank the reviewer for this evaluation and hope the revisions make the point even stronger.

The phylogeny in particular was constructed by stretching available sequence data, with many species added to the tree with only a single locus, and (I think) all analyses depend on a single topology and dating of the tree using penalized likelihood, that does not represent uncertainty in topology or clade ages or pass that forward to the analysis. Using a single topology and dating has previously been a point of criticism in large megaphylogeny projects (e.g. Title et al. 2016).

The authors largely discuss the tree reconstruction in their companion paper, which I have also read. They also do quality control by comparing topology and clade ages to other studies, which seems to me to be a good approach to cross-validation. I think it would help the current ms to add a few sentences to the methods describing quality control and robustness of the tree.

We added more sentences to the methods describing quality control and robustness of the tree.

16) Line 290: Citing Folk et al 2019 is probably not appropriate here, as this idea has been around for much longer.

We added in more literature and removed "Folk et al. 2019".

17) One limitation of the study is the authors did not analyzes ages of clades inside and outside the tropics, such as for example e.g. assess "diversification time" to complement the diversification rate analysis (e.g. Kerkhoff 2014 PNAS did something like this for plants). It would be interesting to know if all the temperate clades are old and only had recently accelerated diversification (seems fro fig 1 this could be the case), or whether many transitions out of the tropics occurred recently. In lieu of new analyses, some discussion of other papers/citation could address this issue.

We have performed and added a series of new analyses (Fig. 2 in main text, and Fig. S4 in Supporting Information). We find that the median date of diversification rate shifts is circa 13.71 Myr (see Fig. R1B in the Reviewer #1 section of this letter), consistent with the first draft of the manuscript. Some of the clades composed mostly of non-tropical species contain subclades that are quite old (74.7 Myr-96.5 Myr; see Fig. R1A, and Fig. S4 in Supporting Information: Rosales and Fagales), but in general, tropical groups are somewhat older under both climatic and geographic definitions (T-test: p-value $< 2.2e-16$ for both datasets). The pattern is more obvious when plotting community ages (see below).

The following box plot (Fig. R2) shows that, in general, divergence events are still older in tropical clades than in non-tropical clades, although with overlap. A map of clade ages per grid cell below shows that, as expected, older communities are primarily in the tropics. There are outliers in the poles and

oceanic islands; these are often very small communities so statistical sampling error may bias the median estimate.

Fig. R2 (also see Fig. 2 in main text). A. Boxplot for tip ages estimated for both tropical and non-tropical rosid lineages defined by climatic and geographic definitions. Species tip age is estimated from the age of the closest node for any given tip in the dated rosid tree; tropical lineages are older than non-tropical ones (T-test: p-value < 2.2e-16 for both datasets). B. Global distribution of median species age for all rosid species sampled in this study, showing the overall age pattern for tropical and non-tropical species. The median species tip age (mentioned in A) is the median age value from those species nested in each grid cell of approximately 322 km width.

A.

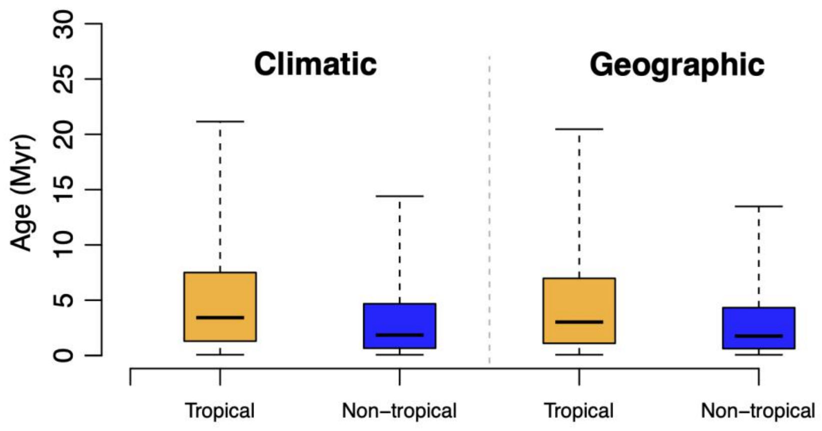

B.

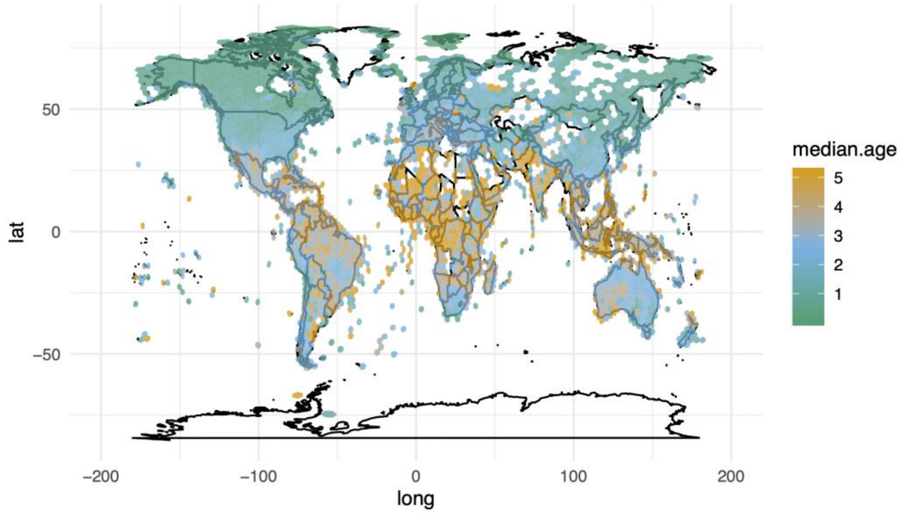

Fig. R3 (also see Fig. 2 in main text). A. Global distribution of tip rates estimated by BAMM for all rosid species sampled in this study showing the overall rate pattern for tropical and non-tropical species. B. Global distribution of tip rate estimated by DR statistic (Method S2) for all rosid species sampled in this study showing the overall rate pattern for tropical and non-tropical species.

A.

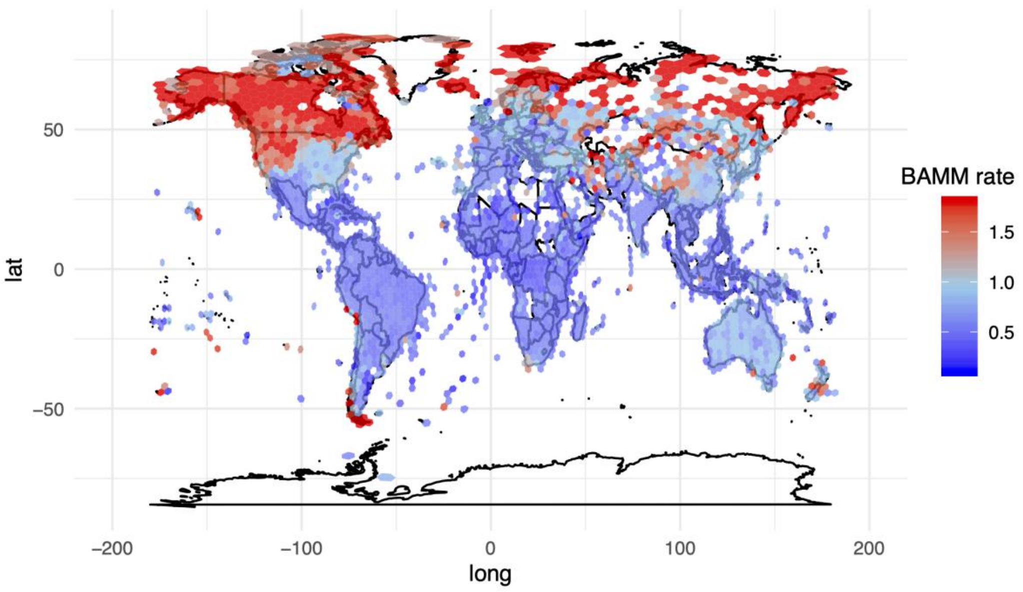

B.

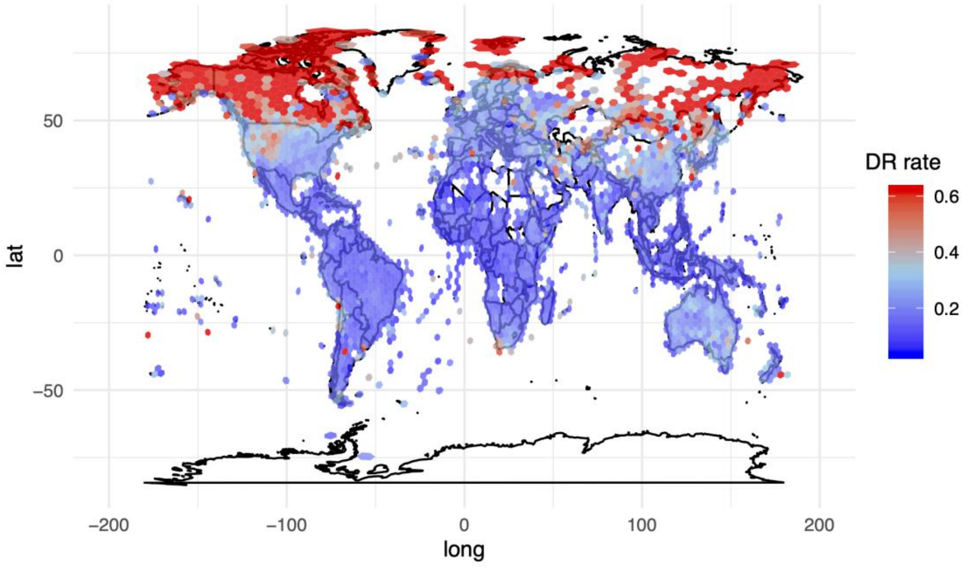

Reviewer #3 (Remarks to the Author):

Sun et al. investigate the diversification dynamics of a large angiosperm clade, the rosids, in relation to temperature niche and tropical/temperate distributions. They find that diversification is highest in low temperatures and in temperate/arctic places, but only when testing the models for the whole rosid clade, in their subclade (order) approach they only find significance for one or two lineages, depending on the test (see Table 1). If true, this result is interesting, quite novel for angiosperms (especially for such a large clade which also includes very important tropical elements) and will raise awareness and discussion in the fields of plant evolution and ecology.

I applaud their efforts of collecting these large datasets and cleaning millions of occurrence records, in addition to using a previously published phylogenetic tree of the rosids. However, I have a couple of major issues which make me unsure about the reliability of the results and main conclusion. Of course, understanding past dynamics will always be challenging because we base it on correlation and inferences of past speciation/extinction rates, but in my opinion it would be essential to base these correlative tests on a solid hypothesis which also finds support in the experimental or physiological literature, for example.

This directly relates to my first major concern. Such a hypothesis is missing. Many studies have tested the link between diversification and temperature or tropical/temperate across many taxonomic groups and found contrasting results (outlined nicely in the introduction). However, not many studies explicitly present the diversification mechanism for the link between temperature or tropical/temperate niche and diversification dynamics. One hypothesis is that high temperatures would lead to high mutation rates and this may facilitate speciation. But when hypothesising that cold temperatures lead to high diversification, a solid hypothesis is missing. In the discussion the authors briefly touch on a possible mechanism by suggesting that Late Miocene opening up of temperate niches provided ecological opportunities for temperate radiations. This is possible, and the RPanda and BAMM results (Fig. 2) could provide support for this idea, but I would prefer to see it better integrated within the manuscript. It also means that temperature by itself is not a diversification driver, it's the opportunity of empty niches, which happened to be in temperate regions from the Late Miocene onwards. So to fully test this hypothesis, the manuscript would need rewording and a more sophisticated way to identify empty niches or ecological opportunities through time. The issue with this idea is that these temperate opportunities also led to reduction in tropical biome areas and opportunities, which may have simultaneously led to extinctions in tropical rosids. So it would be a challenge to identify whether the Late Miocene diversification increase is an artefact of not being able to see the extinct tropical lineages in the phylogeny, or whether it is a true signal. This could be done with simulations in which one simulates trees under constant speciation and extinction rates for both temperate and tropical lineages, and then selectively drops tropical lineages during the

Miocene/Pliocene from the tree, without changing speciation parameters in the simulation, and then repeating the analyses. A script to do this is provided by this study: https://royalsocietypublishing.org/doi/full/10.1098/rspb.2018.0882?url_ver=Z39.88-2003&rfr_id=ori:rid:crossref.org&rfr_dat=cr_pub=pubmed.

We certainly agree about the need to cover more about potential mechanisms in the frame of current hypotheses, and we added (and highlighted) those hypotheses in the introduction. However, we don't fully agree with the reviewer on the hypothesis that warmer conditions should necessarily lead to higher speciation rates via higher mutation rates. There is little empirical evidence for that hypothesis, and thus it is potentially something of a straw man here (and likely in other studies as well). Quoting Schluter (2016), "To date, little compelling evidence connects temperature to speciation or diversification rates via higher rates of mutation (Davies et al. 2004; Evans and Gaston 2005; Dowle et al. 2013; Bromham et al. 2015)". In contrast, there has been increasing evidence that ecological opportunity is a catalyst for increased diversification (Schluter 2016). However, key questions about density-dependence of this process over time and space are still controversial, and in particular it is not yet well understood how habitat space fills and how this relates to niche occupancy, making models of this process non-trivial. Previous work in our group has made the case that in some instances one might expect continued habitat availability. It may be possible to better understand mechanisms via assessing coincident trait evolution (see recent work by Folk et al. [2019]). Testing the role of specific traits involves assembling detailed phenotypic data, a significant undertaking for this enormous group. In sum, the reviewer's assumption of a strict relationship between empty habitats and diversification in terms of setting up a simulation framework may be too simplistic. Additionally, while we fully support acknowledging the role of extinction, which is hard to estimate from empirical extant-only phylogenies, we argue that based on the geological record, the scenario of expanded temperate niche with no speciation of temperate species is extremely unlikely. Paleobotanical reconstructions (Pound et al., 2011) show that there were very few temperate plant communities comparable to modern assemblages before the mid-Miocene, and these communities were largely in a spatially highly restricted area, i.e., restricted to >60 degrees latitude. Creating enough standing temperate diversity to recreate modern-day diversity patterns seems near-impossible under ancient climate scenarios before the Miocene. We also note that our new analyses (Fig. R1 in this letter, and also see Fig. S4 in Supporting Information) show that most diversification rate shifts occurred during the Miocene/Pliocene time period, and this timeframe is at least temporally coincident with establishment of new temperate habitats, although as noted before, we don't know how quickly this happened or if habitats became saturated. We foresee value in a more

rigorous set of simulations related to these key questions of rates of suitable habit change promoting speciation, and presumably accelerated extinction rates as other, tropical habitats shrank, but to conduct such simulations effectively would be a major undertaking in itself and is beyond the scope of this contribution.

Aside from the arguments here regarding the timing of significant biome-level changes, we note that we have done empirical "simulations" (dropping experiments essentially identical other than being based on the true diversification process rather than guessing at parameters) that are very similar to what is described here. The sampling space we are exploring is focused on understanding how much skew there is towards over-representing non-tropical and tropical species, as a key aim was to test whether taxonomic bias drove the results. This suggestion, aside from how to generate the trees, is simply a sampling experiment in the other direction. For a variety of reasons, ranging from concern about realism of processes to already knowing that the results are robust to a surprisingly wide array of empirical sampling parameters, we argue that a further simulation study is not in the scope of the current paper.

In addition, it is unclear from the BAMM plots (Fig. 2) whether both tropical and temperate lineages increased in diversification during the Miocene/Pliocene. It would be nice to distinguish between those in the plots, because alternatively, all rosids increased diversification during this time due to other, non-measured, time-dependent factors.

We have performed new analyses and updated what was Fig. 2 (now Fig. S3 in light of new main text figures) with curves from both tropical and non-tropical lineages shown respectively. Most of the orders that have a preponderance of non-tropical lineages show higher net diversification rates and stronger curve increases than tropical lineages (also see new Fig. 2 in main text).

My second major concern relates to the use of parametric and semi-parametric tests, rather than using more process-based models such as the BiSSE-type. The used tests use the tip speciation rates to test for a correlation with for example present-day average temperature, but this does not actually link the process of speciation to these characteristics (temperature, temperate/tropical). BiSSE methods have been criticised, but several solutions have been proposed (e.g. including hidden states, as well as using simulations). It would illustrate much better whether the different rosid clades consistently through time show higher speciation under lower temperatures. With such a large clade you would also have the statistical power to test whether rates indeed change during the Miocene/Pliocene, as compared to the earlier Cenozoic (i.e.

with time-dependent BiSSE models).

We have incorporated BiSSE/HiSSE models (see Method S3 and Supporting Information Table S4). We find that hidden state models are supported and hence have similar evidence for tropicality-dependent diversification. Interestingly, this new work supports not only a hidden state, but a reversal in the direction of the diversification relationship with tropicality dependent on the hidden state. In combination with everything else we show, this new analysis supports tropicality-dependent diversification, but note that this is also likely driven by other factors (never a surprise over >100 My of evolutionary history) that we have not been able to characterize.

Another concern is that results remain largely not significant, unless the whole rosid clade is considered. Why do subclades not show consistent significant patterns as well – could it be that it is only found for the rosids as a whole because such a large clade with so many tips would always show significance in a (semi-)parametric test? And why do the orders separately in most cases not show any significance?

Each order differs in species richness; hence, statistical power will vary from order to order. For example, Huerteales is a small clade, with only 7 of 30 species (based on OpenTree taxonomy) sampled, 2 of which are tropical and 5 temperate. Several other orders are also small to very small and would not be expected to have high statistical power.

On the other hand, some large clades indeed show non-significant results. It is true that the magnitude of the difference is often high enough that we should have reasonable statistical power for reasonably large trees (e.g., Fig. 1 of Rabosky and Goldberg 2017). As cited above (in response to Reviewer 1), we have returned to the literature. We find that taxon number interacts substantially with model choice and method (e.g., again Fig. 1 of Rabosky and Goldberg 2017). We also believe it could interact with error in characterizing the niche (possible with automated cleaning as implemented here) and phylogenetic error (uncharacterized as far as we are aware but very likely in this case). Loss of power from multiple comparisons is also an important source: for instance, in Table 2 of the main text, only 1 of 5 orders that were initially significant survives the Hochberg correction. Another very simple explanation for the semi-parametric tests is that by breaking down the phylogeny into orders, we are artificially truncating the number of replicated evolutionary events that these methods try to control for. Finally, we may also be introducing sampling imbalances within orders between the two states that are more extreme than the entire dataset.

Minor concerns:

-Tropical areas also include high-alpine, low temperature mountains, which have been often associated with high speciation rates due to ecological opportunities with mountain uplift. It would be good to mention this, or perhaps it could also explain some of the differences found when taking average mean temperature vs. tropical/temperate classifications.

We have addressed this at Line 184-186. We are very aware of this issue, and this was the (admittedly subtle) reasoning behind the use of the Köppen-Geiger tropicality dataset of the first draft of this paper. Our revision more explicitly states our belief that Köppen-Geiger tropicality appropriately captures the difference.

-In addition to taking the mean temperature for a species, would it make sense to take a measure of temperature seasonality? If indeed Miocene climate changes and ecological opportunities were the driver of speciation, then it may be more seasonality rather than average temperature underlying speciation (e.g. though processes of reproductive isolation and allopatric speciation).

Temperature seasonality can be very highly correlated with non-tropical climates, as are many other temperature-based predictors one could use, and hence we have chosen to respond by highlighting that we may be identifying a spectrum of effects of non-tropical environments. In part due to new results, we are careful not to claim that non-tropicality is the only "key innovation" at play here.

-Assignment of species to tropical/temperature was based on the average occurrence or dominant occurrence in one of these areas – how many species have occurrences ranging in both temperate/tropical? Would it be possible to identify those species as wide range in for example a GeoSSE diversification approach? Because it seems those are not physiologically/biologically limited to tropical or temperate biomes and this would be interesting to evaluate (rather than ignore as currently done) as well.

We have re-checked our tropicality traits datasets under both climatic and geographic definitions (Tropical "1", Nontropical "0"; even if for some species records, the mode is "1", and there is only one record showing "0", we calculate it as occurred in both regions). Focusing here on the geographic criteria, we find that 27% of the species have at least one point occurrence in both non-tropical and tropical areas, but the majority (73%) are found only within non-tropical or tropical areas. Moreover, we also have conducted BiSSE & HiSSE analyses which relate to this question (see Table S4 in

Supporting Information). We were concerned about a more elaborate GeoSSE model because we have only two states, and we do not allow them to be both non-tropical and tropical in the present as it would be hard to set an objective limit for non-tropical and tropical distribution. Under these restrictions for extant taxa, the HiSSE approach seems reasonable.

-The order of diversification methods used is not consistent between the results and the methods, this is confusing. The results section needs a sentence or two to introduce the method used, because it's currently a sum-up of p-values and not clear to the reader what was tested exactly and how.

We have reordered the methods to follow results as requested, primarily organized along the lines of the tested relationships (contemporary temperature/tropicality first, then historical temperature).

We also added a brief summary at the beginning of the diversification results sections of each method. Due to the large number of methods used, in the interest of brevity (as requested above as well), we have also expanded on some of the text in the methods instead.

-RPanda results support the "means temperature (x) dependent birth-death model with constant speciation and extinction rates" – can you explain in a bit more words what this biologically means? Also, Table S6 shows very extreme speciation and extinction rate values for certain clades (e.g. Picramniales) suggesting that something went wrong when fitting the model? Do results suggest that speciation rates are higher and extinction rates lower with higher temperatures? Because that would conflict with your conclusions, but it's a bit unclear how to interpret these values, so please clarify. Also, in the discussion you mention that extinction rates were not considered, but this seems not the case for this method?

Firstly, we have maintained our focus in the main text summaries on the main choice between environmentally and time-dependent diversification. However, we have added an explanation of the RPANDA temperature-dependent birth-death model with constant speciation and extinction rates in the discussion. Briefly, the results support temperature-dependence, with a proportional response of both speciation and extinction to temperature. The sign of the parameters indicates the expected negative relationship, obviously critical to our hypotheses. The exact shape of this relationship is not entirely germane, but we have summarized this as an inverse proportional response to temperature.

*Second, regarding the observation that "*Table S6 shows very extreme speciation and extinction rate values for certain clades*", we argue there are two main reasons: 1) Picramniales only have 5 taxa sampled in the tree, with*

*56 species in total based on OpenTree taxonomy. Four of the 5 sampled species are tropical species, and there is only one temperate species. Hence, there probably is not enough signal to meaningfully estimate the parameters. Running state-dependent binary analyses is an impossibility as well. While we show these results for consistency, almost certainly these datasets are not robust for the smallest orders. We have added a note to this effect in the results. The RPANDA analyses did generate extinction rates since the chosen model was a temperature-dependent birth-death model with constant speciation and extinction rates, but we did not use extinction rates for downstream analyses (see above). Hence, we revised "λ" and "μ" columns in the 1st draft as a new "λ-μ" column in the Table S6 in current manuscript draft. Showing just net diversification, as in the new draft, will clarify the confusing appearance of those values. 2), Some of the "*extreme values*" (e.g., negative λ and μ) may also arise in RPANDA from the way that likelihood optimization is implemented; these values should be interpreted as their absolute values (see RPANDA developer's discussion: https://github.com/hmorlon/PANDA/issues/11).*

-The purpose of the sensitivity analyses is not entirely clear – you repeat the STRAPP analyses with different sampling schemes, but (almost) none of the STRAPP analyses for the orders was significant in the empirical results, so how does the sensitivity analyses contribute to this?

Renske Onstein

As noted, running analyses on the entire dataset was an impossibility. At least this analysis is able to confirm that with gradually removing the non-tropical lineages, the effect on the parameter estimates is minimal, especially for the Köppen-Geiger tropicality dataset. That is, in almost all cases, rates estimated from non-tropical lineages are higher than tropical species in all taxon-drop treatments; some are slightly closer or equal, but never lower than rates estimated for tropical species (Table S5 in Supporting Information). That the trend remains unperturbed we interpret as evidence for robustness to incomplete sampling.

Reviewers' comments:

Reviewer #1 (Remarks to the Author):

I think the authors have done a good job in their revision. As a minor point they say in their response that there is no evidence that increased rate of speciation/diversification in plants can be linked to increased rate of mutation (e.g. via increased temperature). This is not exactly true and the authors might want to look at/cite Barraclough & Savolainen, 2007, Evolution 55: 677-683 as an example of such correlations

Reviewer #2 (Remarks to the Author):

This is a second review of Sun et al. "Recent, accelerated diversification in rosids occurred outside the tropics". Overall, I think the authors have done a very thorough job responding to the comments. I found the new analyses and figures greatly improve the robustness and clarity of the results. The authors really do a good job of attacking their central question with a variety of methods, and these all give consistent results which is comforting.

I have one final point:

Since the last review, another paper on this topic has come out showing relatively similar results in plants with a larger tree 60,000 tips (Igea et al., 2020, Ecology Letters). I personally support publication of this new paper as well, both for sake of independent/reproducible science and because it goes further than the previous analysis. The new paper in particular seems to be novel in correlating with temperature rather than geographic tropicality, as well as look at diversification-temperature patterns through time, whereas Igea et al focus on geography and do not look into deep time. However, clearly Igea et al. needs to be cited and at least be discussed in this paper.

Reviewer #3 (Remarks to the Author):

Sun et al. addressed several of my concerns (as well as those from reviewer #1) and the text reads well. Many analytical steps are a lot clearer now. The additional HiSSE analyses also make the ms a lot stronger. However, some of my (as well as reviewer #1) main concerns remain:

1. In their response, the authors indicate that they have further emphasized the potential mechanism for higher diversification in temperate/low temperature places compared with tropical places. As they do not indicate the exact changes in the ms, I re-read the whole ms and found this implementation very limited. Yes, they do mention the ecological opportunity scenario (which was already there in the previous version), and also indicate that results may deviate because of high alpine radiations in tropical zones. However, a more mechanistic interpretation is missing. I want to emphasize that even though lots of studies look for this correlation between diversification and temperature or tropical/temperate, and this study therefore can be added to the list, I am not alone in my opinion that this is a correlative exercise without much biology behind it. The only thing I ask for is to think more carefully about the biological mechanisms underlying these patterns, and support this with the literature. I gave some examples in my previous review (those were just suggestions, feel free to ignore as you did) – another example would be that of massive variation in diversity within the tropics (e.g. the overlooked seasonally-dry tropical forests) which strongly deviate from a LDG pattern, and makes this tropical/temperature comparison just a bit superficial. I would therefore like to see a more careful discussion of this, the possible mechanisms underlying some of your patterns, the potential deviations within the rosid clade that would possibly follow this (e.g. diversification variation within particular temperate/tropical biomes/regions). Simple mean temperature really does not tell us much.

2. Did you mention somewhere in the text how many species had both tropical and non-tropical occurrence records (27%), or only in the response to me? I still think this uncertainty should be considered rather than ignored – at least in a sensitivity analysis. Your argument that you are concerned about a hidden-state GeoSSE model because you do not allow taxa to be both non-tropical and tropical is exactly the problem – you should allow them to be both tropical and non-tropical, and this is possible in GeoSSE (three states: tropical, non-tropical and tropical/non-tropical).

3. The HiSSE hidden trait – any ideas what kind of hidden traits may relate to temperature and diversification and could be mentioned/discussed?

Did you take topological and branch-length uncertainty into account in your analyses (e.g. run analyses over a set of trees)? This would be essential and it's unclear from the text whether this has been done.

Minor comments:

L250 write out what DR and BAMM stand for
L264 Indicate the value of lambda – there is quite some discussion in the literature whether phylogenetic signal actually reflects a process such as phylogenetic niche conservatism. It would be good to mention this here, in the discussion or in the methods, and be careful with statements such as 'phylogenetic niche conservatism' (clearly, rosids made many transitions to temperate regions).
L265 But STRAPP does not test for phylogenetic niche conservatism, or?
L274/275 Again, indicate the value for lambda here
L340 Two times 'significantly' – remove one
L346 Indicate t-test
L359 This sounds like topicality – species in the tropics – is associated with high diversification, but that's not what you mean right? Maybe rephrase.
L364 An unobserved state, or an unobserved trait?
L482-483 Was it consistent with BAMM rate shifts, or did you actually correlate BAMM rates in the tree (so not tip-rates) with paleotemperature? I see in methods that this is done using regression models. There is of course the issue that a correlation is found because both are correlated with time (temporal autocorrelation). This should be acknowledged at least (or corrected for). I would also be happy if you just indicate that the patterns are congruent, and there is thus a consistent relationship with the other tests performed, rather than doing a regression (otherwise people would repeat this approach and I do not think it's valid).

Is there space for Fig. S3 in the main text? I think it's a strong figure.

Renske Onstein

Reviewers' comments:

Reviewer #1 (Remarks to the Author):

I think the authors have done a good job in their revision. As a minor point they say in their response that there is no evidence that increased rate of speciation/diversification in plants can be linked to increased rate of mutation (e.g. via increased temperature). This is not exactly true and the authors might want to look at/cite Barraclough & Savolainen, 2007, Evolution 55: 677-683 as an example of such correlations

We thank this reviewer for the suggestion and reference. The Barraclough & Savolainen paper is more focused on species numbers, morphological traits, and the rate of molecular evolution rather than directly linking to temperature. That paper finds a link between neutral evolution rate, but not morphological rates, and species number, thus discounting adaptive explanations for such patterns. However, that paper does not provide direct evidence for the question at hand, which relates to the issue of temperature as a driver of such patterns. Further, it is equivocal here at best since temperature should likely impact morphological and physiological trait character suites (as noted below in response to Reviewer 3), which were shown to relate to species number. Given the above considerations, we decided to forego including this citation in the main manuscript. We do think that the hypotheses posited in Barraclough & Savolainen (2007) are critical ones, and well worth reconsidering given growth in the data resources and maturing methods since that work was published.

Reviewer #2 (Remarks to the Author):

This is a second review of Sun et al. “Recent, accelerated diversification in rosids occurred outside the tropics”. Overall, I think the authors have done a very thorough job responding to the comments. I found the new analyses and figures greatly improve the robustness and clarity of the results. The authors really do a good job of attacking their central question with a variety of methods, and these all give consistent results which is comforting.

I have one final point:

Since the last review, another paper on this topic has come out showing relatively similar results in plants with a larger tree 60,000 tips (Igea et al., 2020, Ecology Letters). I personally support publication of this new paper as well, both for sake of independent/reproducible science and because it goes further than the previous analysis. The new paper in particular seems to be novel in correlating with temperature rather than geographic tropicality, as well as look at diversification-temperature patterns through time, whereas Igea et al focus on geography and do not look into deep time. However, clearly Igea et al. needs to be cited and at least be discussed in this paper.

We thank this reviewer for an excellent suggestion. We agree with reviewer 2 regarding the scope of that work compared to this one, and the importance of the rigorous and comprehensive approach we have employed here. We have now cited this paper in multiple places in this revision, and discussed its findings in our manuscript, as suggested.

Reviewer #3 (Remarks to the Author):

Sun et al. addressed several of my concerns (as well as those from reviewer #1) and the text reads well. Many analytical steps are a lot clearer now. The additional HiSSE analyses also make the ms a lot stronger. However, some of my (as well as reviewer #1) main concerns remain:

1. In their response, the authors indicate that they have further emphasized the potential mechanism for higher diversification in temperate/low temperature places compared with tropical places. As they do not indicate the exact changes in the ms, I re-read the whole ms and found this implementation very limited. Yes, they do mention the ecological opportunity scenario (which was already there in the previous version), and also indicate that results may deviate because of high alpine radiations in tropical zones. However, a more mechanistic interpretation is missing. I want to emphasize that even though lots of studies look for this correlation between diversification and temperature or tropical/temperate, and this study therefore can be added to the list, I am not alone in my opinion that this is a correlative exercise without much biology behind it. The only thing I ask for is to think more carefully about the biological mechanisms underlying these patterns, and support this with the literature. I gave some examples in my previous review (those were just suggestions, feel free to ignore as you did) – another example would be that of massive variation in diversity within the tropics (e.g. the overlooked seasonally-dry tropical forests) which strongly deviate from a LDG pattern, and makes this tropical/temperature comparison just a bit superficial. I would therefore like to see a more careful discussion of this, the possible mechanisms underlying some of your patterns, the potential deviations within the rosid clade that would possibly follow this (e.g. diversification variation within particular temperate/tropical biomes/regions). Simple mean temperature really does not tell us much.

> *We wish to apologize for any appearances of not considering the issue raised by the reviewer. We do think this is an important consideration, and we feel that the new revision now more directly considers this issue in the required level of detail. Having said that, we were initially concerned about how much we could make a strong mechanistic case, given issues related to lack of temporal resolution of key climatic variables such as seasonality and its change over millennia. We were also concerned about potentially high correlation of key variables brought up previously by this reviewer (e.g., seasonality, but also other predictors) with the temperature predictors we present, which do have a strong temporal record already in the literature. Still, we fundamentally agree with the reviewer and*

have now taken the criticism to heart in terms of rewriting the discussion and drawing on new literature.

We hope that this area of the manuscript (primarily the second-to-last Discussion paragraph) will be found to be substantially improved to account for these criticisms. In particular, we make the case that a next step should be considering physiological adaptation to freezing tolerance in angiosperms and its distribution across angiosperm diversity, as this represents a potentially unifying explanatory mechanism for the turnover of much of the world's flora outside the tropics in recent times. We have now refreshed ourselves on the literature, and drawing on our previous work and the work of others, argue that bursts of diversification may relate to exapting pre-existing cold tolerance traits in response to climate change. This provides a potential mechanism, well supported in the literature, that ties together results in the literature and presents a clear way forward in terms of studies to better validate this mechanism, as we outline towards the end of the Discussion. We finally note that as we gather improved species distribution knowledge at finer scales, it will be valuable to move beyond very coarse assessments of climatic niche and consider areas in the tropics that are more or less seasonal, and related questions about diversification. We add a note to this effect in the main manuscript (quite a few new lines in various places in the Discussion) and end here simply noting the exciting potential and next steps that can be taken following along lines in this work.

2. Did you mention somewhere in the text how many species had both tropical and non-tropical occurrence records (27%), or only in the response to me? I still think this uncertainty should be considered rather than ignored – at least in a sensitivity analysis. Your argument that you are concerned about a hidden-state GeoSSE model because you do not allow taxa to be both non-tropical and tropical is exactly the problem – you should allow them to be both tropical and non-tropical, and this is possible in GeoSSE (three states: tropical, non-tropical and tropical/non-tropical).

Previously we only mentioned this information in the response letter; now this information is incorporated in the main text. We forego a GeoSSE analysis here for three reasons. First, this work already has a particularly extensive set of analyses that form the basis for the paper. Second, we have done a relevant test --- "HiSSE" as the reviewer suggested in the first review round. Third, we will note that in the majority of those 27% percent scored as "both" temperate and tropical, it is only a few records that extend in either temperate areas for a tropical species or vice versa, which here we consider "primarily temperate" and "primarily tropical." Few indeed would be the species that truly span both in a

substantial way rather than outlier occurrences. Hence we believe a binary distinction is a reasonable approach given the scope and scale of this study.

3. The HiSSE hidden trait – any ideas what kind of hidden traits may relate to temperature and diversification and could be mentioned/discussed?

This is a great question. Of course, HiSSE models only provide statistical justification without much in the way of actual biology. Here, there are many unexplored climatic factors (e.g., precipitation, seasonality) and biotic factors (niche filling across communities, trait evolution and its cadence across groups) that are not directly considered but that should form exciting follow-up work. We argue that our HiSSE results are valuable because they point to the need for this next-step work.

Did you take topological and branch-length uncertainty into account in your analyses (e.g. run analyses over a set of trees)? This would be essential and it's unclear from the text whether this has been done.

We use the best likelihood tree among 352 bootstrap trees (cited in the manuscript as Sun et al., 2019: see bioRxiv 694950. doi: 10.1101/694950). It is possible for readers to understand the relative levels of uncertainty for clades by referring to files and text in Sun et al. (2019). For backbone nodes, our tree generally has strong support superior to recent phylogenetic studies in the group. However, this is admittedly indirect, and we do not explicitly integrate phylogenetic uncertainty statistically because the sheer scale of the clade unfortunately has made this computationally intractable. Many similar contributions to the literature have accounted for phylogenetic uncertainty, including some recent contributions from our group. However, this has generally been done on trees far smaller than ours. For a tree approaching 20,000 taxa, the run time on some of our single analyses has run into the territory of months (for example, Fabales has only 23.5% sampling, but it took us 60 days with three continuous runs of a total of 134,198,000 generations to reach convergence on a single tree topology, and even in this case the effective sample size, 230.9, is just past the usually recommended minimum of 200. The typical way phylogenetic uncertainty is run (for all of the analyses we implement, for instance, as BAMM, RPANDA, DR, etc. cannot consider multiple trees natively) is to run replicate analyses across bootstrap replicates and aggregate the result somehow. Running even a small number of replicate runs on bootstrap trees would have been burdensome and beyond even the strong computational resources we have. We

can say with quite a bit of certainty that no study has done this well (and often not at all; e.g. Rabosky et al. (2018): https://doi.org/10.1038/s41586-018-0273-1) at the phylogenetic scale we are working at. The best we have seen is Igea et al., 2020, Ecology Letters (mentioned above), who compared results between two different trees. While a binary comparison is a fairly weak approach for the goal of understanding phylogenetic uncertainty, we have already extensively studied the properties of this phylogenetic dataset across three trees in a separate manuscript (different from that cited above; due out in American Journal of Botany this year, 2020, cited in the manuscript as "Sun et al., 2020"; see bioRxiv Preprint, https://doi.org/10.1101/749325), hence we have already performed studies similar to those in Igea & Tanentzap (2020). We found similar results between the two molecular trees across the two datasets using BAMM, RPANDA, and DR, all implemented in a similar way to the work presented here. We believe our study of phylogenetic uncertainty is representative of the current literature standard at this phylogenetic scale.

With all this said, the reviewer has made a good point that we have incompletely discussed both phylogenetic uncertainty estimate challenges and our approach to this, and we have thus added some needed explanation in the main manuscript. That explanation includes describing computational intractability for fully incorporating uncertainty at the scale of the analysis here, and better discussing how results in the in press American Journal of Botany *paper (Sun et al., 2020, In press) provides needed context on comparative diversification rate analyses, as well as noting this is effectively similar to those implemented in Igea & Tanentzap (2020). This new text is now several prominent lines in the "Challenges" paragraph that closes the Discussion section.*

Minor comments:

L250 write out what DR and BAMM stand for

We have checked this area. These acronyms had already been defined at this point, and we detailed the definitions where they were initially introduced.

L264 Indicate the value of lambda – there is quite some discussion in the literature whether phylogenetic signal actually reflects a process such as phylogenetic niche conservatism. It would be good to mention this here, in the discussion or in the methods, and be careful with statements such as 'phylogenetic niche conservatism' (clearly, rosids made many transitions to temperate regions).

We agree with the reviewer and are now more circumspect in the manuscript. We have cited relevant literature on this point, very briefly in the Results and at greater length in the Methods. We note that "many transitions" and "niche conservatism" are not inherently contradictory as phylogenetic niche conservatism is a relative matter (as stated in the reviews we cite). It is possible to have some degree of phylogenetic niche conservatism (under some definitions) even with very high evolutionary rates.

L265 But STRAPP does not test for phylogenetic niche conservatism, or?

As written, this area of the manuscript was confusing, and we thank the reviewer for pointing this out. We revised the text, and further clarified that STRAPP was not used for testing phylogenetic niche conservatism.

L274/275 Again, indicate the value for lambda here

We have performed requested edits as noted above.

L340 Two times 'significantly' – remove one

We removed one "significantly".

L346 Indicate t-test

Not all the tests are carried as "t-test", so we indicated as suggested, as well as their corresponding references.

L359 This sounds like topicality – species in the tropics – is associated with high diversification, but that's not what you mean right? Maybe rephrase.

We thank the reviewer for pointing this out. We have checked and modified.

L364 An unobserved state, or an unobserved trait?

We thank the reviewer for the word suggestion. We have modified as "an unobserved trait" for clarity.

L482-483 Was it consistent with BAMM rate shifts, or did you actually correlate BAMM rates in the tree (so not tip-rates) with paleotemperature? I see in methods that this is done using regression models. There is of course the issue that a correlation is found because both are correlated with time

(temporal autocorrelation). This should be acknowledged at least (or corrected for). I would also be happy if you just indicate that the patterns are congruent, and there is thus a consistent relationship with the other tests performed, rather than doing a regression (otherwise people would repeat this approach and I do not think it's valid).

The correlation shown in the main text is between the BAMM tree-wide net diversification rates (not tip rates) and the paleo-temperature. We believe this is a reasonable approach in the spirit of summary statistics because we also present RPANDA analyses that explicitly rule out temporal autocorrelation as the only explanation of the result via model choice between temperature- and time-dependent models. Our options are to just rely on RPANDA for this analysis or to show results from multiple methods, not all of which are as explicitly able to deal with the autocorrelation issue. We have chosen the latter in the interest of demonstrating congruence among results.

Elaborating on our BAMM results, we found that more diversification rate shifts (97/182) were detected from 10-15 Myr to present, during which the global temperature was cooling, while <5 shifts were detected at the Eocene Thermal Maximum (ca. 55 Myr) when the global temperature was more than 5–8 °C above average. So the BAMM results agree with paleotemperature trends in several ways, not just via correlation.

In addition to the notes above, we also have incorporated these points in the main text and the Methods section to be explicit with readers about the limitations of this analysis.

Is there space for Fig. S3 in the main text? I think it's a strong figure.

We thank the reviewer for the endorsement of Fig. S3. We have moved it to the main text as Fig. 3.

Renske Onstein

REVIEWERS' COMMENTS:

Reviewer #3 (Remarks to the Author):

The authors addressed all my concerns in this revision and I do not have any further comments.
Looking forward to seeing this published.

Renske Onstein

REVIEWERS' COMMENTS: Reviewer #3 (Remarks to the Author):
The authors addressed all my concerns in this revision and I do not have any further comments. Looking forward to seeing this published.

Renske Onstein

We thank Dr. Onstein for multiple, careful and constructive reviews of this manuscript, leading to a greatly improved product.
